# Risk Factors for Weak Antibody Response of SARS-CoV-2 Vaccine in Adult Solid Organ Transplant Recipients: A Systemic Review and Meta-Analysis

**DOI:** 10.3389/fimmu.2022.888385

**Published:** 2022-06-14

**Authors:** Kezhen Zong, Dadi Peng, Hang Yang, Zuotian Huang, Yunhai Luo, Yihua Wang, Song Xiang, Tingting Li, Tong Mou, Zhongjun Wu

**Affiliations:** Department of Hepatobiliary Surgery, The First Affiliated Hospital of Chongqing Medical University, Chongqing, China

**Keywords:** organ transplantation, COVID19, SARS-CoV-2 vaccine, risk factors, antibody response, meta-analysis

## Abstract

**Objective:**

This is the first systematic review and meta-analysis to determine the factors that contribute to poor antibody response in organ transplant recipients after receiving the 2-dose severe acute respiratory syndrome coronavirus 2 (SARS-CoV-2) vaccine.

**Method:**

Data was obtained from Embase, PubMed, Web of Science, Cochrane Library, China National Knowledge Infrastructure (CNKI), and Chinese Biomedical Literature Database (CBM). Studies reporting factors associated with antibody responses to the 2-dose SARS-CoV-2 vaccine in solid organ transplant recipients were included in our study based on the inclusion and exclusion criteria. Two researchers completed the literature search, screening, and data extraction. Randomized models were used to obtain results. Egger’s test was performed to determine publication bias. Sensitivity analysis was performed to determine the stability of the result. The heterogeneity was determined using the Galbraith plot and subgroup analysis.

**Results:**

A total of 29 studies were included in the present study. The factors included living donor, BNT162b2, tacrolimus, cyclosporine, antimetabolite, mycophenolic acid (MPA) or mycophenolate mofetil (MMF), azathioprine, corticosteroids, high-dose corticosteroids, belatacept, mammalian target of rapamycin (mTOR) inhibitor, tritherapy, age, estimated glomerular filtration rate (eGFR), hemoglobin, and tacrolimus level were significantly different. Multivariate analysis showed significant differences in age, diabetes mellitus, MPA or MMF, high-dose corticosteroids, tritherapy, and eGFR.

**Conclusion:**

The possible independent risk factors for negative antibody response in patients with organ transplants who received the 2-dose SARS-CoV-2 vaccine include age, diabetes mellitus, low eGFR, MPA or MMF, high-dose corticosteroids, and triple immunosuppression therapy. mTOR inhibitor can be a protective factor against weak antibody response.

**Systematic Review Registration:**

PROSPERO, identifier CRD42021257965.

## Introduction

During the coronavirus disease (COVID-19) pandemic, severe acute respiratory syndrome coronavirus 2 (SARS-CoV-2) vaccination became important prophylaxis. The antibody response produced by the immunization can protect the body against SARS-CoV-2 (1–3). Previous studies have reported decreased antibody response after the administration of the SARS-CoV-2 vaccine in solid organ transplant recipients compared with that in healthy vaccinators (4, 5) who had a worse prognosis after infection (6–8). Identifying the risk factors associated with weak antibody response after vaccination can help further measures to increase the benefit of vaccination in solid organ transplant recipients. The present study focused on identifying factors that can affect the antibody response to the 2-dose SARS-CoV-2 vaccine in adults with solid organ transplants.

## Method

### Registration

The protocol of this systematic review and meta-analysis had been reported on the PROSPERO (registration no. CRD42021257965).

### Search Strategy

Electronic Databases used for searching included Embase, PubMed, Web of Science, Cochrane Library, China National Knowledge Infrastructure (CNKI), and Chinese Biomedical Literature Database (CBM). Few records were obtained from other sources (Studies were found to potentially meet the inclusion criteria before conducting the systematic search). The search strategy was conducted by combining subject terms with free words. Two investigators designed the search strategy collaboratively.

### Eligibility Criteria and Study Selection

Cohort studies, case-control studies, and case series were included in this study. The included population was defined as adult solid organ transplant recipients who received a standard dose of the SARS-CoV-2 vaccine. The inclusion criteria were as follows (1): study type: case-control, cohort studies, and case series (2); population: adult solid organ transplant recipients (3); intervention: 2-dose SARS-CoV-2 vaccine (4) outcome: the serologic results. The exclusion criteria were as follows (1): articles not published in Chinese or English language (2); no relevant factors reported (3); studies including the same population unless different outcomes were reported (4); recipients with a history of COVID19 (5); data not available to conduct the meta-analysis. Two examiners performed the study selection independently, with contradictions decided by negotiating with a third examiner.

### Data Collection and Extraction

Two researchers extracted the data separately, and different branches reached the decision together through discussion with a third researcher. Data were extracted from the general information about the study, population characteristics, the intervention method, serological assays, and potential influencing factors. Data that were believed to contain errors were not extracted and included. EndNote X9 was used for reference.

### Risk of Bias

Study quality was independently reviewed by two evaluators using the Newcastle Ottawa Scale (NOS), containing 3 main elements: selection, comparability, and outcome. The study quality was defined as follows: 0–3 points indicating low quality; 4–6 points indicating medium quality; 7–9 points indicating high quality. The Egger’s test was applied to assess publication bias, with P < 0.05 representing publication bias.

### Data Synthesis

Following the past protocol, a random-effect meta-analysis model was applied irrespective of heterogeneity. Standardized mean difference (SMD) with 95% confidence intervals was calculated for continuous variables, and odds ratio (OR) with 95% confidence intervals was applied for the dichotomous variables. Heterogeneity was evaluated using the Cochran Q test and I-squared, with I² > 50% or P < 0.1 considered to indicate high heterogeneity. Sensitivity analysis based on individual studies was conducted to examine the stability of the results. Subgroup analysis was conducted to explore the source of heterogeneity, which included the country, population, vaccine, and antibody for testing. Studies found to infer heterogeneity according to the Galbraith plot were excluded and then reanalyzed. OR originally reported by multivariate analysis was included in the analysis, but heterogeneity analysis and publication bias assessment was limited. The primary statistical analysis was performed by the StataSE 16 software.

## Results

### Literature Search and Study Characteristics

**Figure 1:**
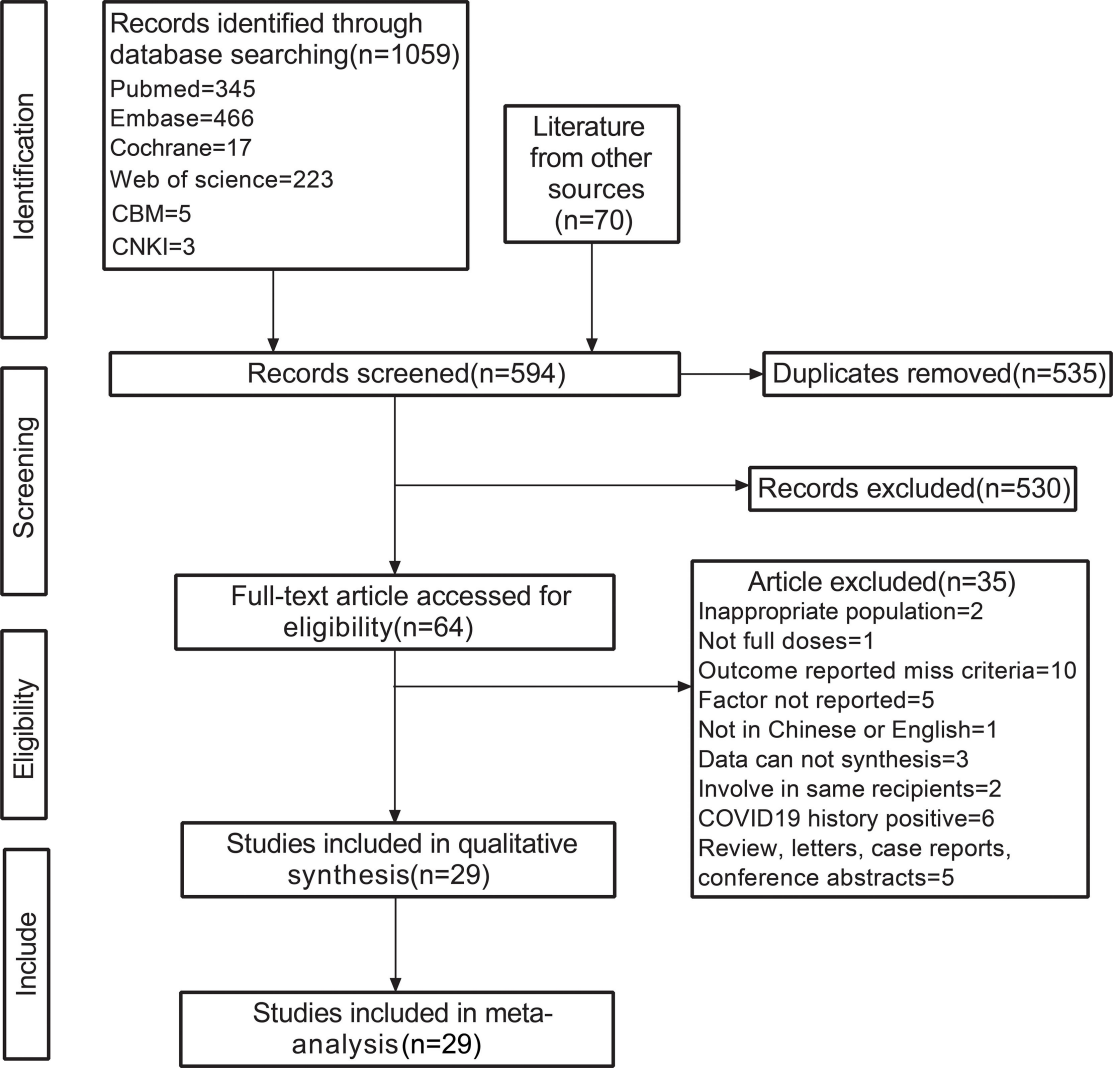
Flow chart depicting the study inclusion and exclusion processes. CNKI, China National Knowledge Infrastructure; CBM, Chinese Biomedical Literature Database; other sources, literature collected in other ways before conducting the systemic search.

The last literature search was performed on October 8, 2021. A total of 1129 records were obtained from electronic databases and other sources. The detailed search strategy is included in **Supplementary Material**. A total of 64 studies were included in the full-text review after initial screening and excluding duplicate studies. A total of 29 cohort studies (9–37) were finally included in the present study (**Figure 1**). Among the included studies, heart transplant recipients (HTR), liver transplant recipients (LTR), kidney transplant recipients (KTR), pulmonary transplant recipients (PTR), and other solid organ transplant recipients (SOTR) were involved, wherein KTR predominated. These individuals belonged from 11 different countries and received vaccination with four types of SARS-CoV-2 vaccines. Eventually, a total of 16 dichotomous variables, 11 continuous variables, and 10 OR values obtained from the multivariate analysis were combined in the analysis. The detailed study information and NOS score are present in the **Supplemental Material**.

### Data Analysis and Publication Bias

**Figure 2:**
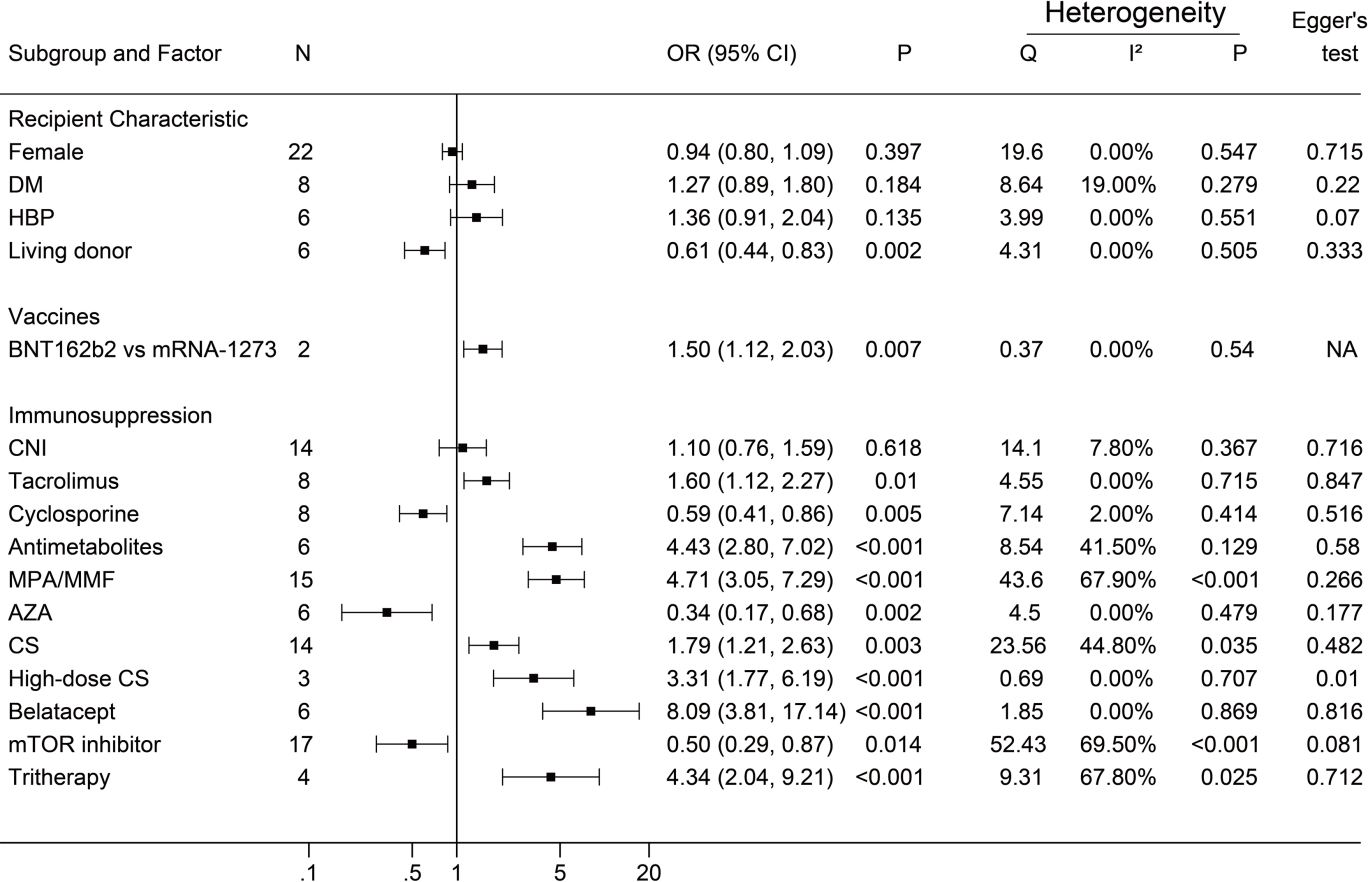
Forest plots of dichotomous factors. The event was a negative antibody response after vaccination. N, The number of studies; DM, diabetes mellitus; HBP, hypertension; CNI, calcineurin inhibitor; MPA/MMF, mycophenolic acid or mycophenolate mofetil; AZA, azathioprine; CS, low-dose corticosteroids; mTOR inhibitor, mammalian target of rapamycin inhibitor; Tritherapy, triple immunosuppressive therapy. NA indicates missing Egger's test.

**Figure 3:**
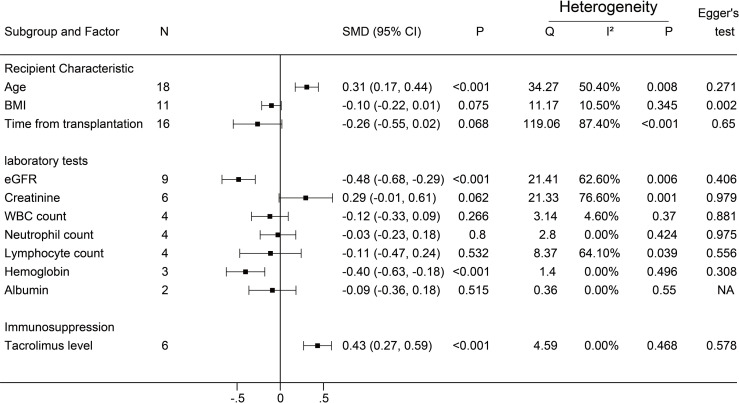
Forest plots of continuous factors.The event was a negative antibody response after vaccination. N, The number of studies; BMI, body mass index; eGFR, estimated glomerular filtration rate; WBC, white blood cells. NA indicates missing Egger's test.

**Figure 4:**
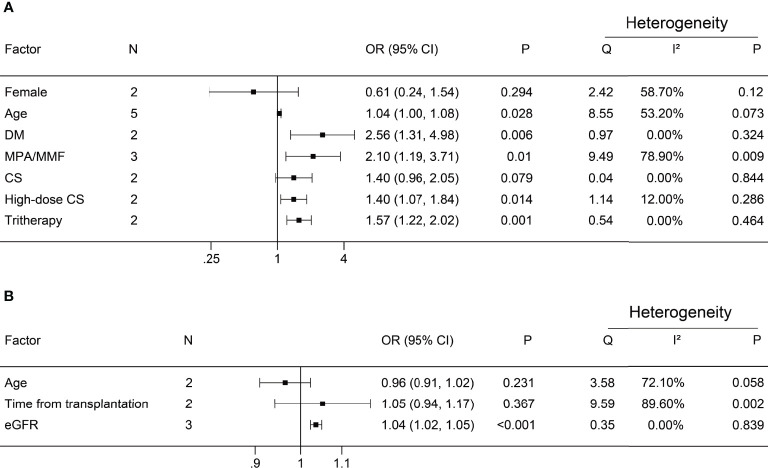
Forest plots of multivariate analysis. **(A)** Factors associated with a negative antibody response; **(B)** Factors associated with positive antibody response. N, number of studies; DM, diabetes mellitus; MPA/MMF, mycophenolic acid or mycophenolate mofetil; CS, corticosteroids; tritherapy, triple immunosuppressive therapy; eGFR, estimated glomerular filtration rate.

A negative antibody response was defined as an outcome event. The results of the dichotomous variables and the continuous variables are shown in **Figures 2** and **3**, respectively. The results of risk factors obtained from multivariate logistic regression analysis are shown in **Figure 4**. Significant differences were found among the dichotomous variables for twelve factors, which included living donor, BNT162b2, tacrolimus, cyclosporine, antimetabolite, mycophenolic acid (MPA)/mycophenolate mofetil (MMF), azathioprine (AZA), corticosteroids (CS), high-dose CS, belatacept, mammalian target of rapamycin (mTOR) inhibitor, and tritherapy. Significant heterogeneity was found in MPA/MMF, CS, mTOR inhibitor, and tritherapy. Egger’s test suggested publication bias for high-dose CS. Significant differences were found for the four continuous factors, which included age, estimated glomerular filtration rate (eGFR), hemoglobin, and tacrolimus level. Nevertheless, significant heterogeneity was found in age, time from transplantation, eGFR, creatinine, and lymphocyte count. Publication bias in body mass index (BMI) was determined by Egger’s test. The combined result from multivariate analysis associated with a negative response, age, diabetes mellitus, MPA/MMF, high-dose CS, and tritherapy showed significant differences. Significant heterogeneity was found in the female gender, age of the patient, and MPA/MMF. The only significant difference was in eGFR for factors associated with the positive response. Both age and time from transplantation showed significant heterogeneity.

### Heterogeneity and Exclusion Criteria

**Figure 5:**
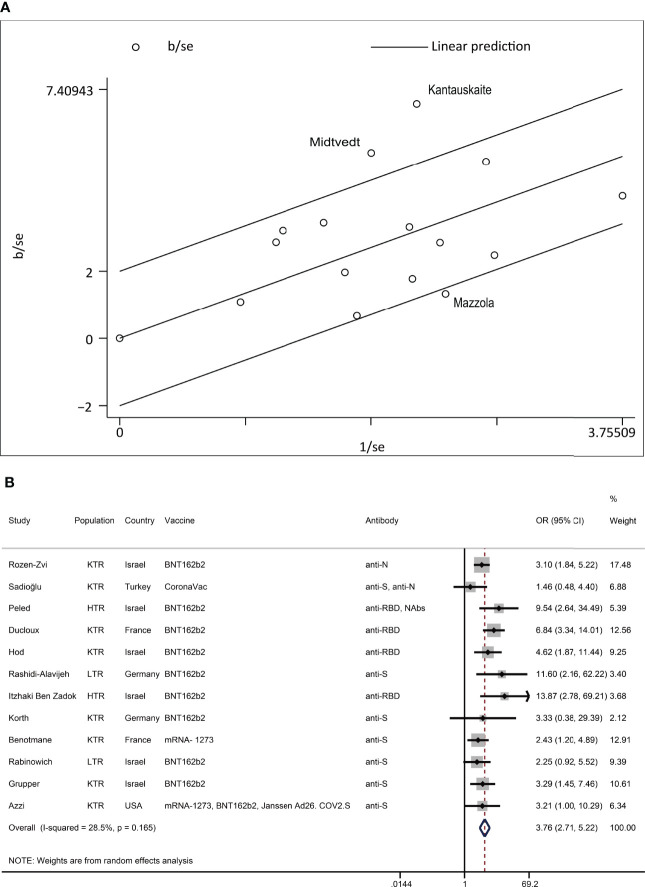
Heterogeneity exploring of MPA/MMF. **(A)** In the Galbraith plot of MPA/MMF for heterogeneity exploration, hollow circles outside the parallel reference line represent studies that were considered possible sources of heterogeneity. **(B)** Reanalysis of MPA/MMF after the removal of possible sources of heterogeneity. The event was a negative antibody response after vaccination. N, the number of studies; KTR, kidney transplant recipients; LTR, liver transplant recipients; HTR, heart transplant recipients; anti-S, anti-spike protein antibody; anti-N, anti-nucleocapsid protein antibody; anti-RBD, anti-receptor-binding domain antibody; Nab, neutralizing antibody.

**Figure 6:**
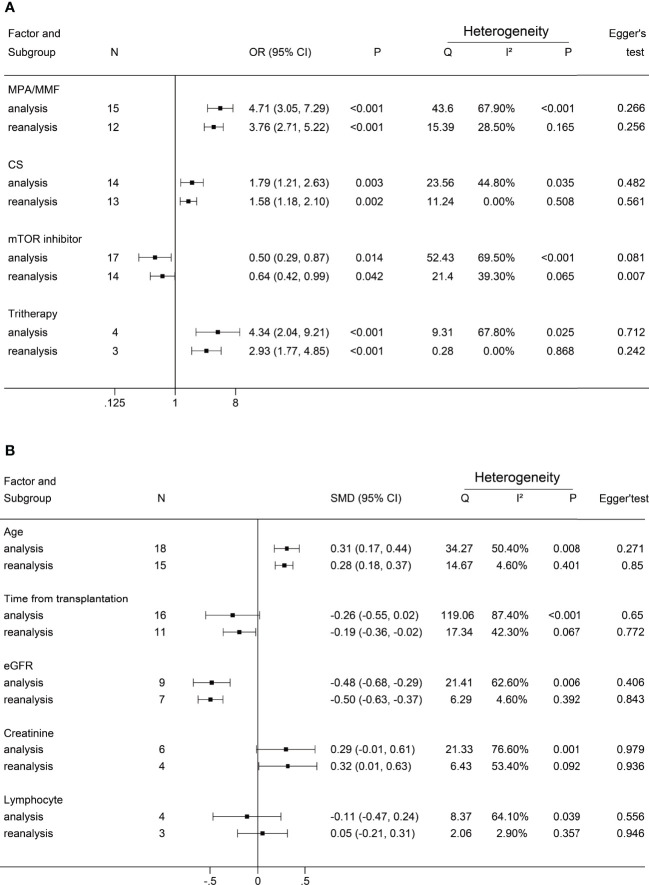
Forest plots of reanalysis. **(A)** reanalysis of dichotomous factors. **(B)** reanalysis of continuous factors. The event was a negative antibody response after vaccination. MPA/MMF, mycophenolic acid or mycophenolate mofetil; CS, corticosteroids; mTOR inhibitor, mammalian target of rapamycin inhibitor; Tritherapy, triple immunosuppressive therapy; eG.

We performed subgroup analyses and Galbraith plots to determine the sources of heterogeneity for dichotomous and continuous variables. In the present study, we found that I² >50% or P <0.1 was necessary for significant heterogeneity. The subgroup analysis based on country, population, vaccine, and antibody is shown in the **Supplemental Material**. Studies that can cause heterogeneity in results found by the Galbraith plot were excluded. The remaining studies were reanalyzed. Taking the factor MPA/MMF as an example, the results of the Galbraith plot and reanalysis are shown in **Figure 5**. The summary reanalysis results are shown in **Figure 6**. The studies that were excluded based on the Galbraith plot were as follows: MPA/MMF = 3 (28, 29, 33), CS = 1 (16), mTOR inhibitor = 3 (13, 15, 17), tritherapy = 1 (28), age = 3 (12, 18, 26), time from transplantation = 5 (11, 17, 21, 28, 35), eGFR = 2 (28, 31), creatinine = 2 (28, 30), and lymphocyte = 1 (34). Subgroup analyses and the Galbraith plot of other factors with significant heterogeneity, which included CS, mTOR inhibitor, tritherapy, age, time from transplantation, eGFR, creatinine, and lymphocyte, are shown in the **Supplemental Material**.

### Sensitivity Analysis

Sensitivity analysis of each factor showed that no individual studies changed the final results. The factors associated with multivariate analysis were not included in sensitivity analyses for a few included studies. The sensitivity analysis results are shown in the **Supplemental Material**.

## Discussion

To the best of our knowledge, this is the first systemic review and meta-analysis to determine the risk factors of negative antibody response to the 2-dose SARS-CoV-2 vaccine in adult recipients of solid organs. The significant risk factors associated with negative antibody response included age, diabetes mellitus, MPA or MMF, high-dose corticosteroids, and triple immunosuppression therapy.

The use of immunosuppressants is one of the most important reasons for anergy to vaccination. Our results showed that MPA/MMF, as a representative among antimetabolites, is an independent risk factor for negative antibody response after immunization. The study showed that the administration of MPA/MMF in patients with systemic lupus erythematosus (SLE) reduced the serologic response to the SARS-CoV-2 vaccine (38), which indicated that MMF/MPA weakened the humoral response after vaccination regardless of the receiver. Furthermore, in liver transplant recipients, mycophenolate not only resulted in a poor prognosis of COVID19 but also increased the risk of severe COVID-19 (39). These results implied that MMF/MPA should be used with caution during the COVID-19 pandemic. Corticosteroids are another widely used immunosuppressant that can reduce the antibody response after vaccination in solid organ transplant recipients with high doses versus low doses. Even though the result of dichotomous variables was different from the results of multivariate analysis, we believe that low-dose CS cannot increase the risk of nonresponse to vaccination because of the reliability of the multivariate analysis. Although reduced vaccine response with triple immunosuppressive therapy is dangerous, it is difficult to balance the immunostability and immune response when solid organ transplant recipients take triple immunosuppressants to keep their immune system silent.

Surprisingly, our study suggested another immunosuppressant, mTOR inhibitor, which did not increase the risk of vaccine ineffectiveness. This result was consistent with the multiple-logistic-regression results of Stumpf et al. and Ducloux et al. (17, 19). However, these results are difficult to synthesize. According to our results, dichotomous variables showed risk factors, whereas the opposite was found for cyclosporine. The results of the dichotomous variables (tacrolimus) were consistent with the continuous variables (tacrolimus level), which implied that tacrolimus can be negatively correlated with humoral immune response. The results show that belatacept can be associated with negative antibody response. Terrec et al. reported studies on COVID-19 infection and vaccine response after belatacept conversion in renal transplant recipients and concluded that renal transplant recipients receiving belatacept have a poorer response to the SARS-CoV-2 vaccine. This is consistent with our results, which suggested a delay in belatacept conversion because it can increase the risk of opportunistic infections (40).

The general characteristics of solid organ transplant recipients help to identify the high-risk population. Age is a risk factor for negative antibody response to SARS-CoV-2 vaccination. Many studies reported a late and weak response to the vaccine in elderly vaccinees than in younger vaccinees (41, 42), which is consistent with our results. However, we did not find a significant result for age in the multivariate analysis associated with positive antibody response. Diabetes mellitus is considered to be associated with negative antibody response because the multivariate analysis is more reliable. Moreover, meta-analysis results also indicated a negative correlation between diabetes mellitus prevalence and humoral response rate to the SARS-CoV-2 vaccine in other populations (43). Hypertension, another prevalent disease, was not a significant risk factor in our results. Living donors can be a protective factor for negative antibody responses, whereas administration of the BNT162b2 vaccine (compared to mRNA-1273) can be a risk factor. The result of multiple logistic regression also showed that BNT162b2 was an essential factor responsible for the failure of the antibody response in a previous study (19). The gender (female), BMI, and time from transplantation were not significant factors, which implied that they did not affect the antibody response.

The laboratory results from our study suggested that eGFR correlated with vaccine antibody response, which was shown by continuous variables and multivariate analyses. This indicated that patients with better renal function can react to the SARS-CoV-2 vaccine better. Unexpectedly, the results shown by hemoglobin indicated that it can be positively correlated with the antibody response. The reanalysis for creatinine showed a significant difference. However, we considered that the evidence for its correlation with the vaccine-antibody response was insufficient without the support of multivariate analysis (12, 32). However, according to our results, white blood cell (WBC) count, neutrophils count, lymphocytes count, and albumin did not show significance.

For heterogeneity determination, the subgroup analysis was unable to identify the causes of heterogeneity, which suggested that such subgroupings such as country, type of vaccine, antibody response, and type of transplantation may not be appropriate, and heterogeneity was because of a combination of multiple factors. After excluding the studies that caused heterogeneity found by the Galbraith plot, the heterogeneity of many factors became non-significant, and the results were also unchanged. However, the heterogeneity of several factors, such as mTOR inhibitor, time from transplantation, and creatinine levels, were significant after reanalysis, and the creatinine results changed, which suggested further investigating.

## Limitations

We did not limit the graft type of the included population because of the similarity of immune status and immunosuppressive agents in solid organ transplant recipients and also to include more studies to expand the conclusions. This led to heterogeneity. We selected the 2-dose-vaccine studies for intervention. However, studies have been performed to show that an increased dose can improve the immune response in solid organ transplant recipients. This was not included in our study (44, 45). We only considered the outcomes of antibody responses to the vaccine. We did not include cellular immune responses in the study; however, studies have reported that cellular immune responses were an equally important factor (46, 47). Among the factors included in the analysis, when constitutional ratios were not available, we considered calcineurin inhibitor (CNI) and antimetabolites, a class of drugs in general, as a variable. This can also contribute to the difficulty in interpreting some of our results.

Certain factors, such as eGFR and belatacept, were almost exclusively reported in renal transplantation studies. Some of the studies that fulfilled the inclusion criteria contained brief methods or process descriptions, which can increase the risk of errors. When potentially incorrect data were found, we excluded those parts of the data rather than the entire study. While determining heterogeneity, we identified that the study by Kantauskaite et al. was a source of heterogeneity in more than one factor. Therefore, we possibly had unreasonable inclusion and exclusion criteria for the study.

## Conclusions

Old age, diabetes mellitus, MPA or mycophenolate, high-dose corticosteroids, and triple immunosuppression therapy are important risk factors for negative antibody response in solid organ transplant recipients administered with a 2-dose SARS-CoV-2 vaccine. BNT162b2, antimetabolites, belatacept, and tacrolimus can be associated with weak antibody responses to the SARS-CoV-2 vaccine in solid organ transplant recipients, whereas the factor living donors showed opposite results. The administration of low-dose CS and mTOR inhibitor do not increase the risk of negative humoral response in solid organ transplant recipients receiving a 2-dose SARS-CoV-2 vaccine. In general, during the COVID-19 pandemic, MMF/MPA, high-dose CS, and triple immunosuppressants should be limited. For the population with risk factors, such as diabetes mellitus, old age, and low eGFR, the SARS-CoV-2 vaccine should be enhanced and more attention should be paid to preventing COVID-19 infection while keeping the vaccine safe. Compared with BNT162b2, the mRNA-1273 vaccine can be a better choice for solid organ transplant recipients. More advanced and comprehensive studies are required in the future.

## Data Availability Statement

The raw data supporting the conclusions of this article will be made available by the authors, without undue reservation.

## Author Contributions

Study design and registration: KZ and DP. Search strategy design: TL and TM. literature search: YW. Study selection: HY, YL, and KZ. Data collection and extraction: KZ, DP, and SX. Data analysis and writing: KZ, DP, and ZH. Arrangement and supervision: ZW. All authors contributed to the article and approved the submitted version.

## Funding

The present study was funded by The National Natural Science Foundation of China (No. 82170666 and No. 81873592).

## Conflict of Interest

The authors declare that the research was conducted in the absence of any commercial or financial relationships that could be construed as a potential conflict of interest.

## Publisher’s Note

All claims expressed in this article are solely those of the authors and do not necessarily represent those of their affiliated organizations, or those of the publisher, the editors and the reviewers. Any product that may be evaluated in this article, or claim that may be made by its manufacturer, is not guaranteed or endorsed by the publisher.

## References

[1] Corbett KS Nason MC Flach B Gagne M O'Connell S Johnston TS . Immune Correlates of Protection by mRNA-1273 Vaccine Against SARS-CoV-2 in Nonhuman Primates. Science (2021) 373(6561):eabj0299. doi: 10.1126/science.abj0299 34529476 PMC8449013

[2] He X Chandrashekar A Zahn R Wegmann F Yu J Mercado NB . Low-Dose Ad26.Cov2.S Protection Against SARS-CoV-2 Challenge in Rhesus Macaques. Cell (2021) 184(13):3467–73.e11. doi: 10.1016/j.cell.2021.05.040 34133941 PMC8166510

[3] McMahan K Yu J Mercado NB Loos C Tostanoski LH Chandrashekar A . Correlates of Protection Against SARS-CoV-2 in Rhesus Macaques. Nature (2021) 590(7847):630–4. doi: 10.1038/s41586-020-03041-6 PMC7906955 33276369

[4] Havlin J Svorcova M Dvorackova E Lastovicka J Lischke R Kalina T . Immunogenicity of Bnt162b2 mRNA Covid-19 Vaccine and SARS-CoV-2 Infection in Lung Transplant Recipients. J Heart Lung Transplant (2021) 40(8):754–8. doi: 10.1016/j.healun.2021.05.004 PMC8139179 34120839

[5] Sattler A Schrezenmeier E Weber UA Potekhin A Bachmann F Straub-Hohenbleicher H . Impaired Humoral and Cellular Immunity After SARS-CoV-2 Bnt162b2 (Tozinameran) Prime-Boost Vaccination in Kidney Transplant Recipients. J Clin Invest (2021) 131(14):e150175. doi: 10.1172/jci150175 PMC8279581 34101623

[6] Ao G Wang Y Qi X Nasr B Bao M Gao M . The Association Between Severe or Death Covid-19 and Solid Organ Transplantation: A Systematic Review and Meta-Analysis. Transplant Rev (Orlando) (2021) 35(3):100628. doi: 10.1016/j.trre.2021.100628 34087553 PMC8137345

[7] Ahmed F Abid M Maniya T Usman MS Fudim M . Incidence and Prognosis of Covid-19 Amongst Heart Transplant Recipients: A Systematic Review and Meta-Analysis. Eur J Prev Cardiol (2022) 29(6):e224–6. doi: 10.1093/eurjpc/zwab175 PMC8689985 34757386

[8] Caillard S Anglicheau D Matignon M Durrbach A Greze C Frimat L . An Initial Report From the French Sot Covid Registry Suggests High Mortality Due to Covid-19 in Recipients of Kidney Transplants. Kidney Int (2020) 98(6):1549–58. doi: 10.1016/j.kint.2020.08.005 PMC7444636 32853631

[9] Boyarsky BJ Werbel WA Avery RK Tobian AAR Massie AB Segev DL . Antibody Response to 2-Dose SARS-CoV-2 mRNA Vaccine Series in Solid Organ Transplant Recipients. Jama (2021) 325(21):2204–6. doi: 10.1001/jama.2021.7489 PMC8100911 33950155

[10] Bertrand D Hanoy M Edet S Lemée V Hamzaoui M Laurent C . Antibody Response to SARS-CoV-2 mRNA Bnt162b2 Vaccine in Kidney Transplant Recipients and in-Centre and Satellite Centre Haemodialysis Patients. Clin Kidney J (2021) 14(9):2127–8. doi: 10.1093/ckj/sfab100 PMC8344605 34471524

[11] Rozen-Zvi B Yahav D Agur T Zingerman B Ben-Zvi H Atamna A . Antibody Response to SARS-CoV-2 mRNA Vaccine Among Kidney Transplant Recipients: A Prospective Cohort Study. Clin Microbiol Infect (2021) 27(8):1173 e1– e4. doi: 10.1016/j.cmi.2021.04.028 PMC8091803 33957273

[12] Sadioğlu RE Demir E Evren E Aktar M Şafak S Artan AS . Antibody Response to Two Doses of Inactivated SARS-CoV-2 Vaccine (Coronavac) in Kidney Transplant Recipients. Transpl Infect Dis (2021) 23(6):e13740. doi: 10.1111/tid.13740 34606134 PMC8646873

[13] Peled Y Ram E Lavee J Sternik L Segev A Wieder-Finesod A . Bnt162b2 Vaccination in Heart Transplant Recipients: Clinical Experience and Antibody Response. J Heart Lung Transplant (2021) 40(8):759–62. doi: 10.1016/j.healun.2021.04.003 PMC8058049 34034958

[14] Herrera S Colmenero J Pascal M Escobedo M Castel MA Sole-González E . Cellular and Humoral Immune Response After mRNA-1273 SARS-CoV-2 Vaccine in Liver and Heart Transplant Recipients. Am J Transplant (2021) 21(12):3971–9. doi: 10.1111/ajt.16768 PMC9800111 34291552

[15] Shostak Y Shafran N Heching M Rosengarten D Shtraichman O Shitenberg D . Early Humoral Response Among Lung Transplant Recipients Vaccinated With Bnt162b2 Vaccine. Lancet Respir Med (2021) 9(6):e52–e3. doi: 10.1016/s2213-2600(21)00184-3 PMC8099313 33964244

[16] Rabinowich L Shibolet O Katchman H . Effectiveness of SARS-CoV-2 Vaccination in Liver Transplanted Patients: The Debate Is Open! J Hepatol (2022) 76(1):239–40. doi: 10.1016/j.jhep.2021.07.034 PMC8498774 34634386

[17] Ducloux D Colladant M Chabannes M Bamoulid J Courivaud C . Factors Associated With Humoral Response After Bnt162b2 mRNA Covid-19 Vaccination in Kidney Transplant Patients. Clin Kidney J (2021) 14(10):2270–2. doi: 10.1093/ckj/sfab125 PMC8483680 34603706

[18] Hall VG Ferreira VH Ierullo M Ku T Marinelli T Majchrzak-Kita B . Humoral and Cellular Immune Response and Safety of Two-Dose SARS-CoV-2 mRNA-1273 Vaccine in Solid Organ Transplant Recipients. Am J Transplant (2021) 21(12):3980–9. doi: 10.1111/ajt.16766 PMC8441872 34347934

[19] Stumpf J Siepmann T Lindner T Karger C Schwöbel J Anders L . Humoral and Cellular Immunity to SARS-CoV-2 Vaccination in Renal Transplant Versus Dialysis Patients: A Prospective, Multicenter Observational Study Using mRNA-1273 or Bnt162b2 mRNA Vaccine. Lancet Reg Health Eur (2021) 9:100178. doi: 10.1016/j.lanepe.2021.100178 34318288 PMC8299287

[20] Hod T Ben-David A Olmer L Levy I Ghinea R Mor E . Humoral Response of Renal Transplant Recipients to the BNT162b2 SARS-CoV-2 mRNA Vaccine Using Both RBD IgG and Neutralizing Antibodies. Transplantation (2021) 105(11):e234–43. doi: 10.1097/tp.0000000000003889 PMC8549122 34310101

[21] Rashidi-Alavijeh J Frey A Passenberg M Korth J Zmudzinski J Anastasiou OE . Humoral Response to SARS-CoV-2 Vaccination in Liver Transplant Recipients-A Single-Center Experience. Vaccines (Basel) (2021) 9(7):738. doi: 10.3390/vaccines9070738 34358154 PMC8310292

[22] Noble J Langello A Bouchut W Lupo J Lombardo D Rostaing L . Immune Response Post-SARS-CoV-2 mRNA Vaccination in Kidney-Transplant Recipients Receiving Belatacept. Transplantation (2021) 105(11):e259–60. doi: 10.1097/tp.0000000000003923 PMC8549124 34387243

[23] Ou MT Boyarsky BJ Chiang TPY Bae S Werbel WA Avery RK . Immunogenicity and Reactogenicity After SARS-CoV-2 mRNA Vaccination in Kidney Transplant Recipients Taking Belatacept. Transplantation (2021) 105(9):2119–23. doi: 10.1097/TP.0000000000003824 PMC8380692 34028386

[24] Marinaki S Adamopoulos S Degiannis D Roussos S Pavlopoulou ID Hatzakis A . Immunogenicity of SARS-CoV-2 Bnt162b2 Vaccine in Solid Organ Transplant Recipients. Am J Transplant (2021) 21(8):2913–5. doi: 10.1111/ajt.16607 PMC8250574 33864722

[25] Holden IK Bistrup C Nilsson AC Hansen JF Abazi R Davidsen JR . Immunogenicity of SARS-CoV-2 mRNA Vaccine in Solid Organ Transplant Recipients. J Intern Med (2021) 290(6):1264–7. doi: 10.1111/joim.13361 PMC8447120 34237179

[26] Itzhaki Ben Zadok O Shaul AA Ben-Avraham B Yaari V Ben Zvi H Shostak Y . Immunogenicity of the Bnt162b2 mRNA Vaccine in Heart Transplant Recipients - a Prospective Cohort Study. Eur J Heart Fail (2021) 23(9):1555–9. doi: 10.1002/ejhf.2199 33963635

[27] Korth J Jahn M Dorsch O Anastasiou OE Sorge-Hadicke B Eisenberger U . Impaired Humoral Response in Renal Transplant Recipients to SARS-CoV-2 Vaccination With Bnt162b2 (Pfizer-Biontech). Viruses (2021) 13(5):756. doi: 10.3390/v13050756 33923063 PMC8146144

[28] Kantauskaite M Müller L Kolb T Fischer S Hillebrandt J Ivens K . Intensity of Mycophenolate Mofetil Treatment Is Associated With an Impaired Immune Response to SARS-CoV-2 Vaccination in Kidney Transplant Recipients. Am J Transplant (2021) 22(2):634–9. doi: 10.1111/ajt.16851 PMC8653081 34551181

[29] Midtvedt K Tran T Parker K Marti HP Stenehjem AE Goransson LG . Low Immunization Rate in Kidney Transplant Recipients Also After Dose 2 of the Bnt162b2 Vaccine: Continue to Keep Your Guard Up! Transplantation (2021) 105(8):e80–e1. doi: 10.1097/TP.0000000000003856 PMC8294835 34132229

[30] Benotmane I Gautier-Vargas G Cognard N Olagne J Heibel F Braun-Parvez L . Low Immunization Rates Among Kidney Transplant Recipients Who Received 2 Doses of the mRNA-1273 SARS-CoV-2 Vaccine. Kidney Int (2021) 99(6):1498–500. doi: 10.1016/j.kint.2021.04.005 PMC8055921 33887315

[31] Rabinowich L Grupper A Baruch R Ben-Yehoyada M Halperin T Turner D . Low Immunogenicity to SARS-CoV-2 Vaccination Among Liver Transplant Recipients. J Hepatol (2021) 75(2):435–8. doi: 10.1016/j.jhep.2021.04.020 PMC8058047 33892006

[32] Crespo M Barrilado-Jackson A Padilla E Eguia J Echeverria-Esnal D Cao H . Negative Immune Responses to Two-Dose mRNA COVID-19 Vaccines in Renal Allograft Recipients Assessed With Simple Antibody and Interferon Gamma Release Assay Cellular Monitoring. Am J Transplant (2021) 22(3):786–800. doi: 10.1111/ajt.16854 34551187 PMC8653097

[33] Mazzola A Todesco E Drouin S Hazan F Marot S Thabut D . Poor Antibody Response After Two Doses of Severe Acute Respiratory Syndrome Coronavirus 2 (SARS-CoV-2) Vaccine in Transplant Recipients. Clin Infect Dis (2022) 74(6):1093–1096. doi: 10.1093/cid/ciab580 34166499 PMC8384412

[34] Grupper A Rabinowich L Schwartz D Schwartz IF Ben-Yehoyada M Shashar M . Reduced Humoral Response to mRNA SARS-CoV-2 Bnt162b2 Vaccine in Kidney Transplant Recipients Without Prior Exposure to the Virus. Am J Transplant (2021) 21(8):2719–26. doi: 10.1111/ajt.16615 PMC8250589 33866672

[35] Azzi Y Raees H Wang T Cleare L Liriano-Ward L Loarte-Campos P . Risk Factors Associated With Poor Response to Covid-19 Vaccination in Kidney Transplant Recipients. Kidney Int (2021) 100(5):1127–8. doi: 10.1016/j.kint.2021.08.019 PMC8413103 34481804

[36] Ruether DF Schaub GM Duengelhoef PM Haag F Brehm TT Fathi A . Sars-Cov2-Specific Humoral and T-Cell Immune Response After Second Vaccination in Liver Cirrhosis and Transplant Patients. Clin Gastroenterol Hepatol (2022) 20(1):162–72.e9. doi: 10.1016/j.cgh.2021.09.003 PMC8427908 34509643

[37] Russo G Lai Q Poli L Perrone MP Gaeta A Rossi M . SARS-CoV-2 Vaccination With Bnt162b2 in Renal Transplant Patients: Risk Factors for Impaired Response and Immunological Implications. Clin Transplant (2022) 36(1):e14495. doi: 10.1111/ctr.14495 34569101 PMC8646240

[38] Tang W Gartshteyn Y Ricker E Inzerillo S Murray S Khalili L . The Use of Covid-19 Vaccines in Patients With Sle. Curr Rheumatol Rep (2021) 23(11):79. doi: 10.1007/s11926-021-01046-2 34767100 PMC8586600

[39] Colmenero J Rodríguez-Perálvarez M Salcedo M Arias-Milla A Muñoz-Serrano A Graus J . Epidemiological Pattern, Incidence, and Outcomes of Covid-19 in Liver Transplant Patients. J Hepatol (2021) 74(1):148–55. doi: 10.1016/j.jhep.2020.07.040 PMC7395653 32750442

[40] Terrec F Jouve T Malvezzi P Janbon B Naciri Bennani H Rostaing L . Belatacept Use After Kidney Transplantation and Its Effects on Risk of Infection and Covid-19 Vaccine Response. J Clin Med (2021) 10(21):5159. doi: 10.3390/jcm10215159 34768680 PMC8585113

[41] Collier DA Ferreira I Kotagiri P Datir RP Lim EY Touizer E . Age-Related Immune Response Heterogeneity to SARS-CoV-2 Vaccine Bnt162b2. Nature (2021) 596(7872):417–22. doi: 10.1038/s41586-021-03739-1 PMC8373615 34192737

[42] Chen WH Kozlovsky BF Effros RB Grubeck-Loebenstein B Edelman R Sztein MB . Vaccination in the Elderly: An Immunological Perspective. Trends Immunol (2009) 30(7):351–9. doi: 10.1016/j.it.2009.05.002 PMC3739436 19540808

[43] Chen JJ Lee TH Tian YC Lee CC Fan PC Chang CH . Immunogenicity Rates After SARS-CoV-2 Vaccination in People With End-Stage Kidney Disease: A Systematic Review and Meta-Analysis. JAMA Netw Open (2021) 4(10):e2131749. doi: 10.1001/jamanetworkopen.2021.31749 34709385 PMC8554642

[44] Benotmane I Gautier G Perrin P Olagne J Cognard N Fafi-Kremer S . Antibody Response After a Third Dose of the mRNA-1273 SARS-CoV-2 Vaccine in Kidney Transplant Recipients With Minimal Serologic Response to 2 Doses. JAMA (2021) 326(11):1063–5. doi: 10.1001/jama.2021.12339 PMC8456389 34297036

[45] Westhoff TH Seibert FS Anft M Blazquez-Navarro A Skrzypczyk S Zgoura P . A Third Vaccine Dose Substantially Improves Humoral and Cellular SARS-CoV-2 Immunity in Renal Transplant Recipients With Primary Humoral Nonresponse. Kidney Int (2021) 100(5):1135–6. doi: 10.1016/j.kint.2021.09.001 PMC8427909 34509489

[46] Dolff S Zhou B Korth J Luo D Dai Y Jahn M . 21evidence of Cell-Mediated Immune Response in Kidney Transplants With a Negative mRNA Vaccine Antibody Response. Kidney Int (2021) 100(2):479–80. doi: 10.1016/j.kint.2021.05.013 PMC8141266 34029553

[47] Lovatt J Lam S Carter C Nadat F Moore J Chimakurthi CR . T Cell Response in Immunosuppressed Redo-Liver Transplant Recipients After Covid-19 Infection and After First Dose of Vaccine Against SARS-CoV-2. Transplantation (2021) 105(8 SUPPL 1):156. doi: 10.1097/01.tp.0000789500.50801.c7

