# Supplementary material for: Risk Factors for Weak Antibody Response of SARS-CoV-2 Vaccine in Adult Solid Organ Transplant Recipients: A Systemic Review and Meta-Analysis
Source: Front Immunol. 2022 Jun 14;13:888385. doi: 10.3389/fimmu.2022.888385 (PMC9237843; doi:10.3389/fimmu.2022.888385)

Supplementary Material

# Search Strategy

Cochrane

| #1 | MeSH descriptor: [Transplantation] explode all trees |
| --- | --- |
| #2 | (Transplant*):ti,ab,kw |
| #3 | #1 OR #2 |
| #4 | (Vaccine*):ti,ab,kw OR (Vaccinat*):ti,ab,kw |
| #5 | (COVID-19):ti,ab,kw OR (COVID 19):ti,ab,kw OR (COVID19):ti,ab,kw OR (SARS-CoV-2):ti,ab,kw OR (SARS CoV 2):ti,ab,kw OR (SARS2):ti,ab,kw OR (Coronavirus Disease 2019):ti,ab,kw OR (Coronavirus Disease-19):ti,ab,kw OR (Coronavirus Disease 19):ti,ab,kw OR (2019 nCoV):ti,ab,kw OR (2019 Novel Coronavirus):ti,ab,kw OR (SARS Coronavirus 2):ti,ab,kw |
| #6 | MeSH descriptor: [COVID-19 Vaccines] explode all trees |
| #7 | #4 AND #5 |
| #8 | #6 OR #7 |
| #9 | #3 AND #8 |
|  |  |

# Pubmed

| #1 | ("Transplantation"[Mesh]) OR (Transplant*[Title/Abstract]) | "Transplantation"[MeSH Terms] OR "transplant*"[Title/Abstract] |
| --- | --- | --- |
| #2 | (Vaccine*[Title/Abstract]) OR (Vaccinat*[Title/Abstract]) | "vaccine*"[Title/Abstract] OR "vaccinat*"[Title/Abstract] |
| #3 | ((((((((((((COVID-19[Title/Abstract]) OR (COVID 19[Title/Abstract])) OR (COVID19[Title/Abstract])) OR (SARS-CoV-2[Title/Abstract])) OR (SARS CoV 2[Title/Abstract])) OR (SARS2[Title/Abstract])) OR (Coronavirus Disease 2019[Title/Abstract])) OR (Coronavirus Disease-19[Title/Abstract])) OR (Coronavirus Disease 19[Title/Abstract])) OR (2019-nCoV[Title/Abstract])) OR (2019 nCoV[Title/Abstract])) OR (2019 Novel Coronavirus[Title/Abstract])) OR (SARS Coronavirus 2[Title/Abstract]) | "COVID-19"[Title/Abstract] OR "COVID-19"[Title/Abstract] OR "COVID19"[Title/Abstract] OR "SARS-CoV-2"[Title/Abstract] OR "SARS-CoV-2"[Title/Abstract] OR "SARS2"[Title/Abstract] OR "coronavirus disease 2019"[Title/Abstract] OR "coronavirus disease 19"[Title/Abstract] OR "coronavirus disease 19"[Title/Abstract] OR "2019-nCoV"[Title/Abstract] OR "2019-nCoV"[Title/Abstract] OR "2019 novel coronavirus"[Title/Abstract] OR "sars coronavirus 2"[Title/Abstract] |
| #4 | (#2) AND (#3) | ("vaccine*"[Title/Abstract] OR "vaccinat*"[Title/Abstract]) AND ("COVID-19"[Title/Abstract] OR "COVID-19"[Title/Abstract] OR "COVID19"[Title/Abstract] OR "SARS-CoV-2"[Title/Abstract] OR "SARS-CoV-2"[Title/Abstract] OR "SARS2"[Title/Abstract] OR "coronavirus disease 2019"[Title/Abstract] OR "coronavirus disease 19"[Title/Abstract] OR "coronavirus disease 19"[Title/Abstract] OR "2019-nCoV"[Title/Abstract] OR "2019-nCoV"[Title/Abstract] OR "2019 novel coronavirus"[Title/Abstract] OR "sars coronavirus 2"[Title/Abstract]) |
| #5 | ("COVID-19 Vaccines"[Mesh]) OR (#4) | "COVID-19 Vaccines"[MeSH Terms] OR (("vaccine*"[Title/Abstract] OR "vaccinat*"[Title/Abstract]) AND ("COVID-19"[Title/Abstract] OR "COVID-19"[Title/Abstract] OR "COVID19"[Title/Abstract] OR "SARS-CoV-2"[Title/Abstract] OR "SARS-CoV-2"[Title/Abstract] OR "SARS2"[Title/Abstract] OR "coronavirus disease 2019"[Title/Abstract] OR "coronavirus disease 19"[Title/Abstract] OR "coronavirus disease 19"[Title/Abstract] OR "2019-nCoV"[Title/Abstract] OR "2019-nCoV"[Title/Abstract] OR "2019 novel coronavirus"[Title/Abstract] OR "sars coronavirus 2"[Title/Abstract])) |
| #6 | (#1) AND (#5) | ("Transplantation"[MeSH Terms] OR "transplant*"[Title/Abstract]) AND ("COVID-19 Vaccines"[MeSH Terms] OR (("vaccine*"[Title/Abstract] OR "vaccinat*"[Title/Abstract]) AND ("COVID-19"[Title/Abstract] OR "COVID-19"[Title/Abstract] OR "COVID19"[Title/Abstract] OR "SARS-CoV-2"[Title/Abstract] OR "SARS-CoV-2"[Title/Abstract] OR "SARS2"[Title/Abstract] OR "coronavirus disease 2019"[Title/Abstract] OR "coronavirus disease 19"[Title/Abstract] OR "coronavirus disease 19"[Title/Abstract] OR "2019-nCoV"[Title/Abstract] OR "2019-nCoV"[Title/Abstract] OR "2019 novel coronavirus"[Title/Abstract] OR "sars coronavirus 2"[Title/Abstract]))) |

# Embase

| #1 | transplant*:ti,ab,kw |
| --- | --- |
| #2 | 'transplantation'/exp |
| #3 | vaccine*:ti,ab,kw OR vaccinat*:ti,ab,kw |
| #4 | 'covid 19':ti,ab,kw OR covid19:ti,ab,kw OR 'sars cov 2':ti,ab,kw OR sars2:ti,ab,kw OR 'coronavirus disease 2019':ti,ab,kw OR 'coronavirus disease-19':ti,ab,kw OR 'coronavirus disease 19':ti,ab,kw OR '2019 ncov':ti,ab,kw OR '2019 novel coronavirus':ti,ab,kw OR 'sars coronavirus 2':ti,ab,kw |
| #5 | 'sars-cov-2 vaccine'/exp |
| #6 | #1 OR #2 |
| #7 | #3 AND #4 |
| #8 | #5 OR #7 |
| #9 | #6 AND #8 |

Web of Science

| #1 | Transplant* (Topic) |
| --- | --- |
| #2 | Vaccine* (Topic) or Vaccinat* (Topic) |
| #3 | COVID-19(Topic) or COVID 19 (Topic) or COVID19 (Topic) or SARS-CoV-2 (Topic) or SARS CoV 2 (Topic) or SARS2 (Topic) or Coronavirus Disease 2019(Topic) or Coronavirus Disease-19 (Topic) or Coronavirus Disease 19(Topic) or 2019-nCoV(Topic) or 2019 nCoV (Topic) or 2019 Novel Coronavirus (Topic) or SARS Coronavirus 2(Topic) |
| #4 | ((#1) AND #2) AND #3 |

# Supplementary Table and Figure

Study Characteristics and NOS score


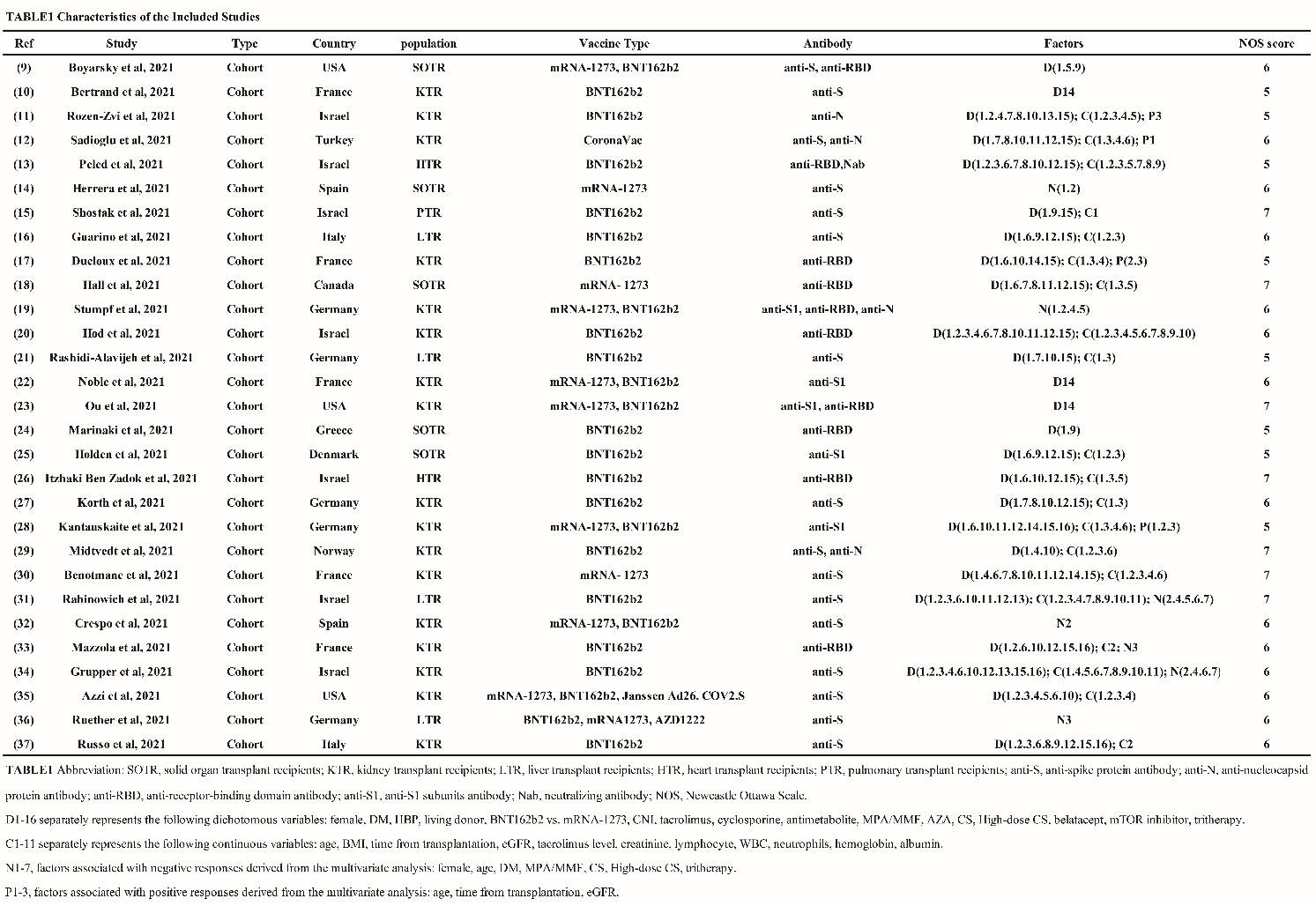


Subgroup analysis based on country, population, vaccine, and antibody


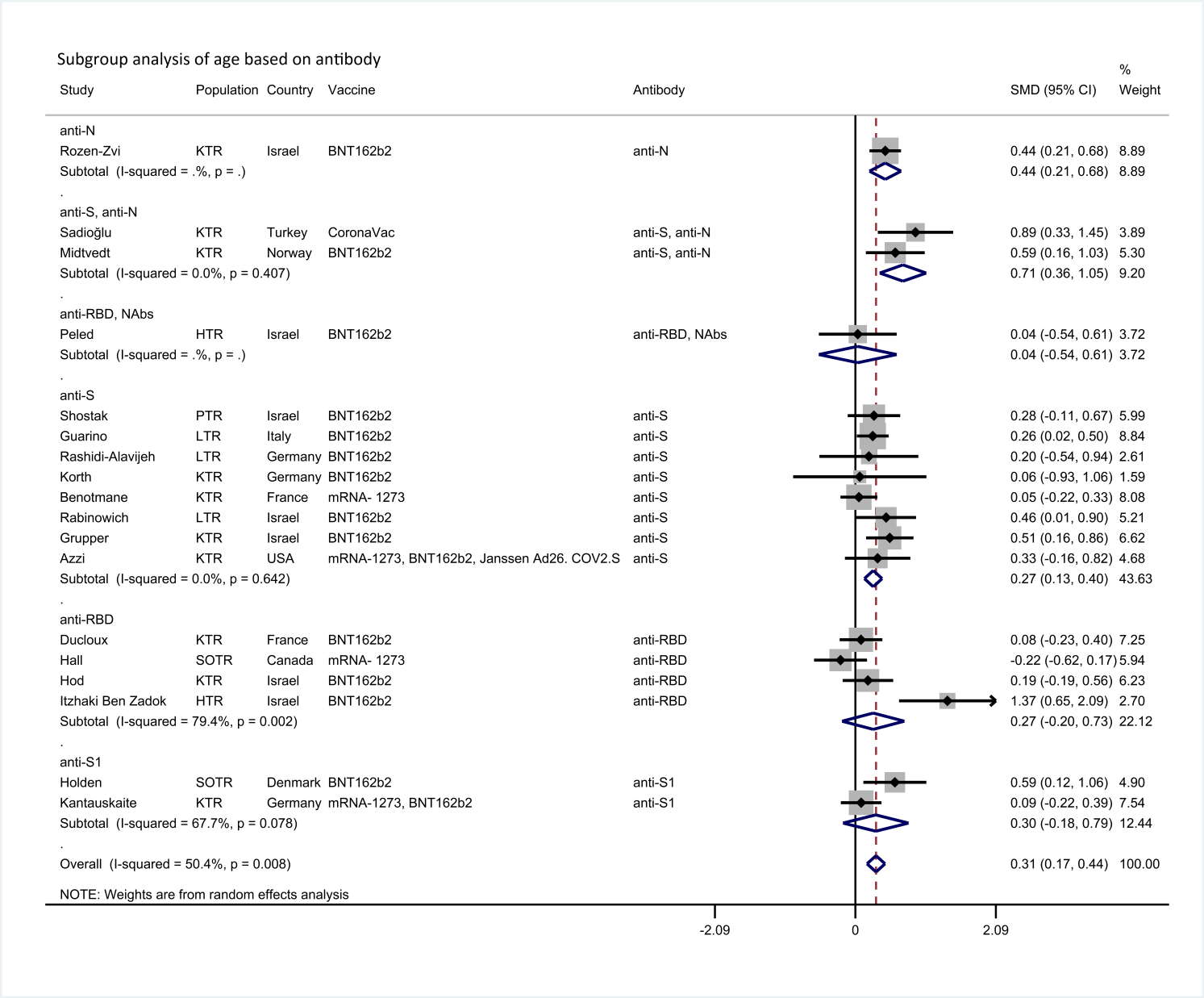

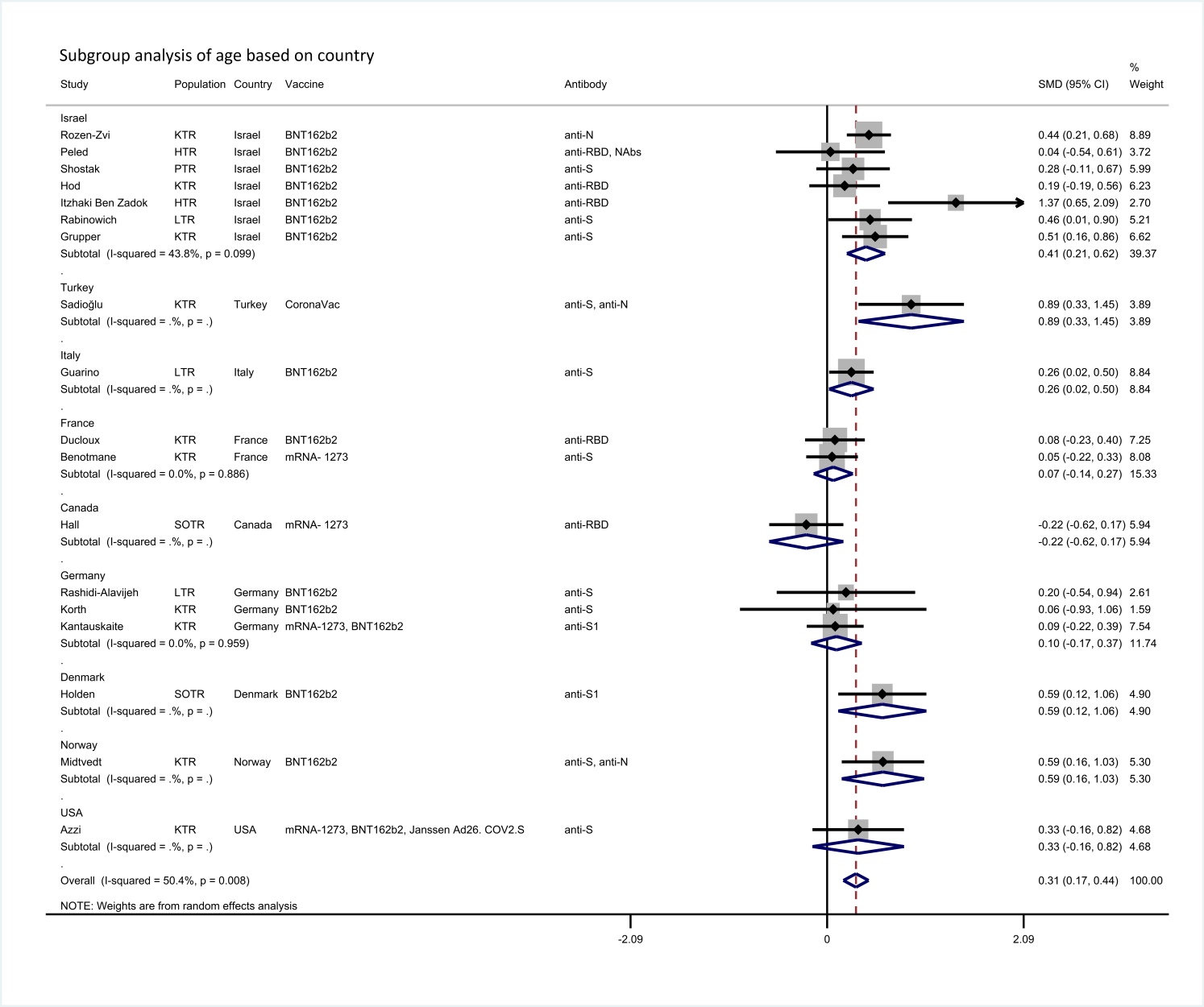

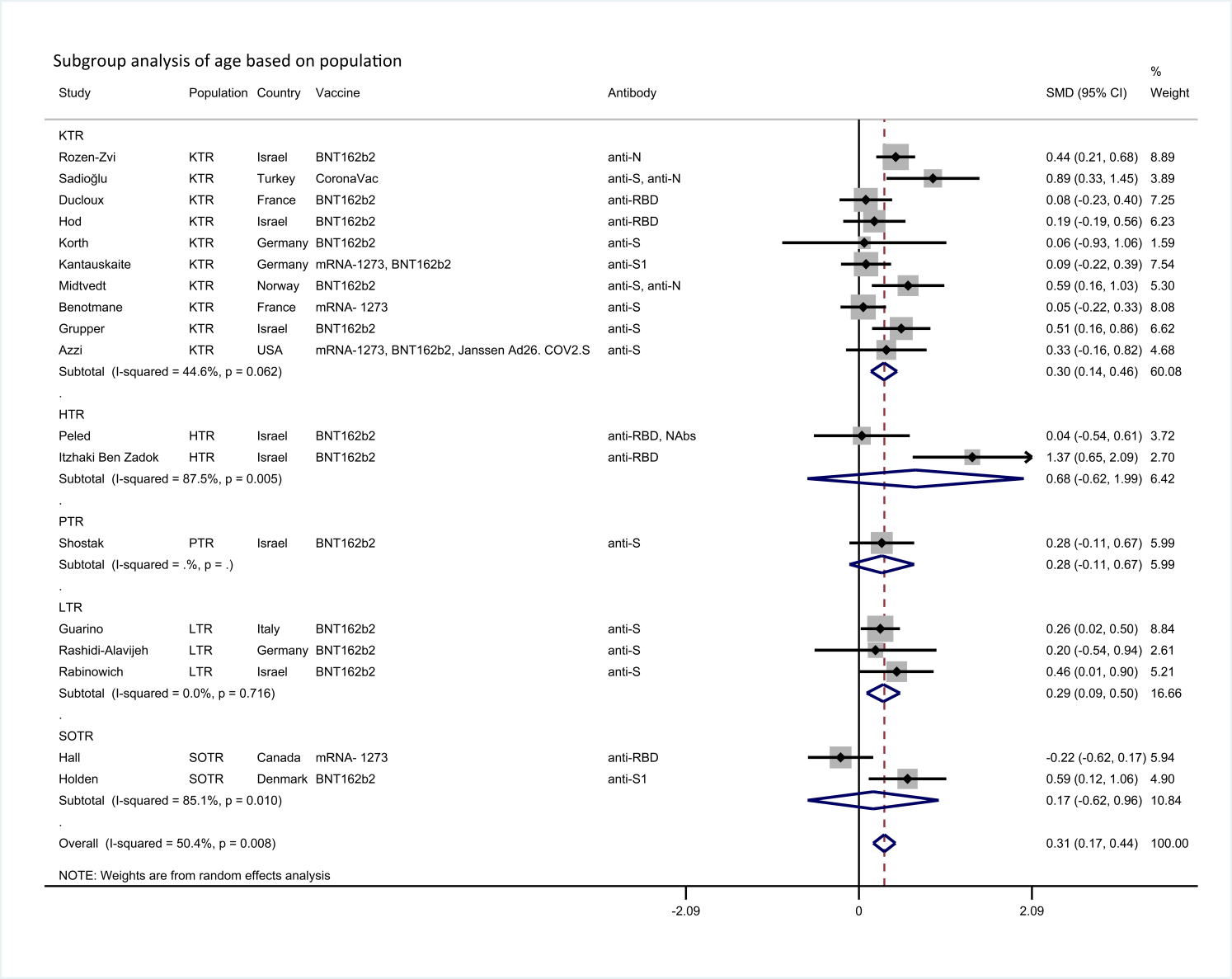

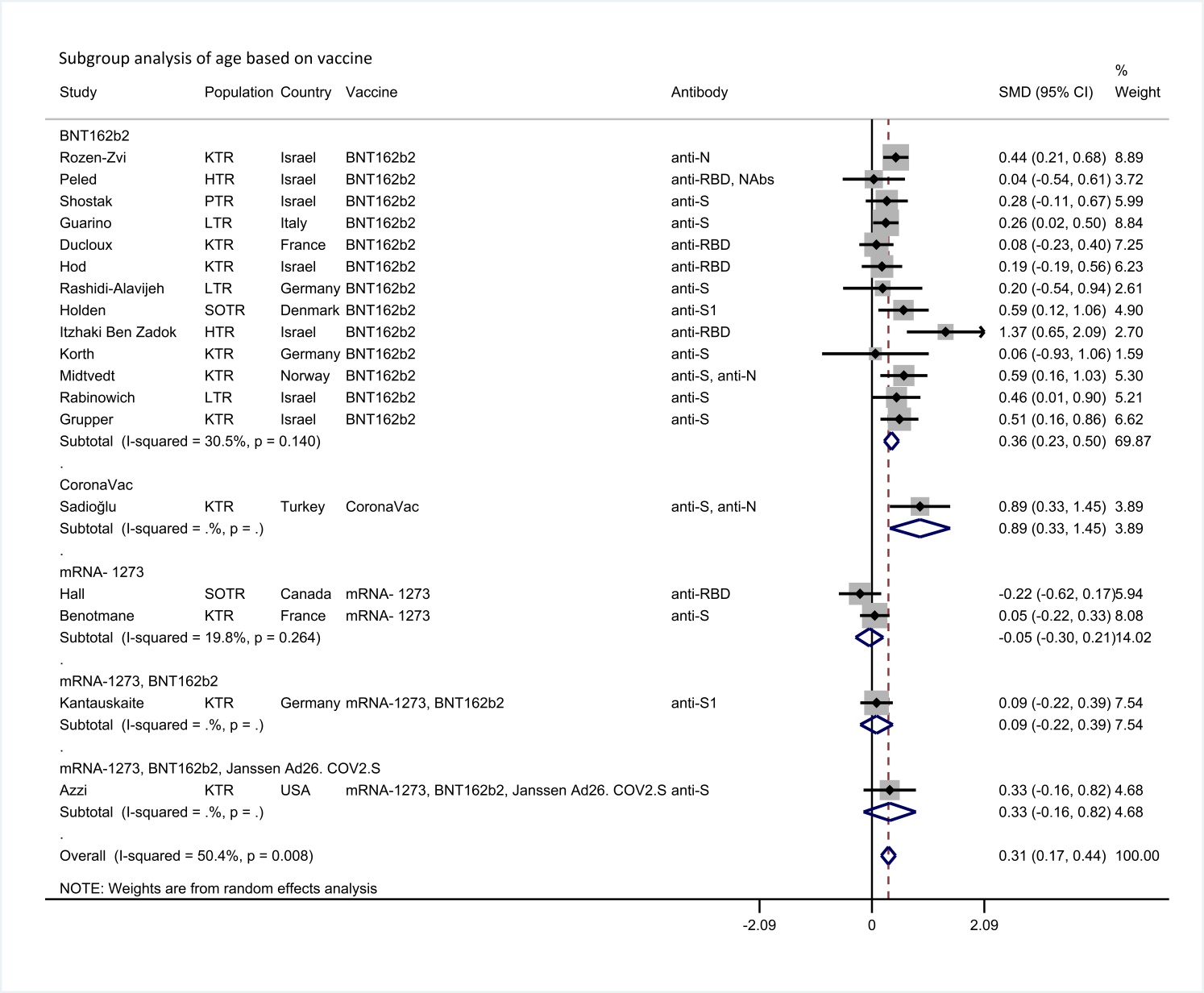


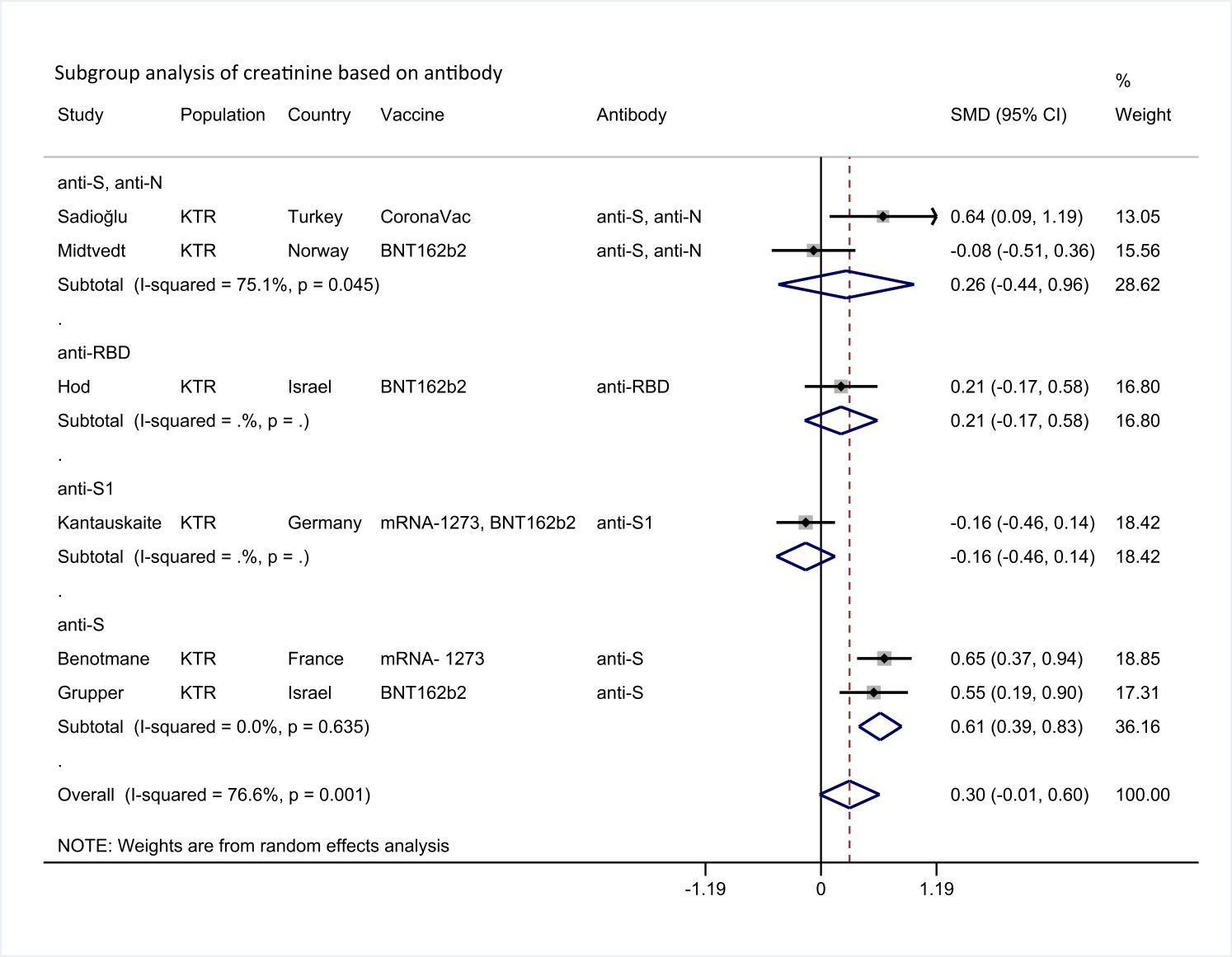

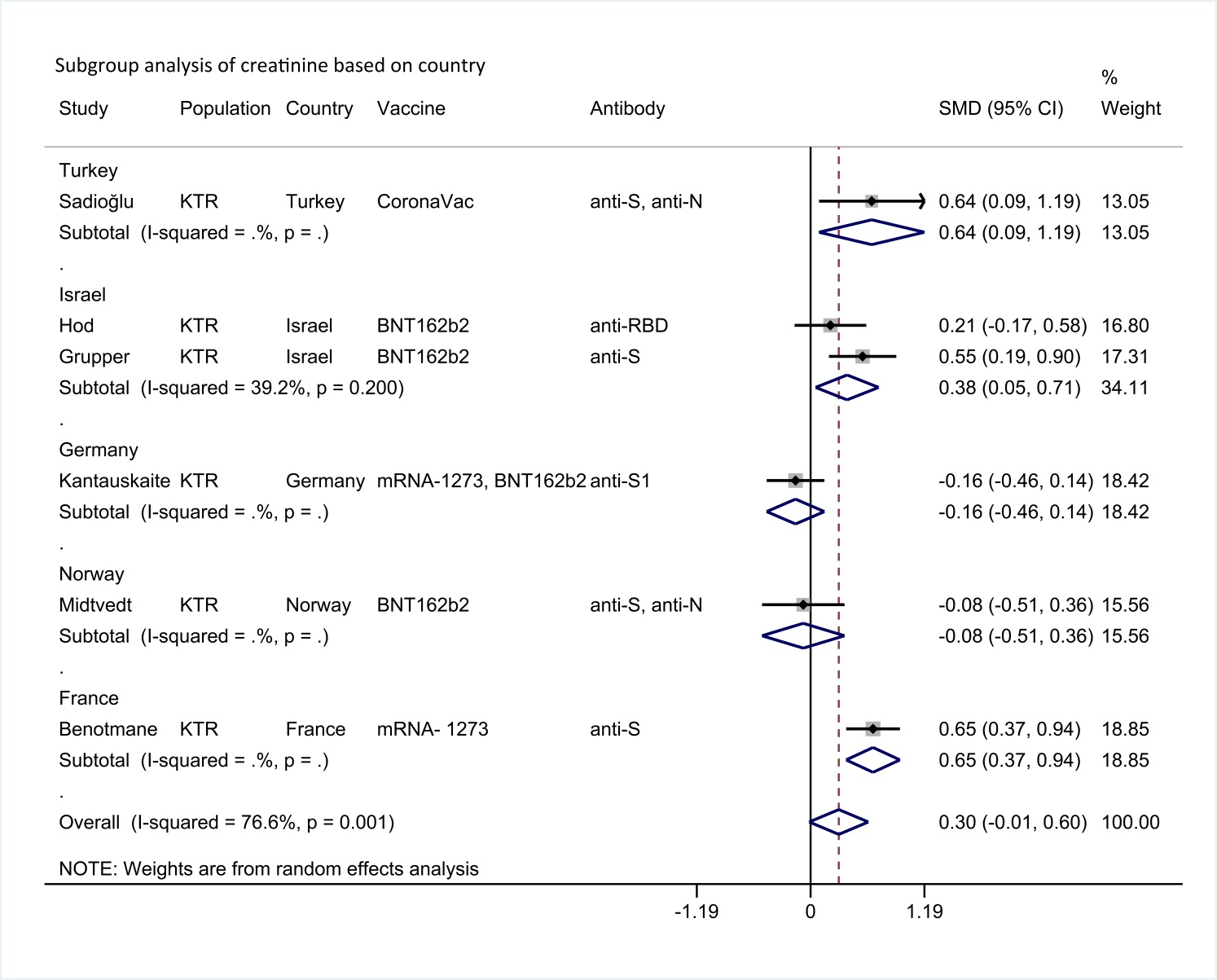

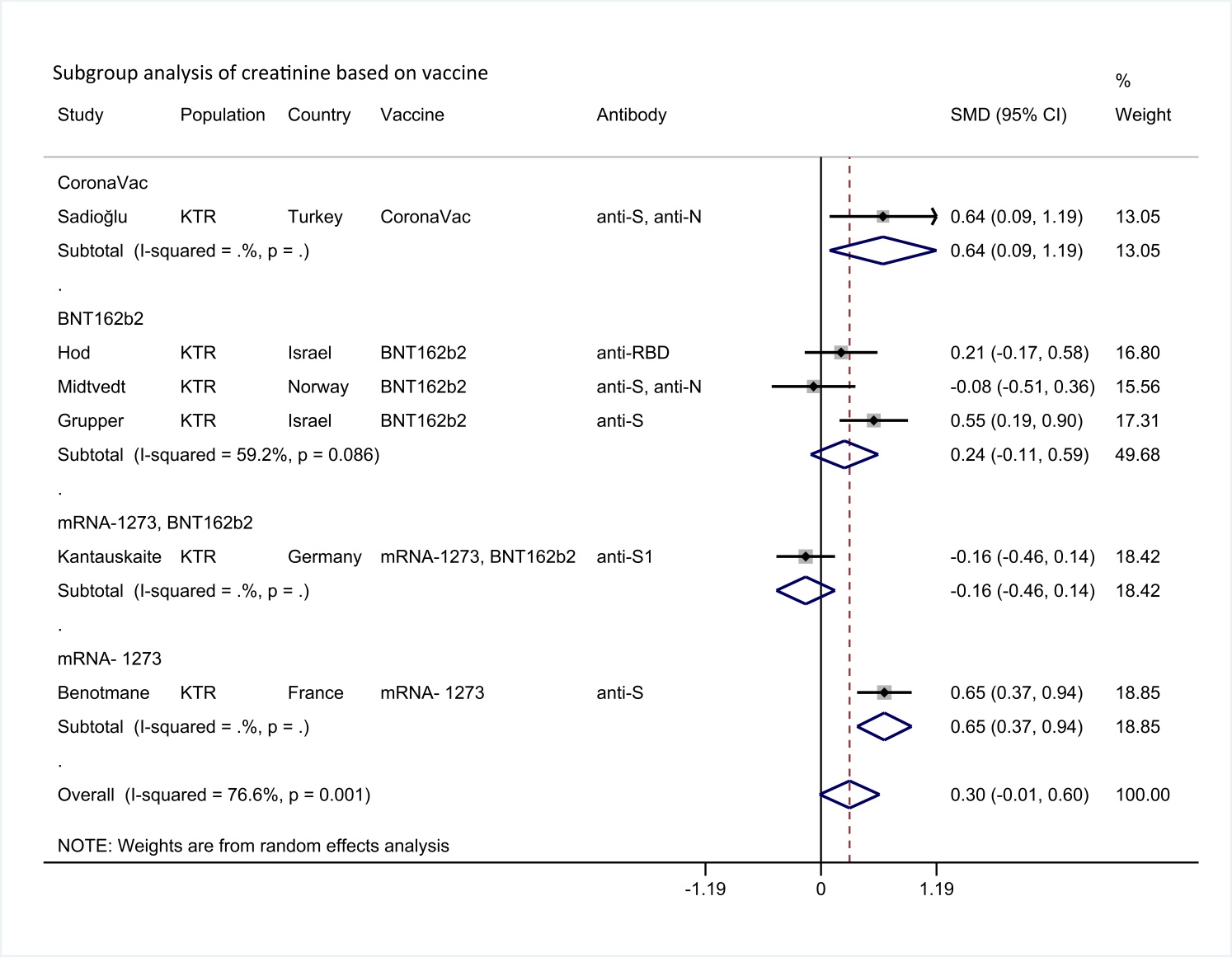


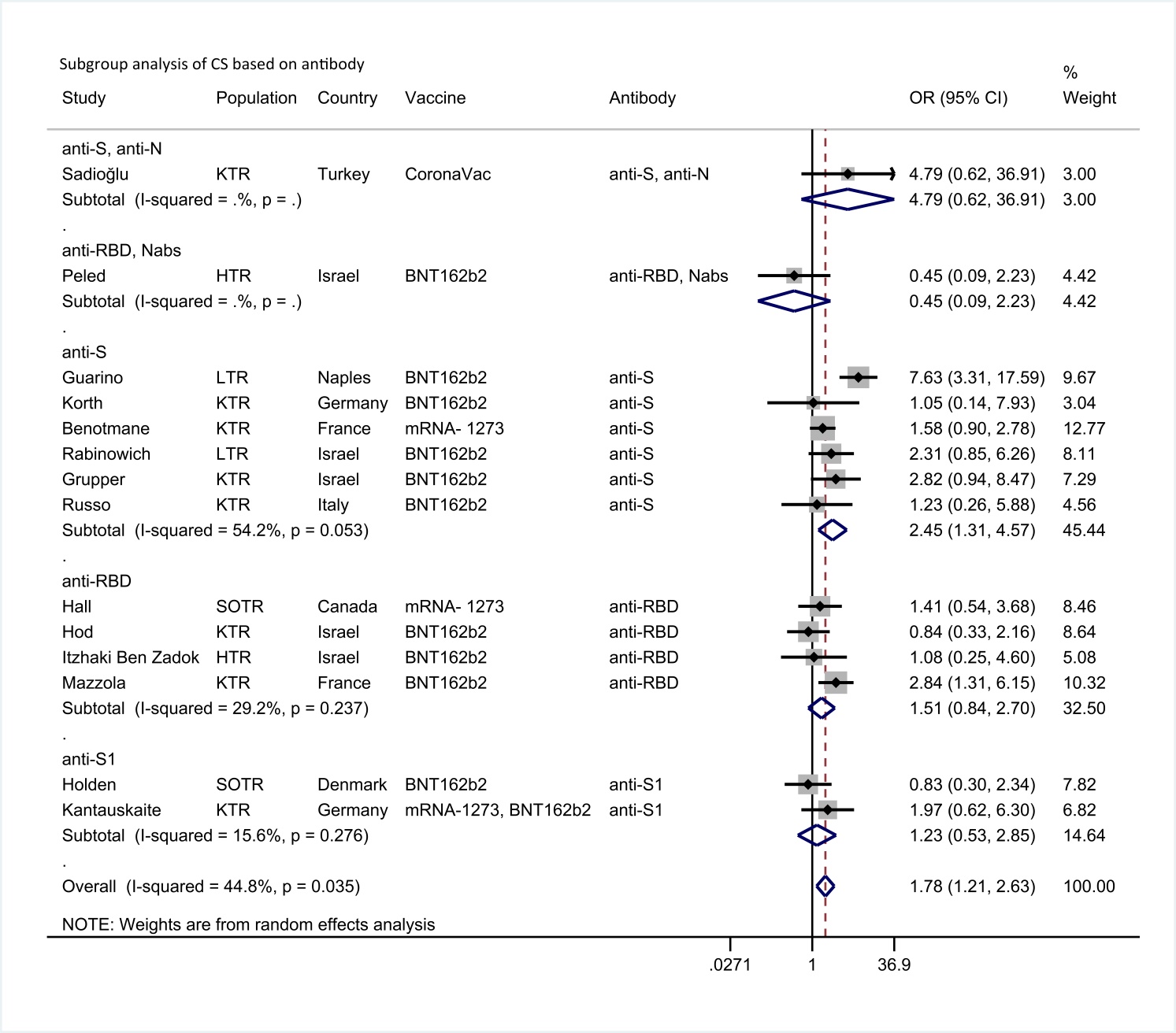

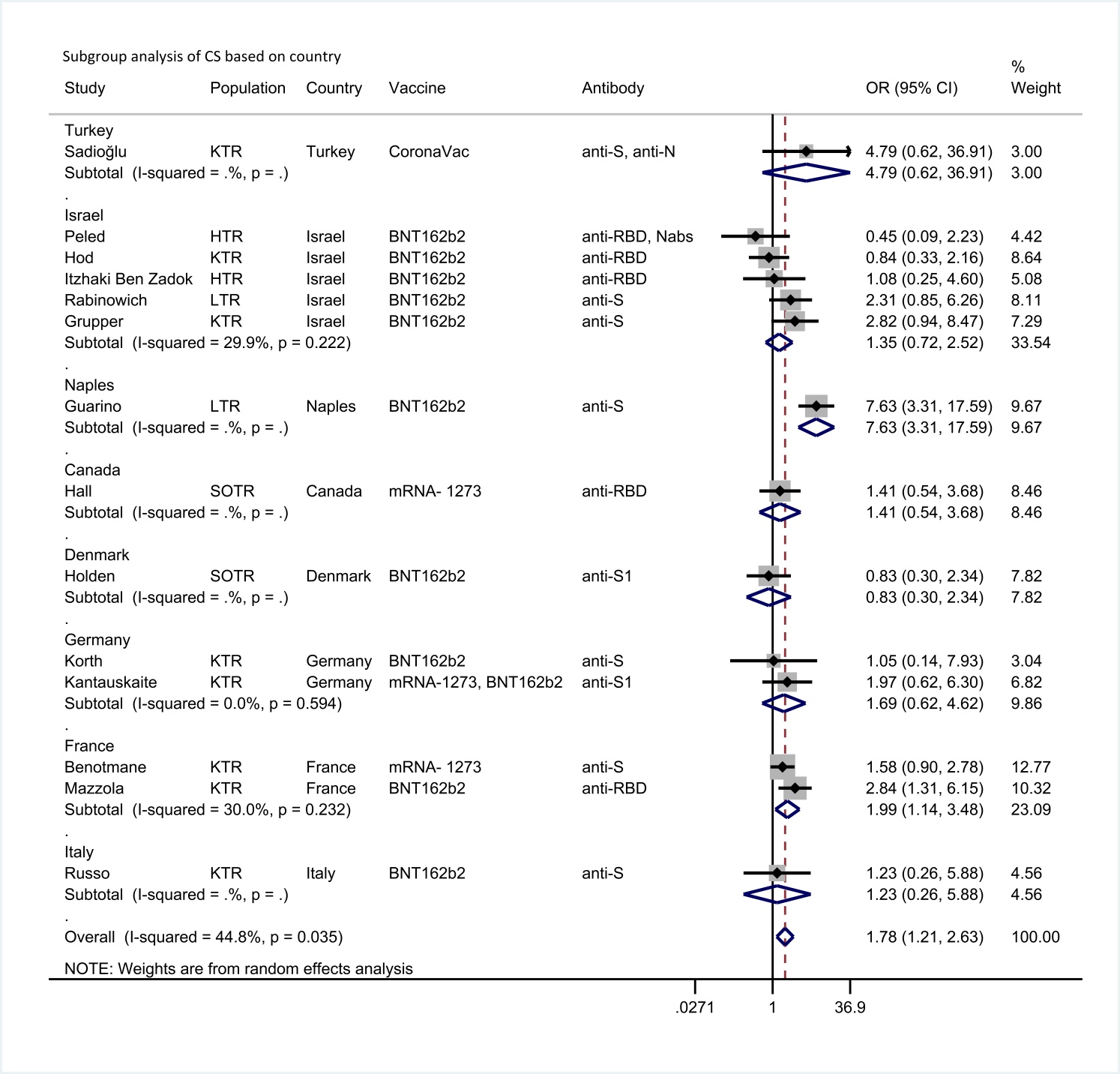

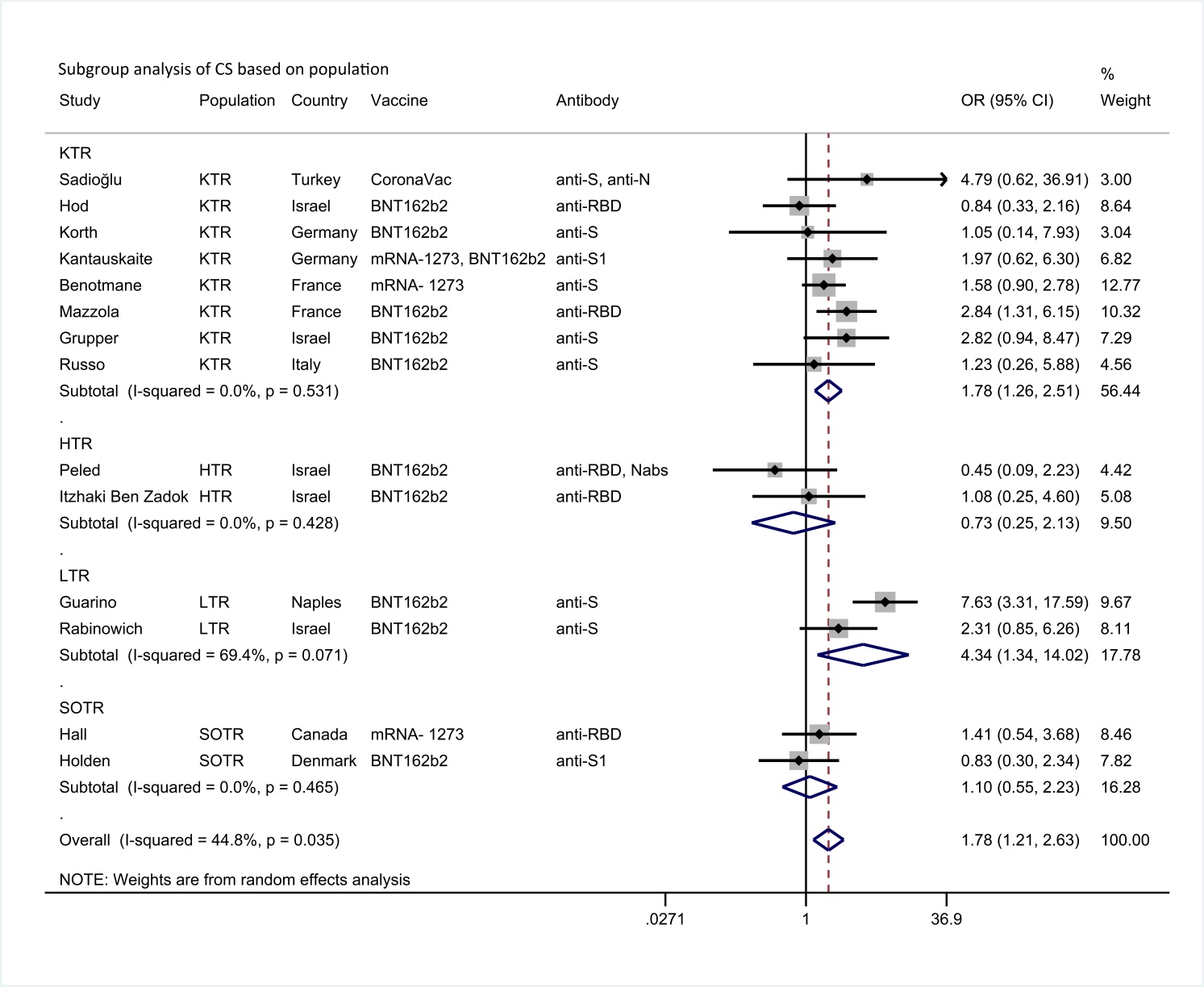

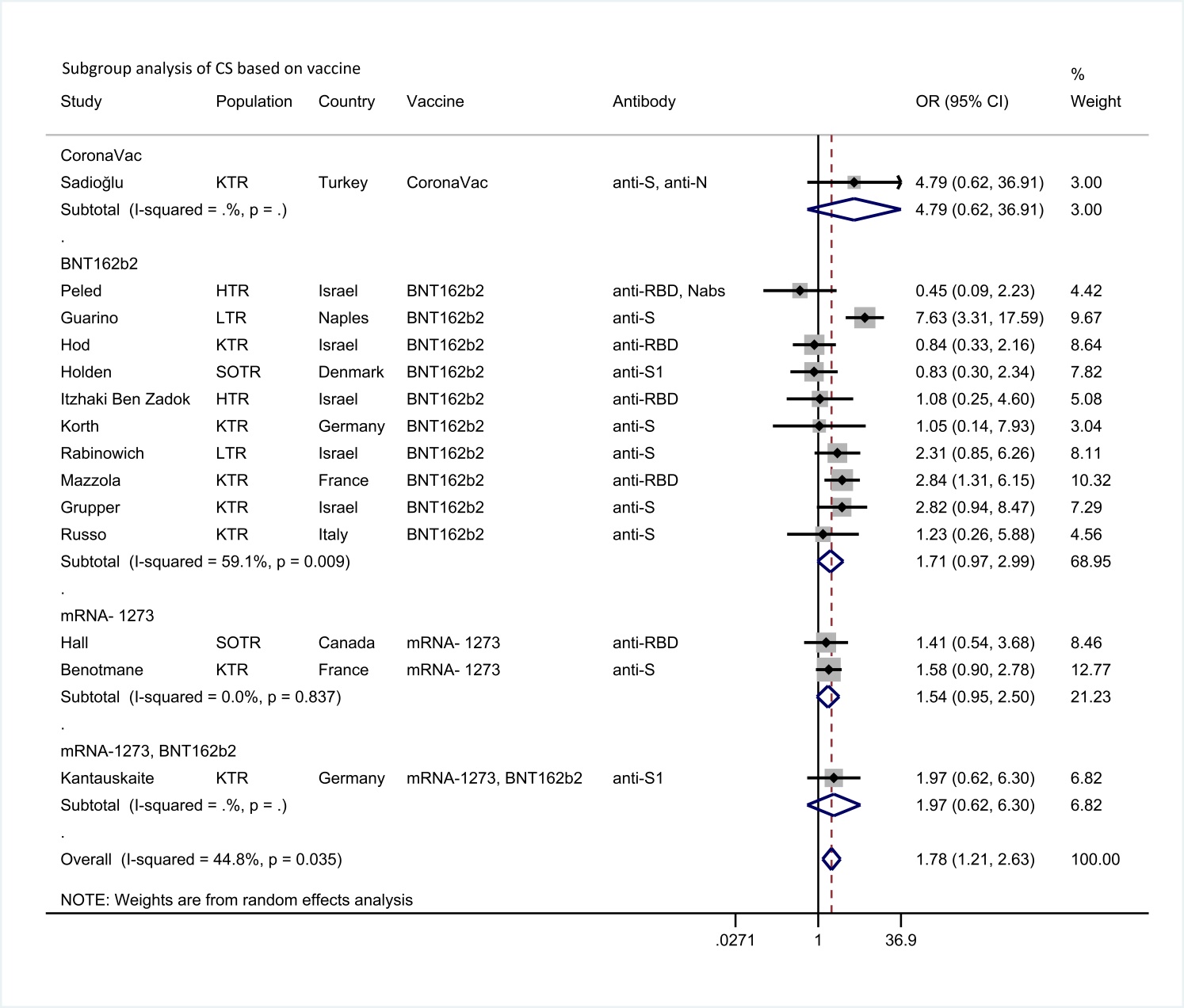


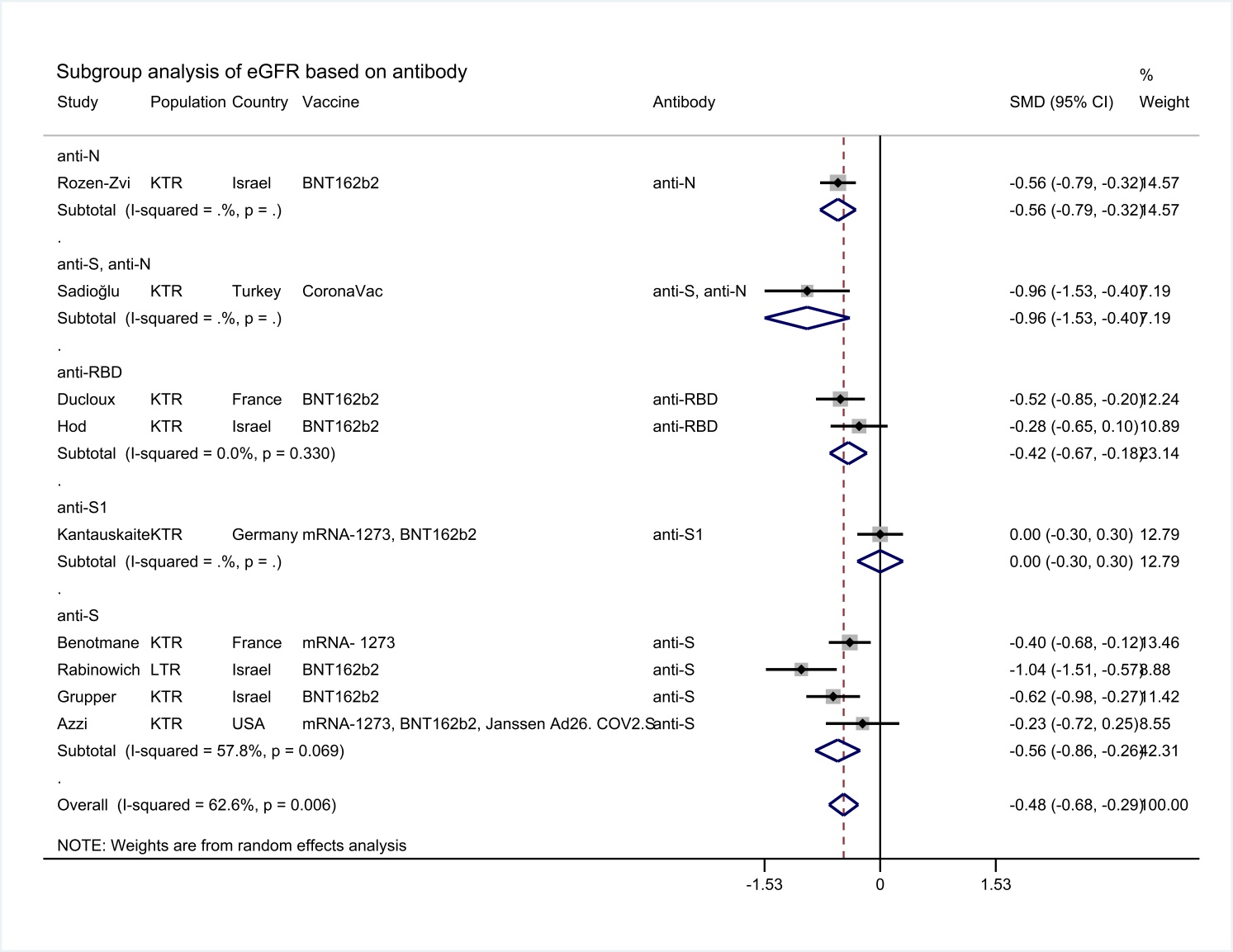

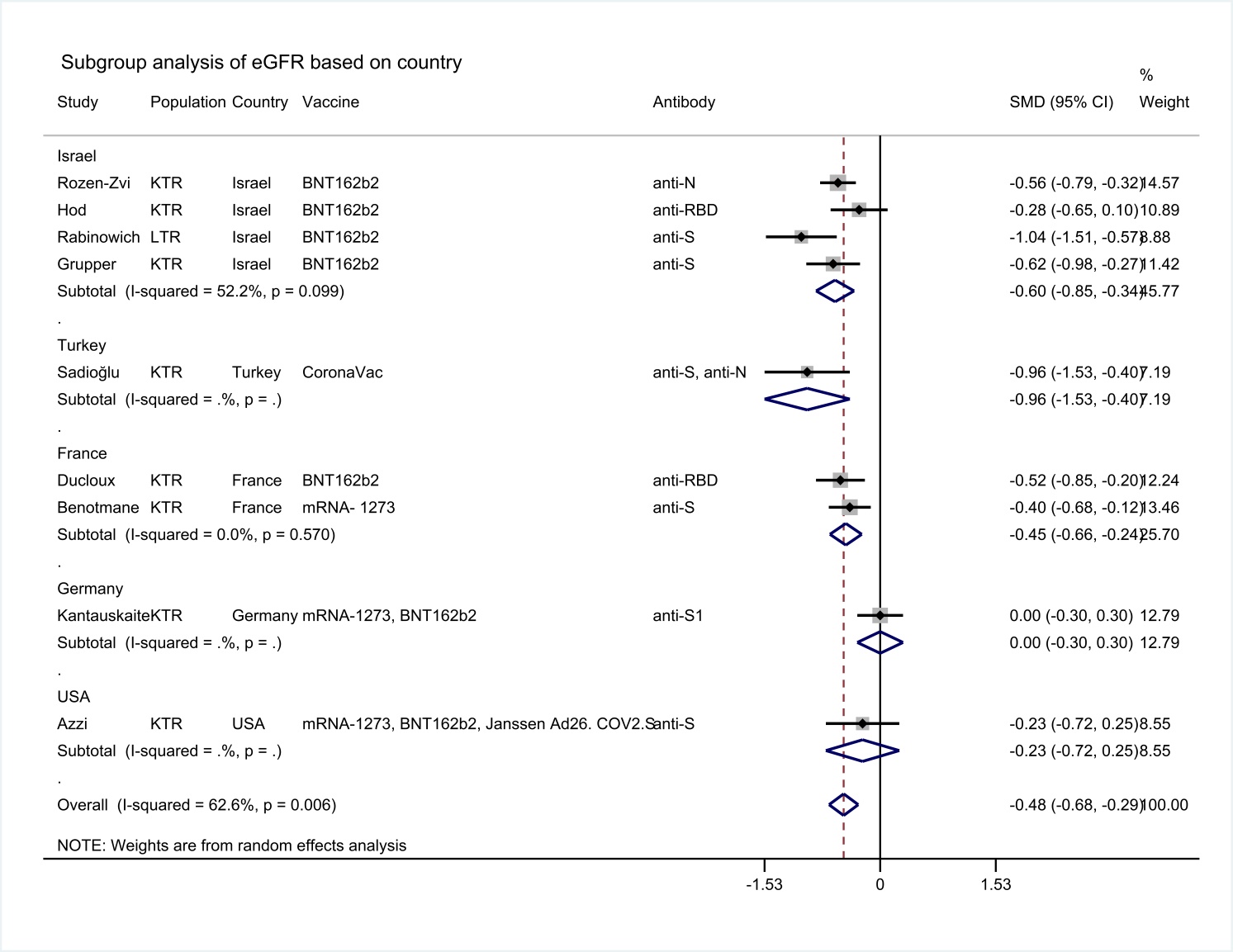

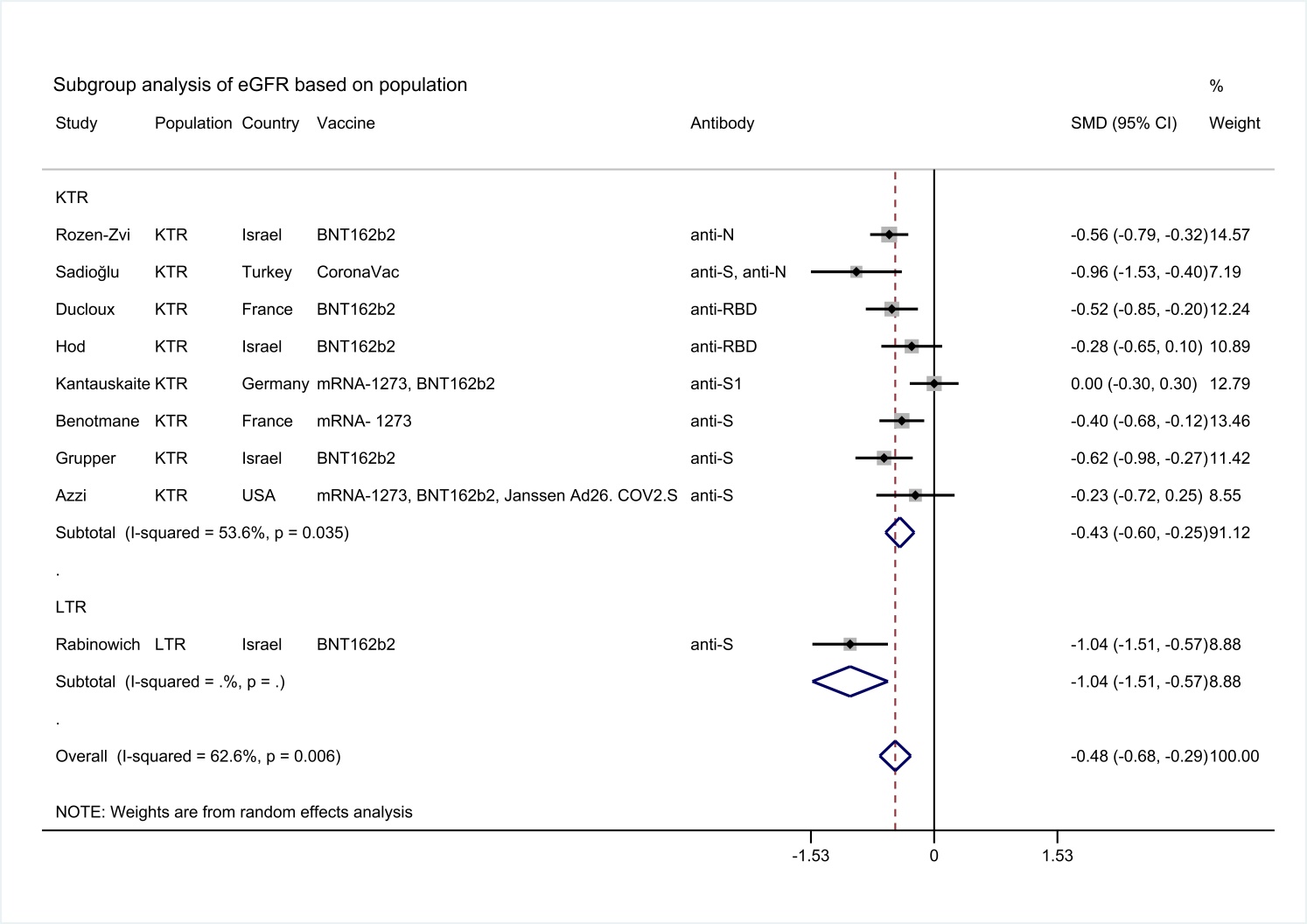

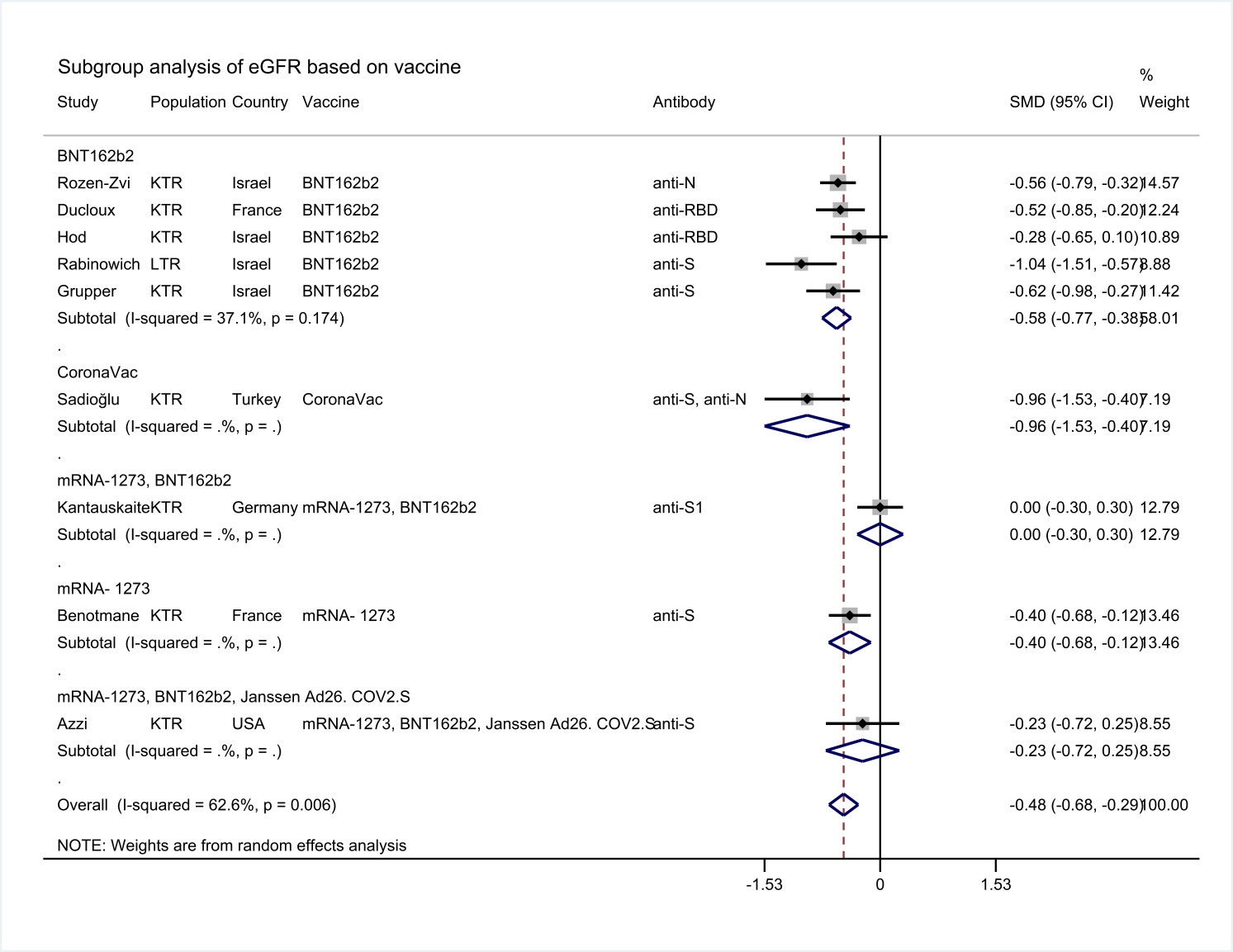


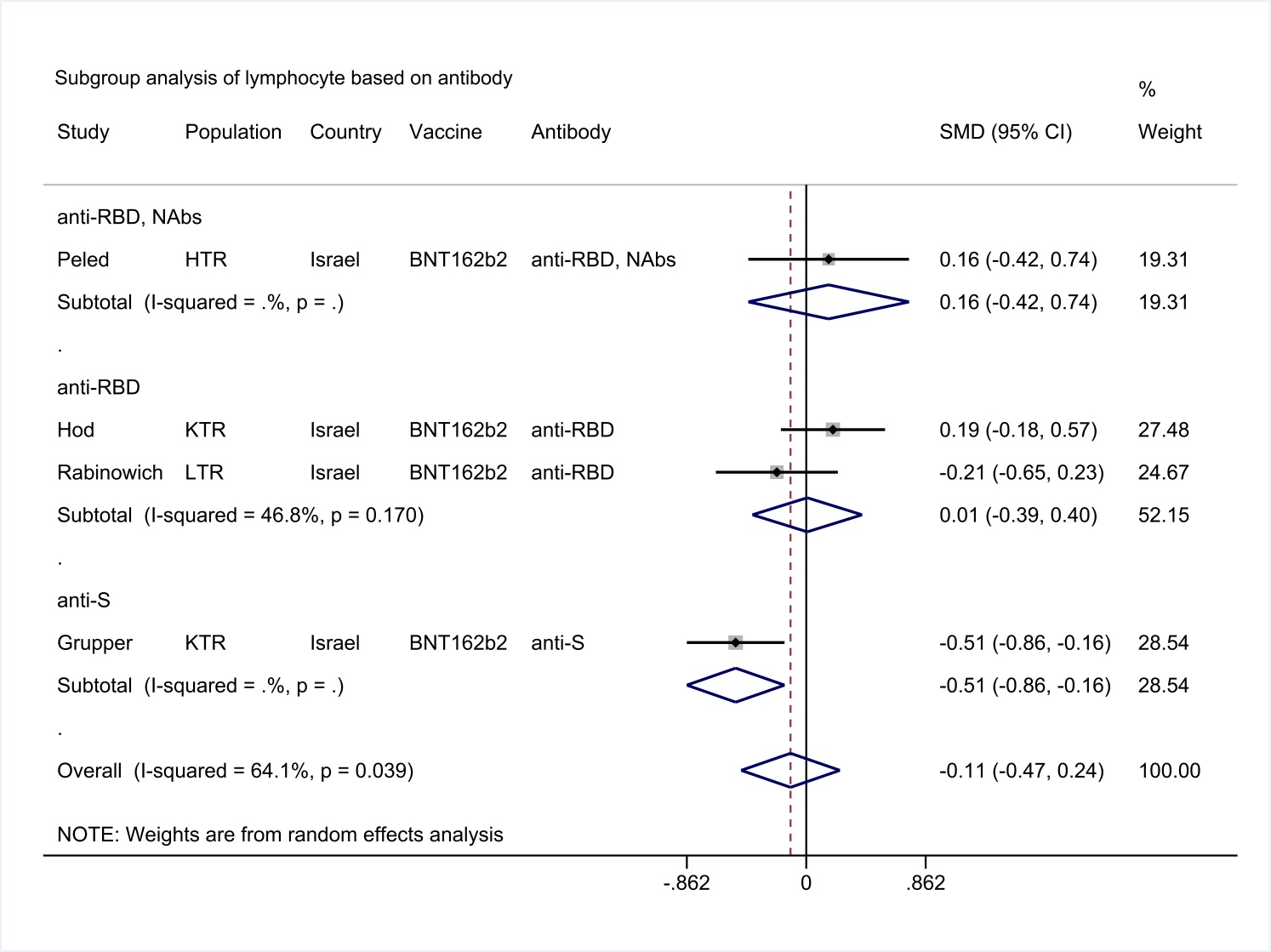

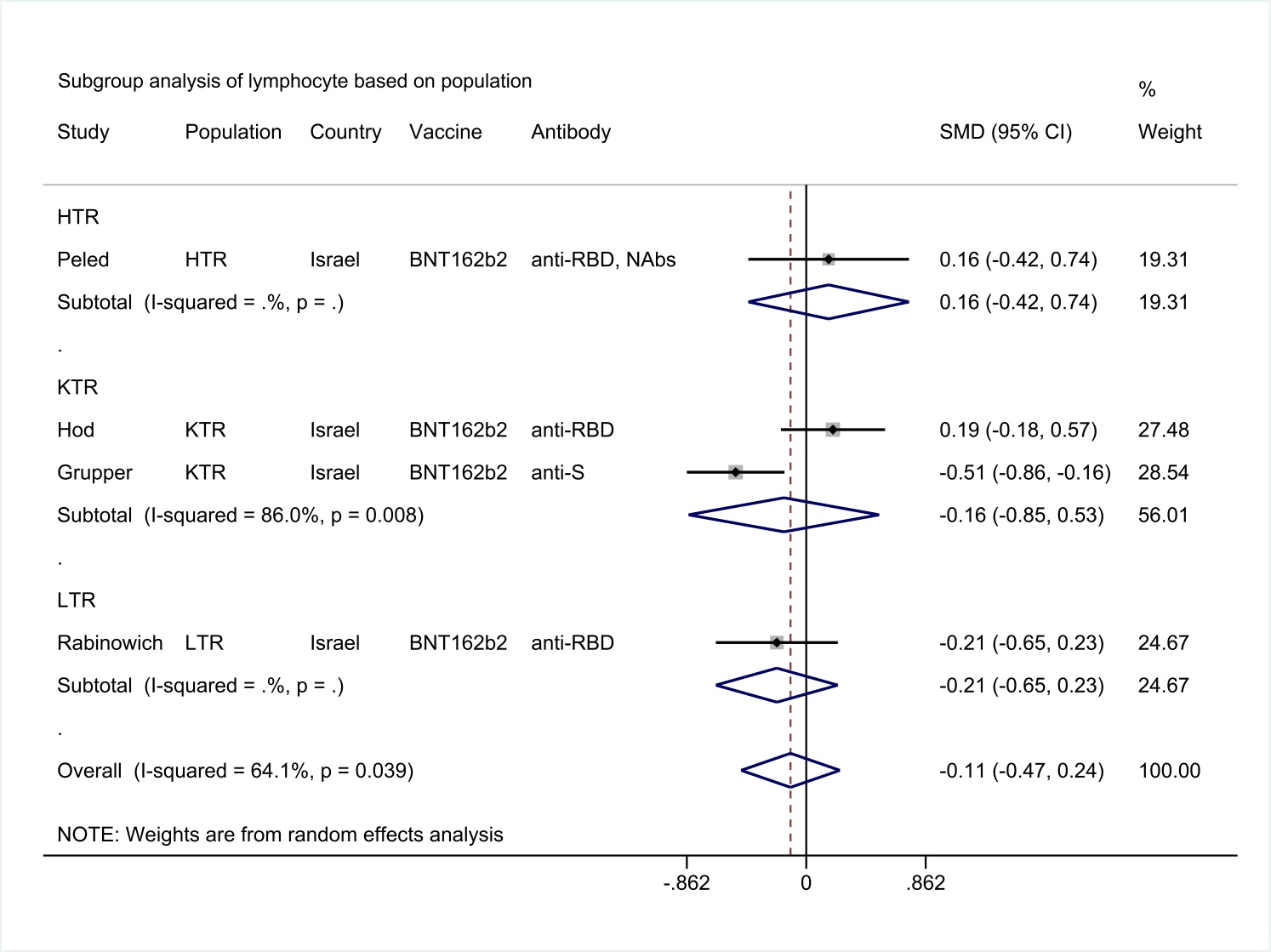


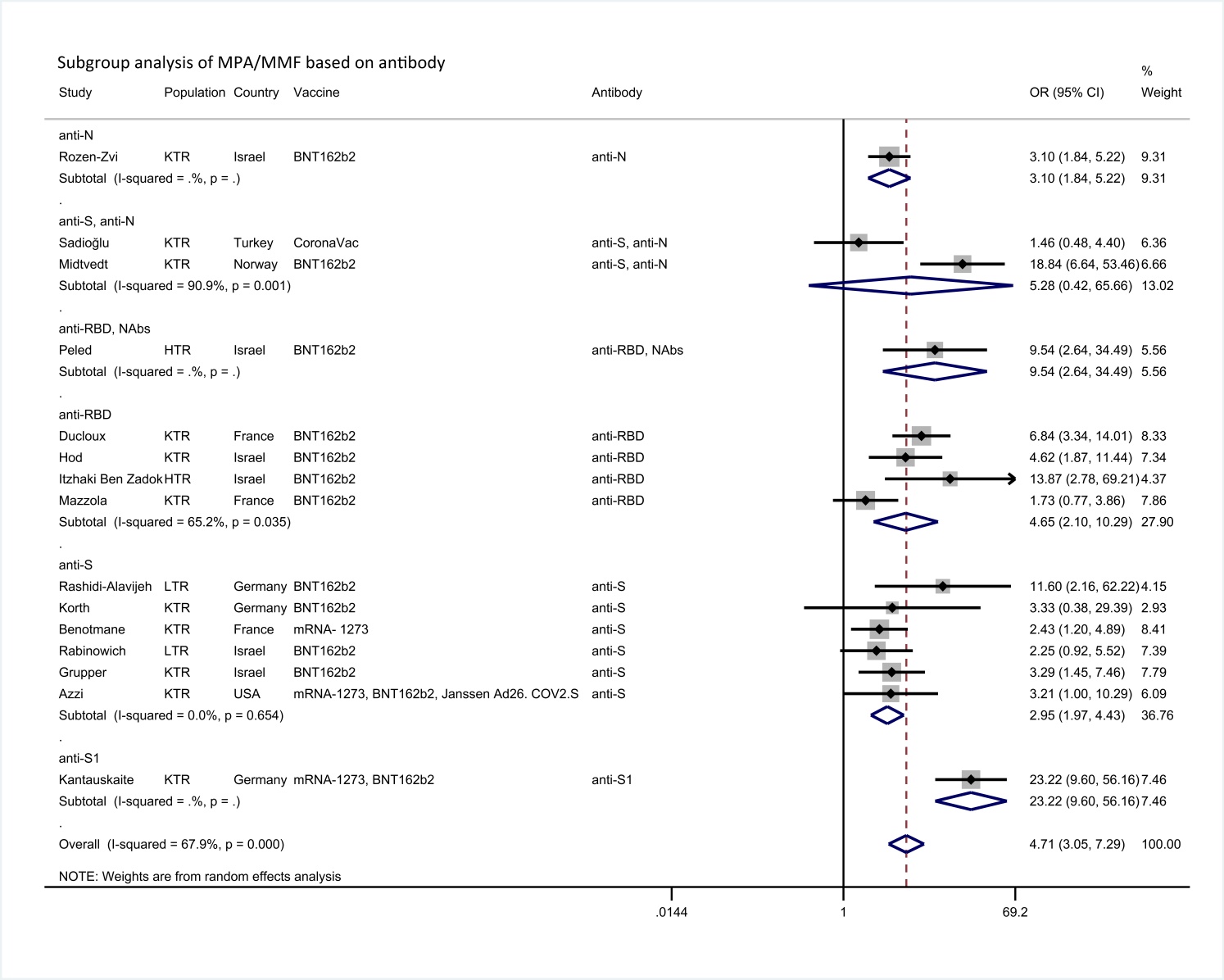

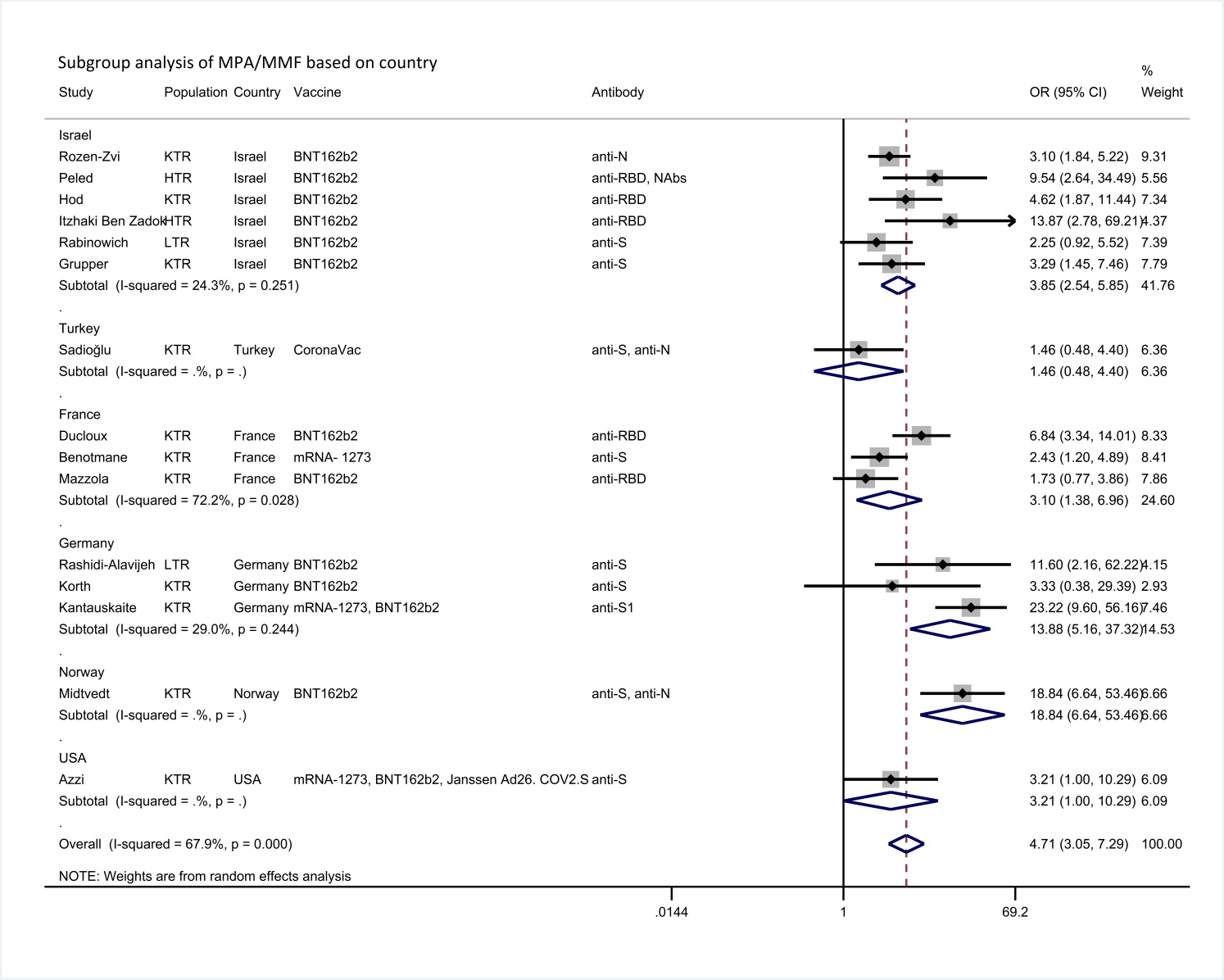

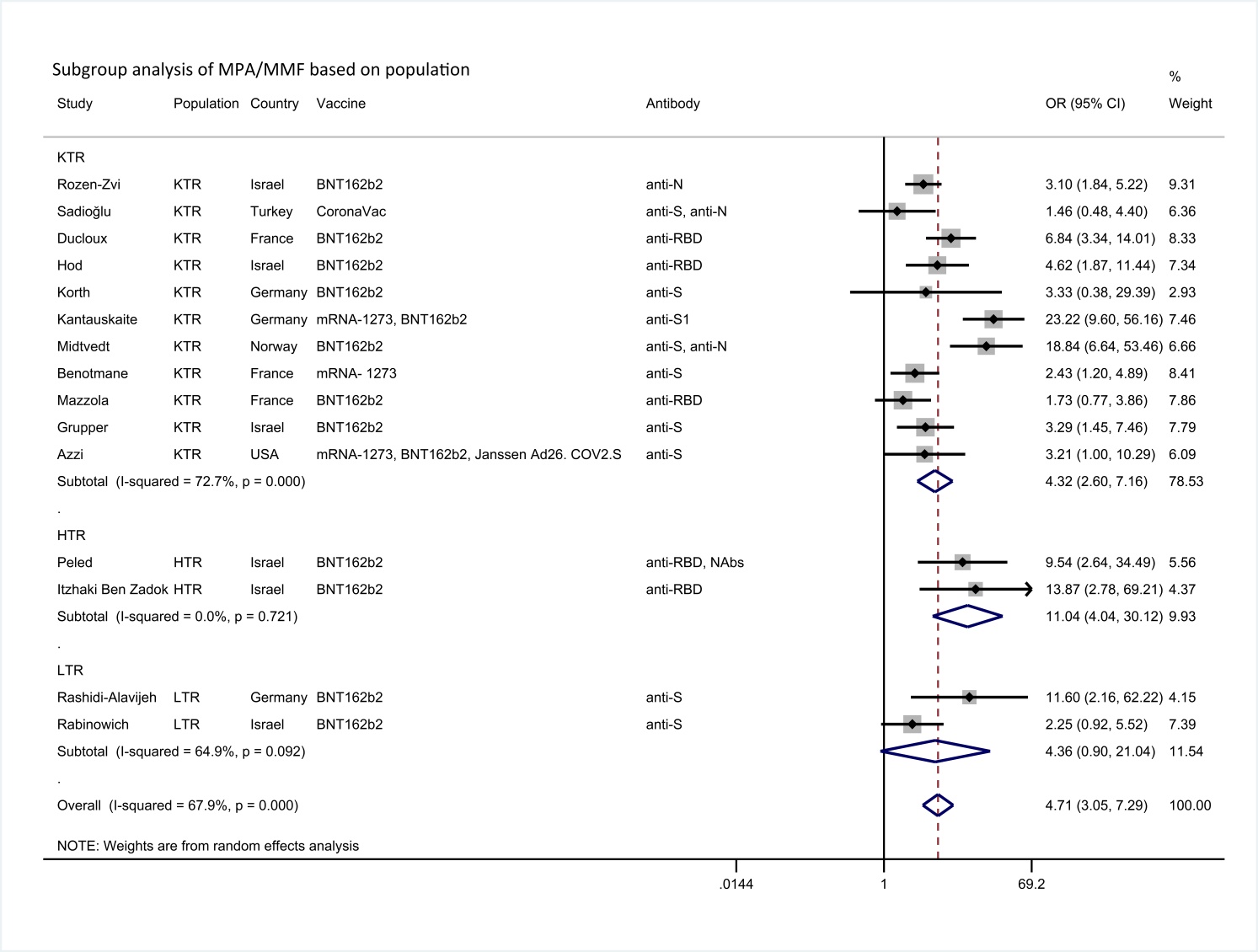

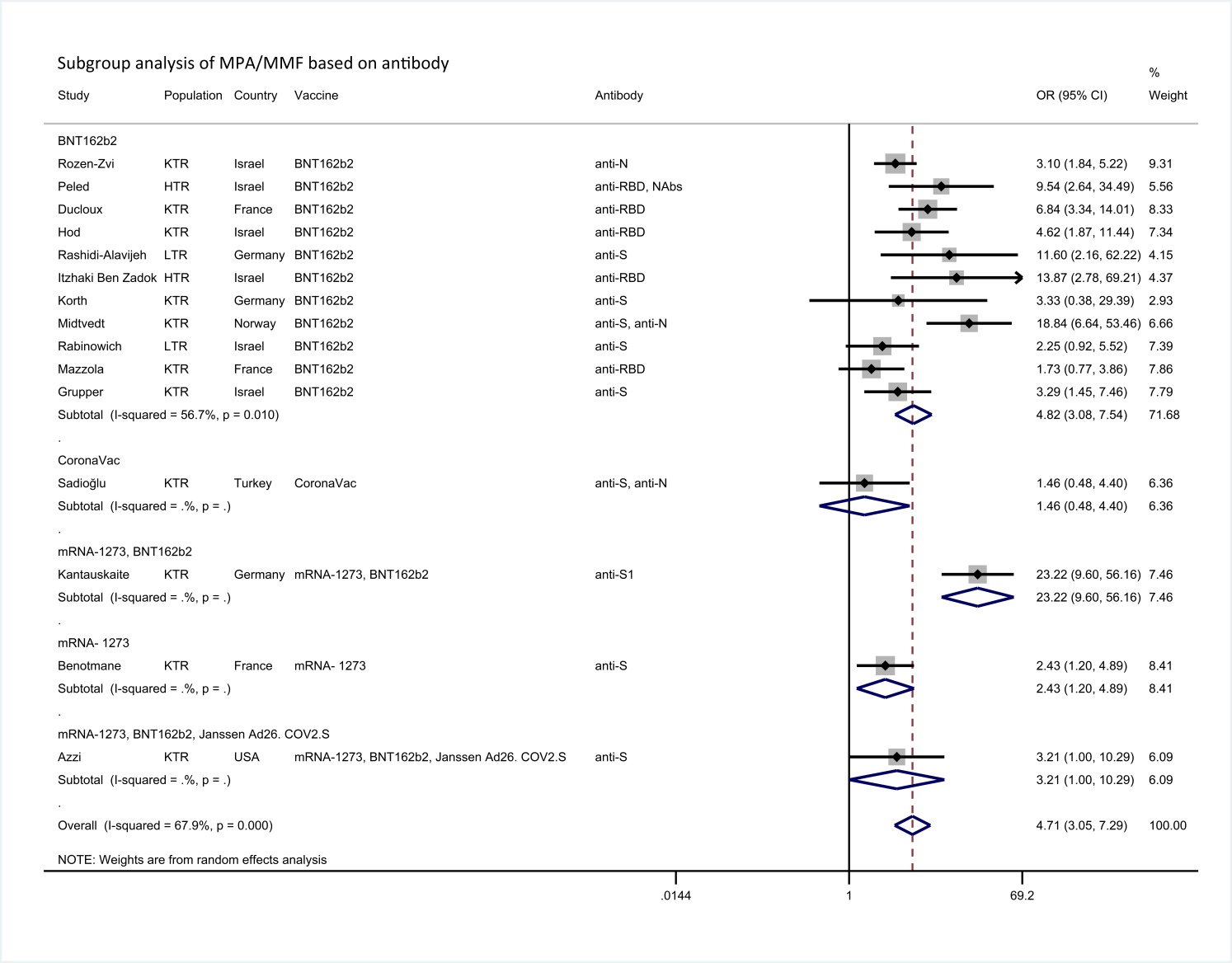


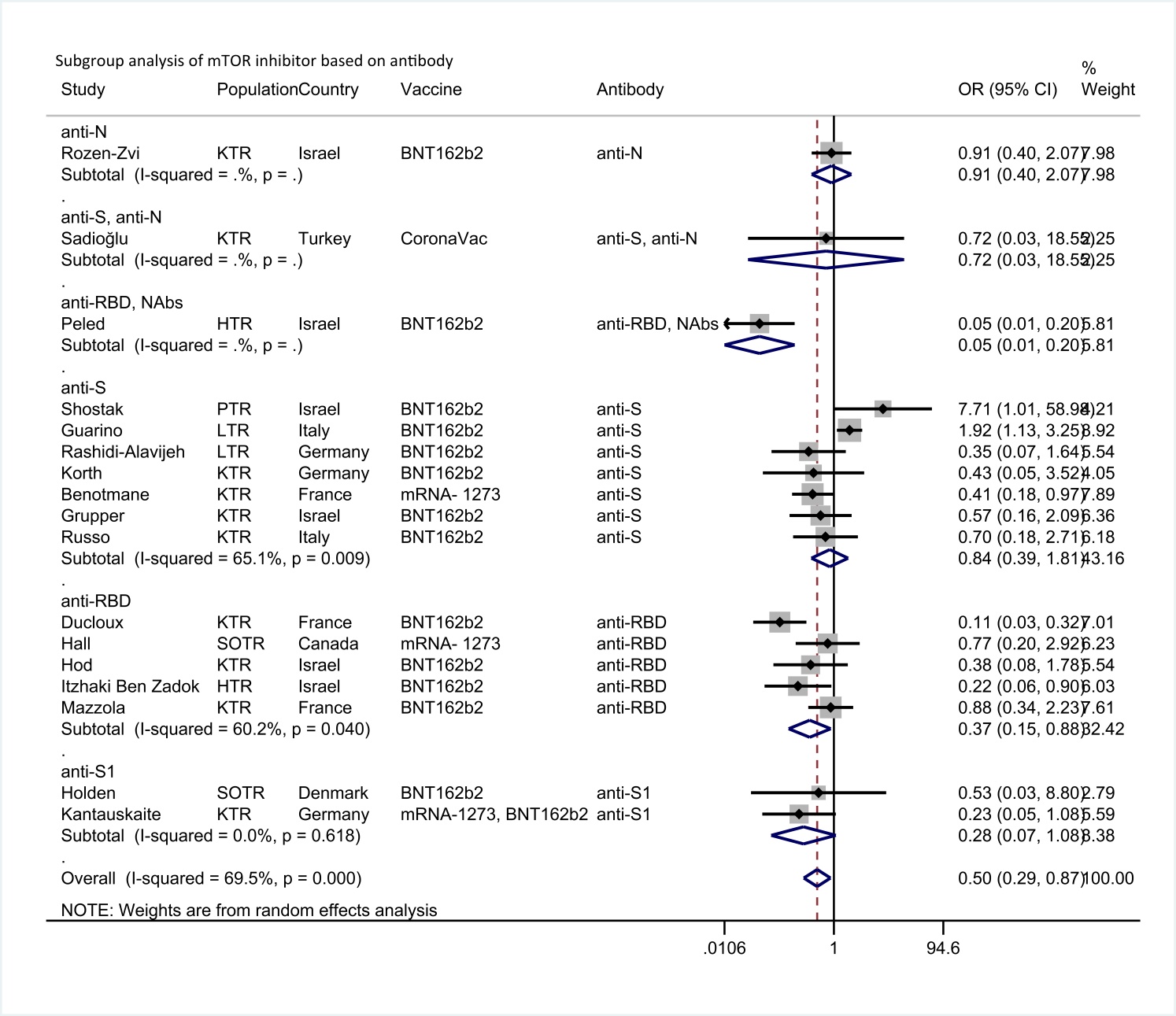

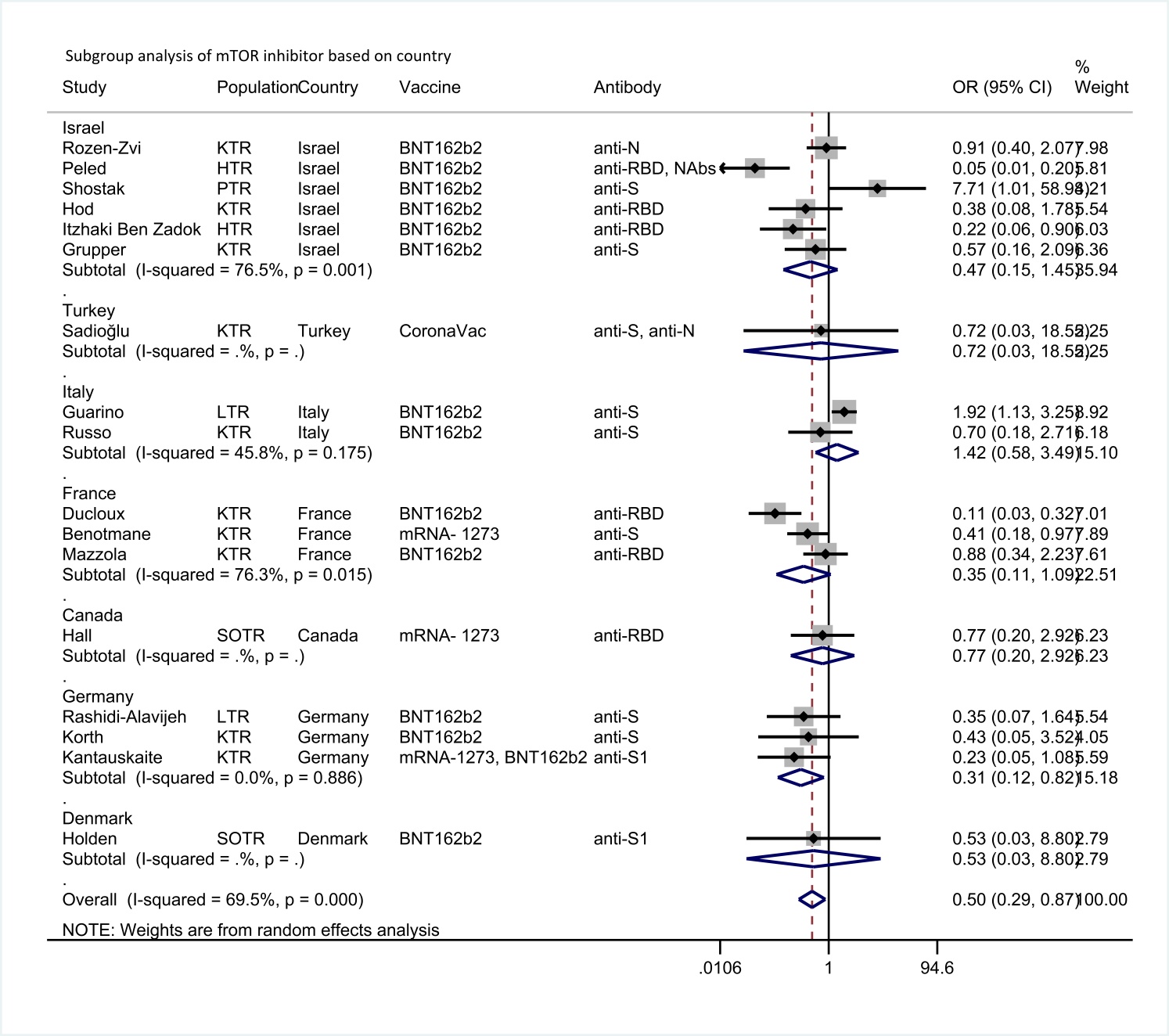

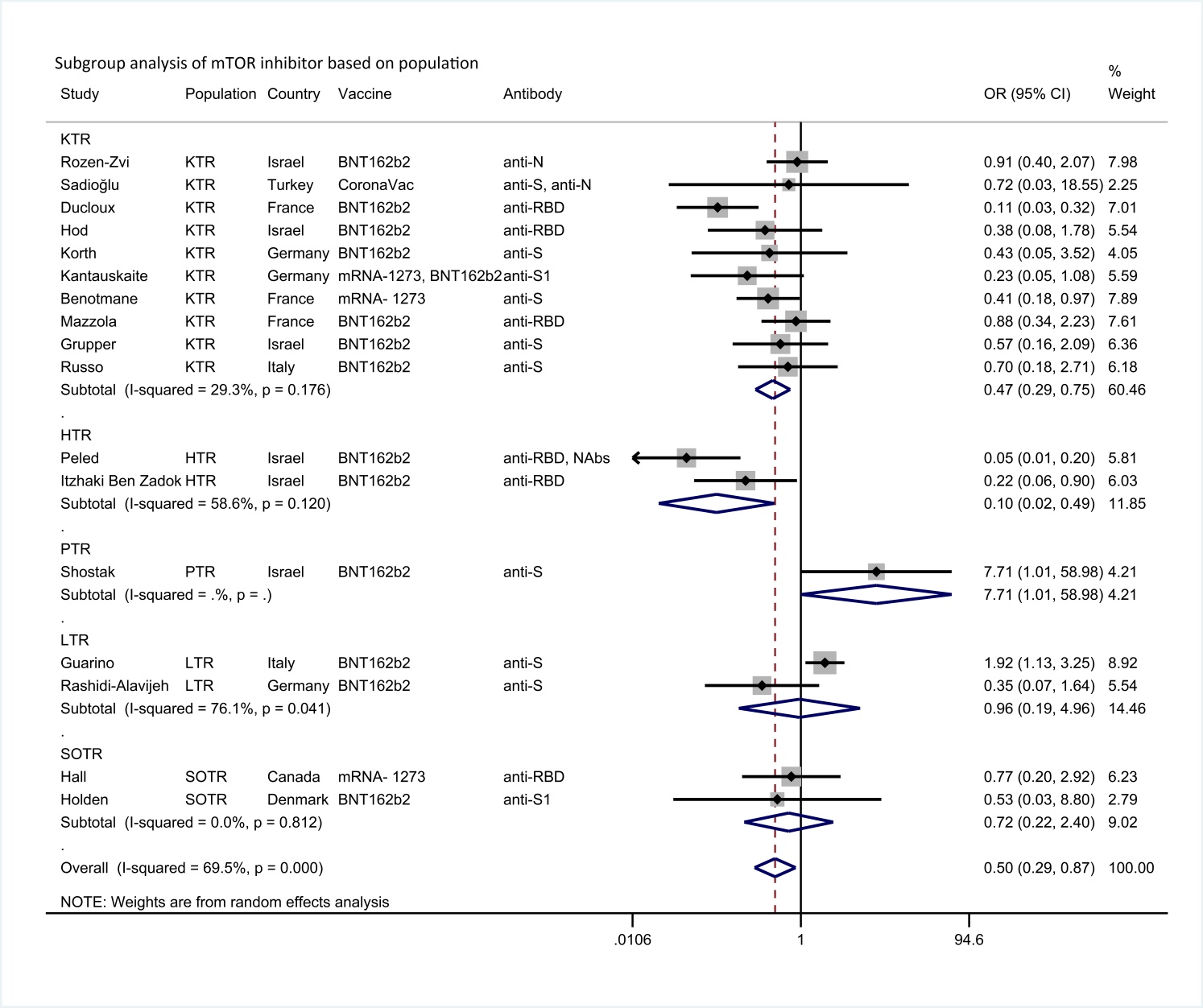

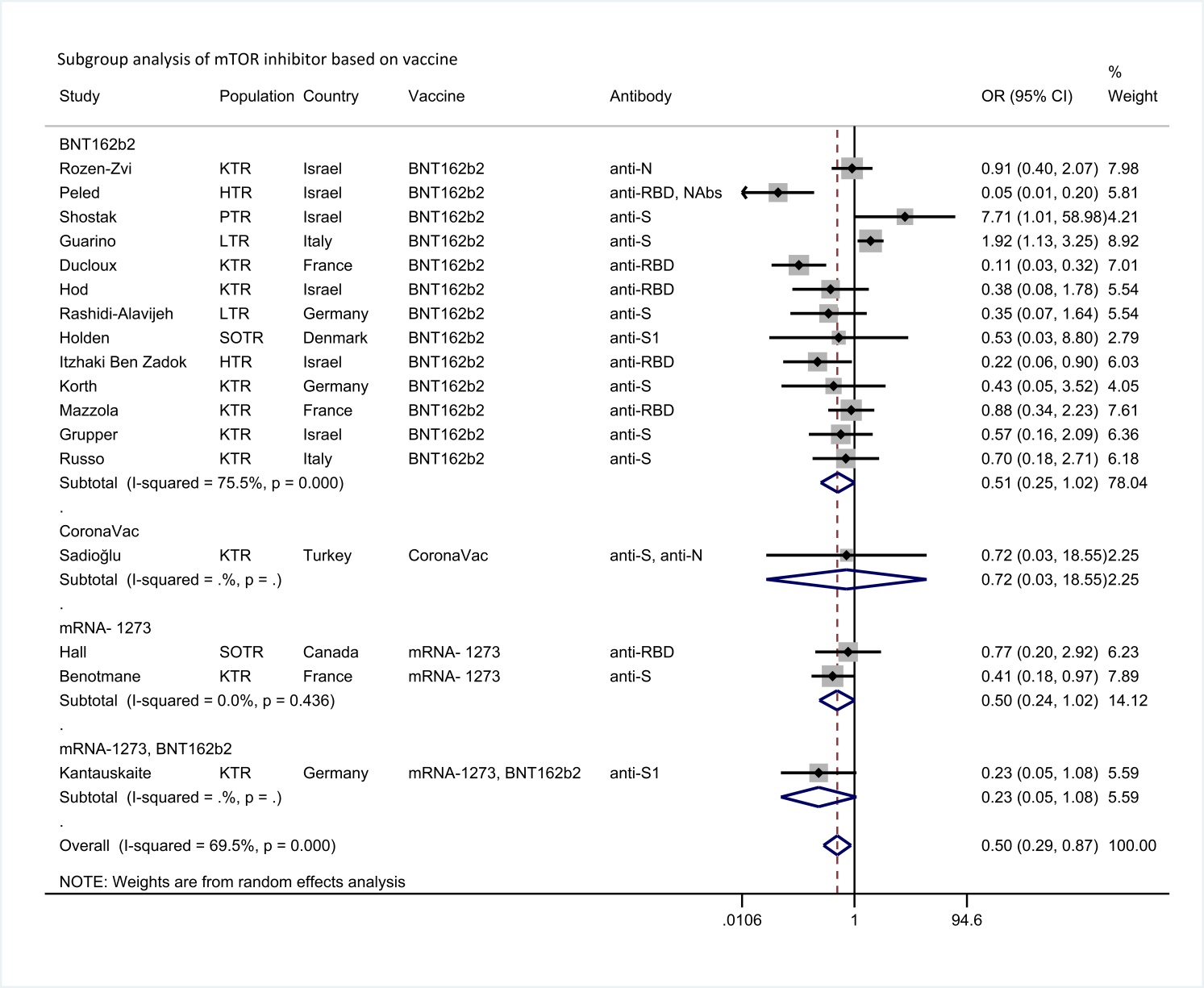


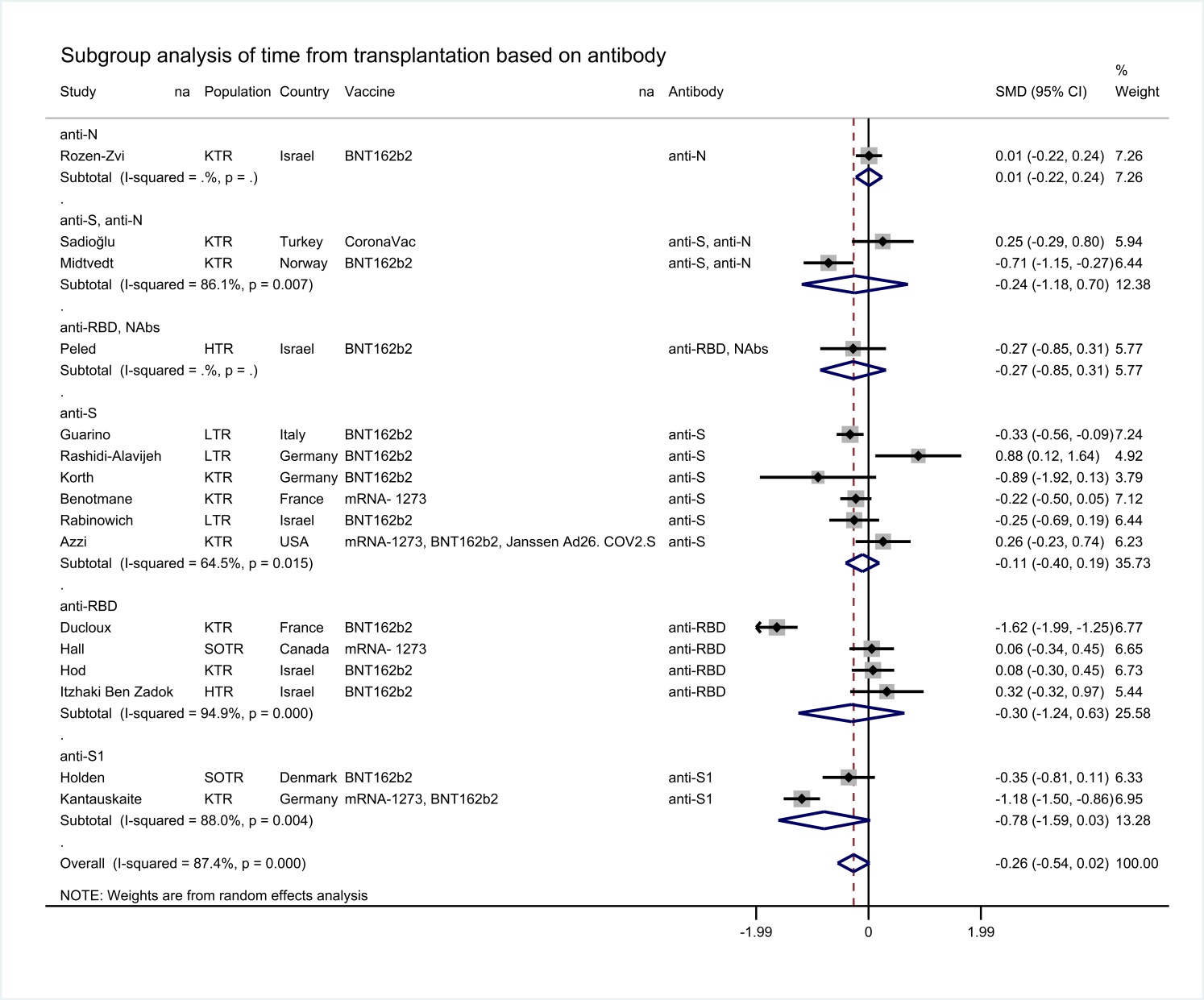

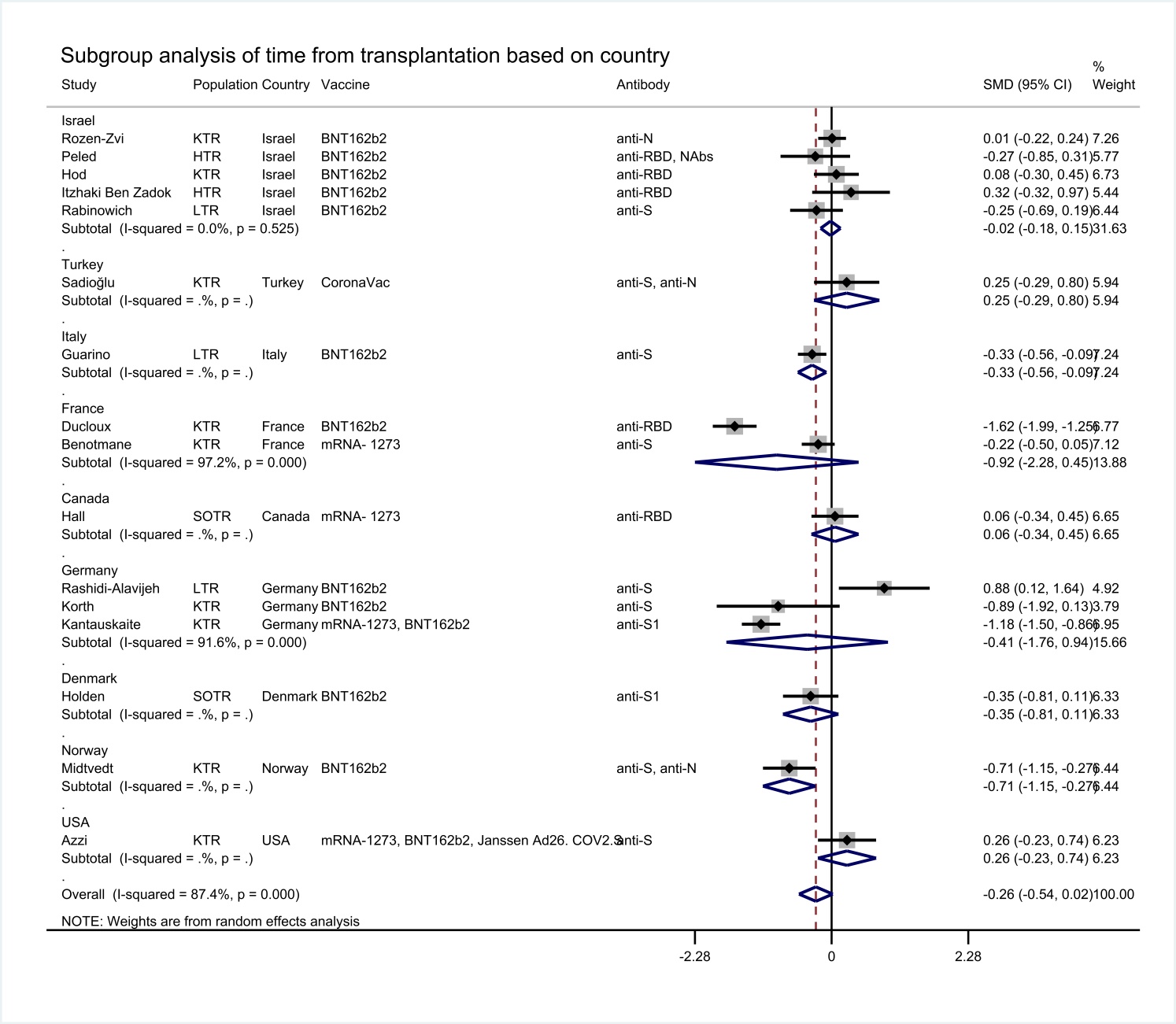

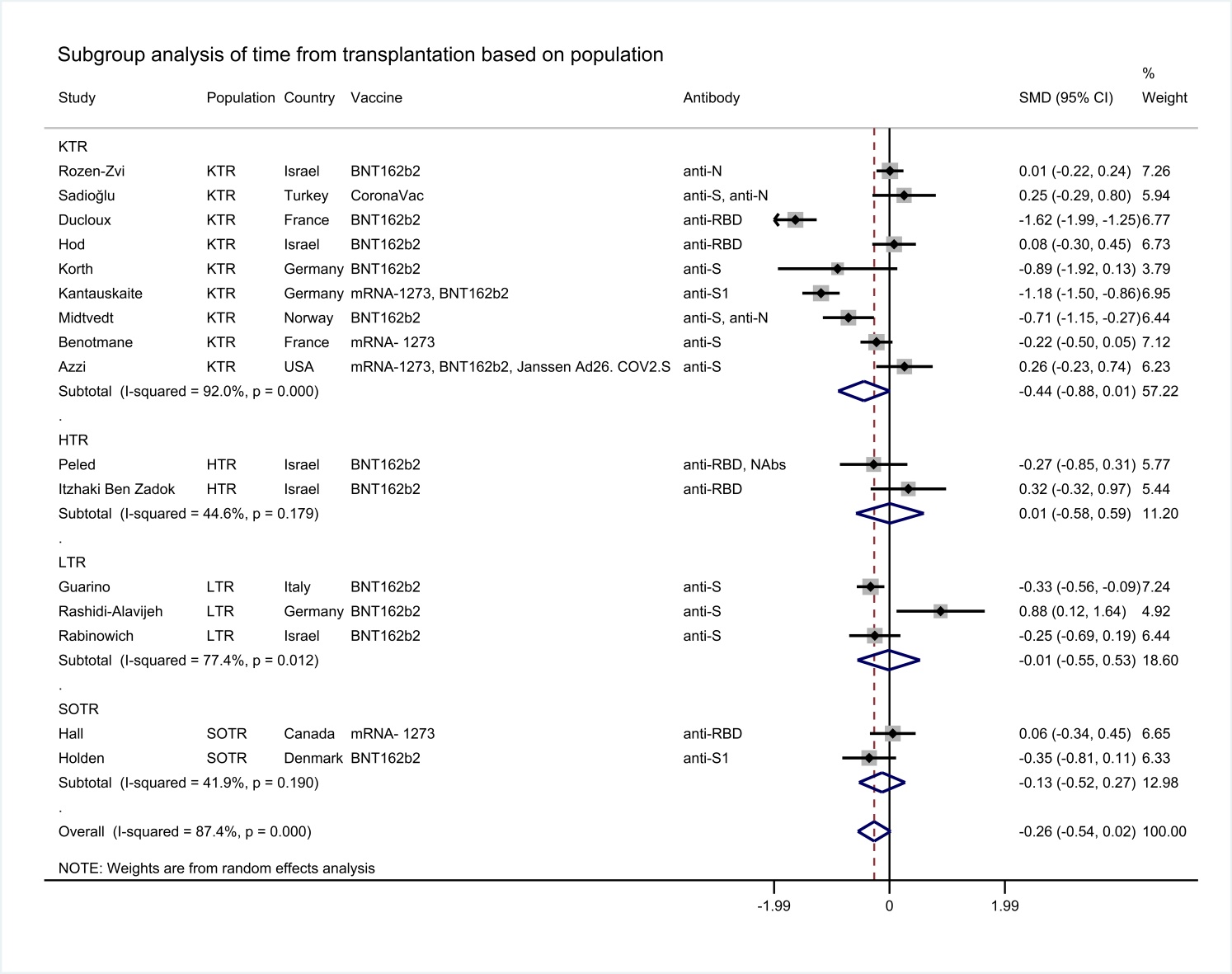

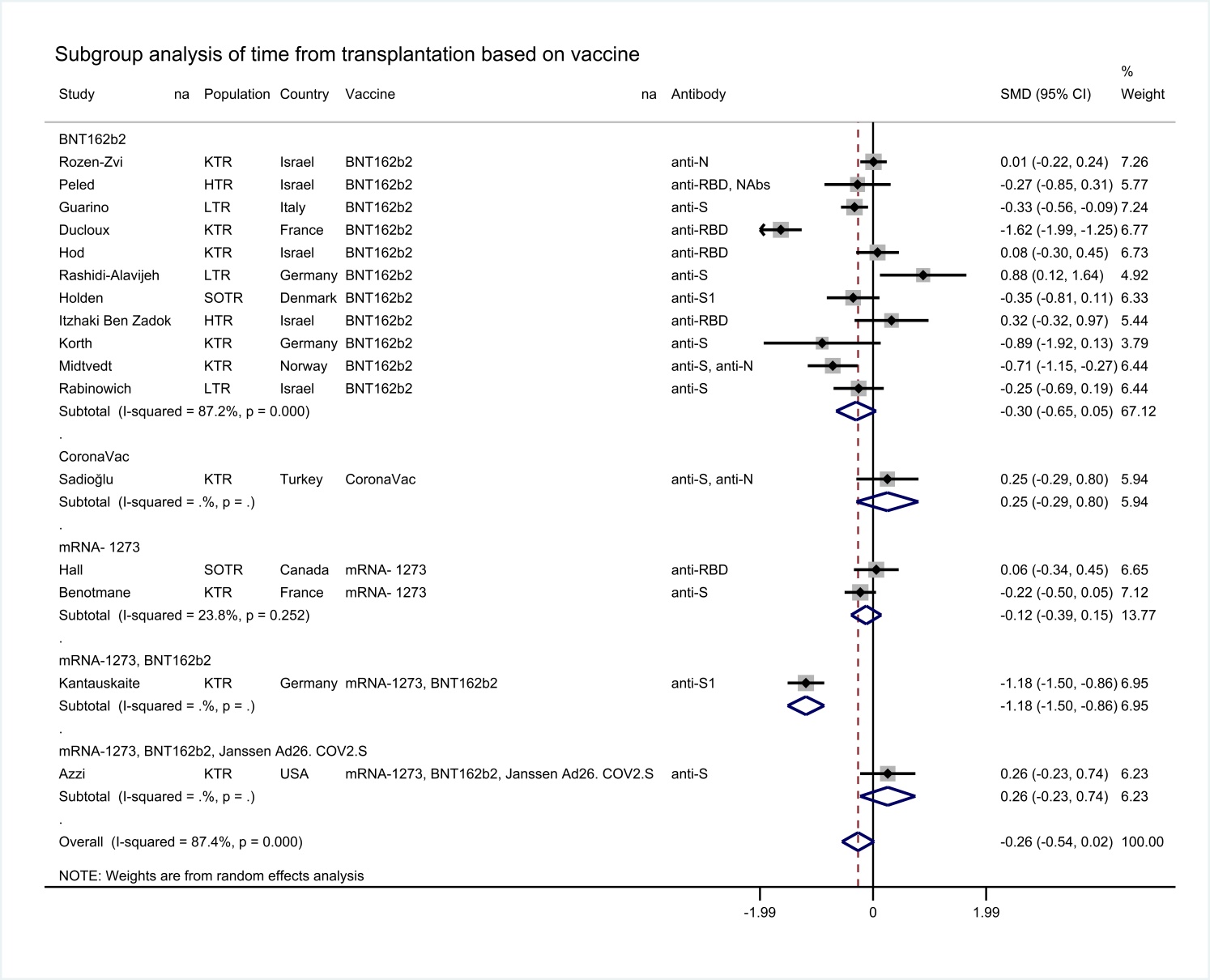


Galbraith plot


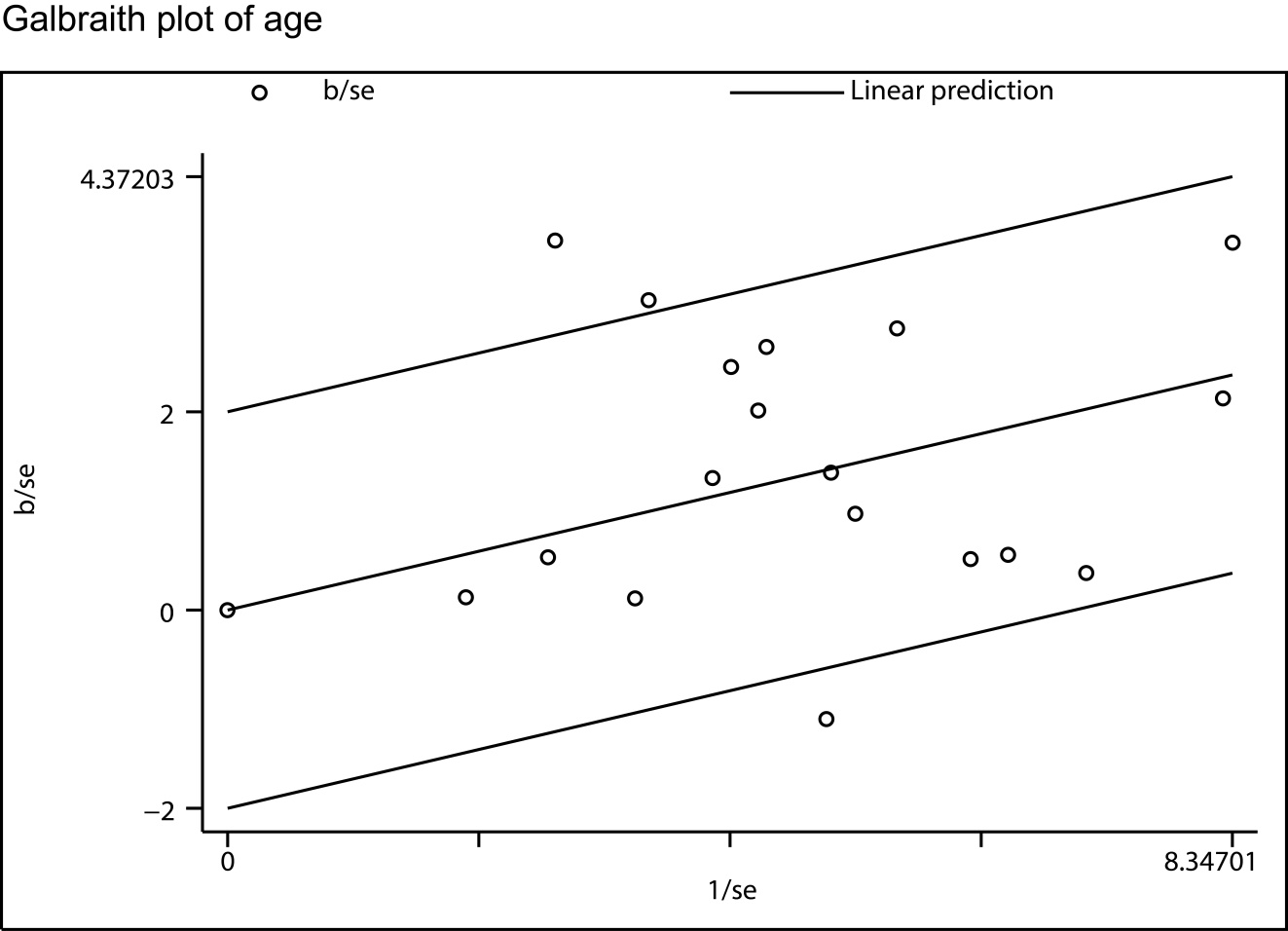

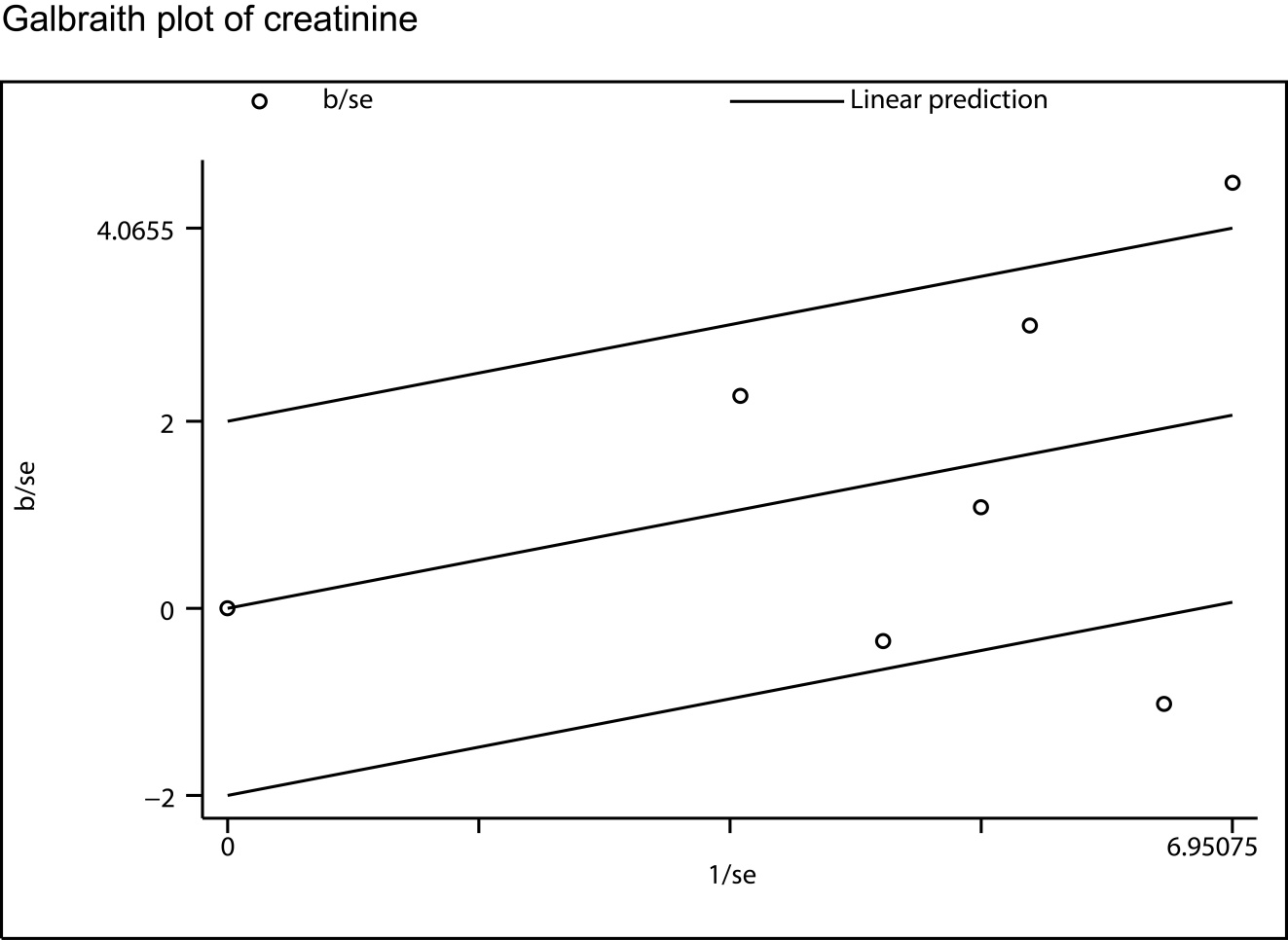

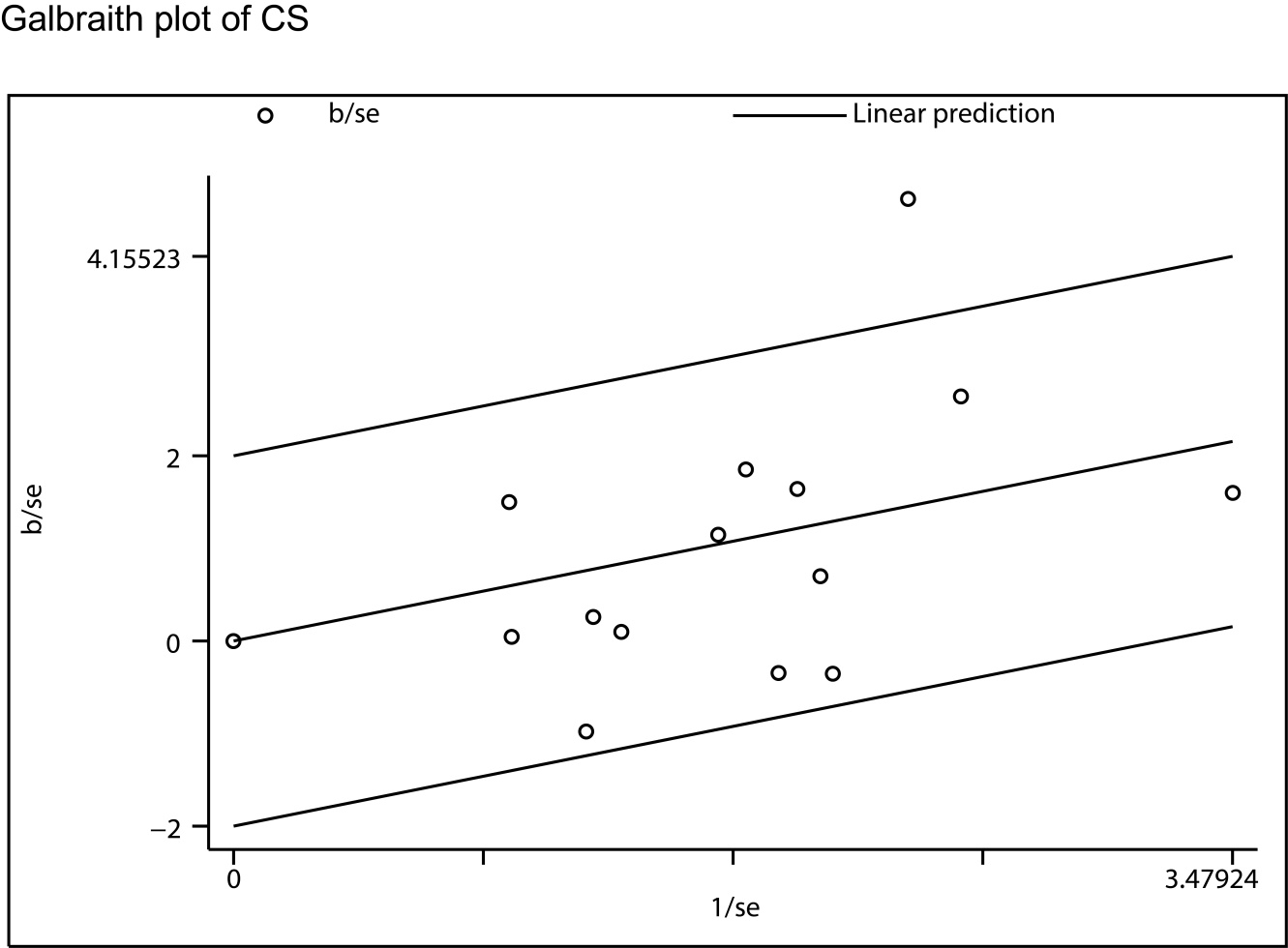

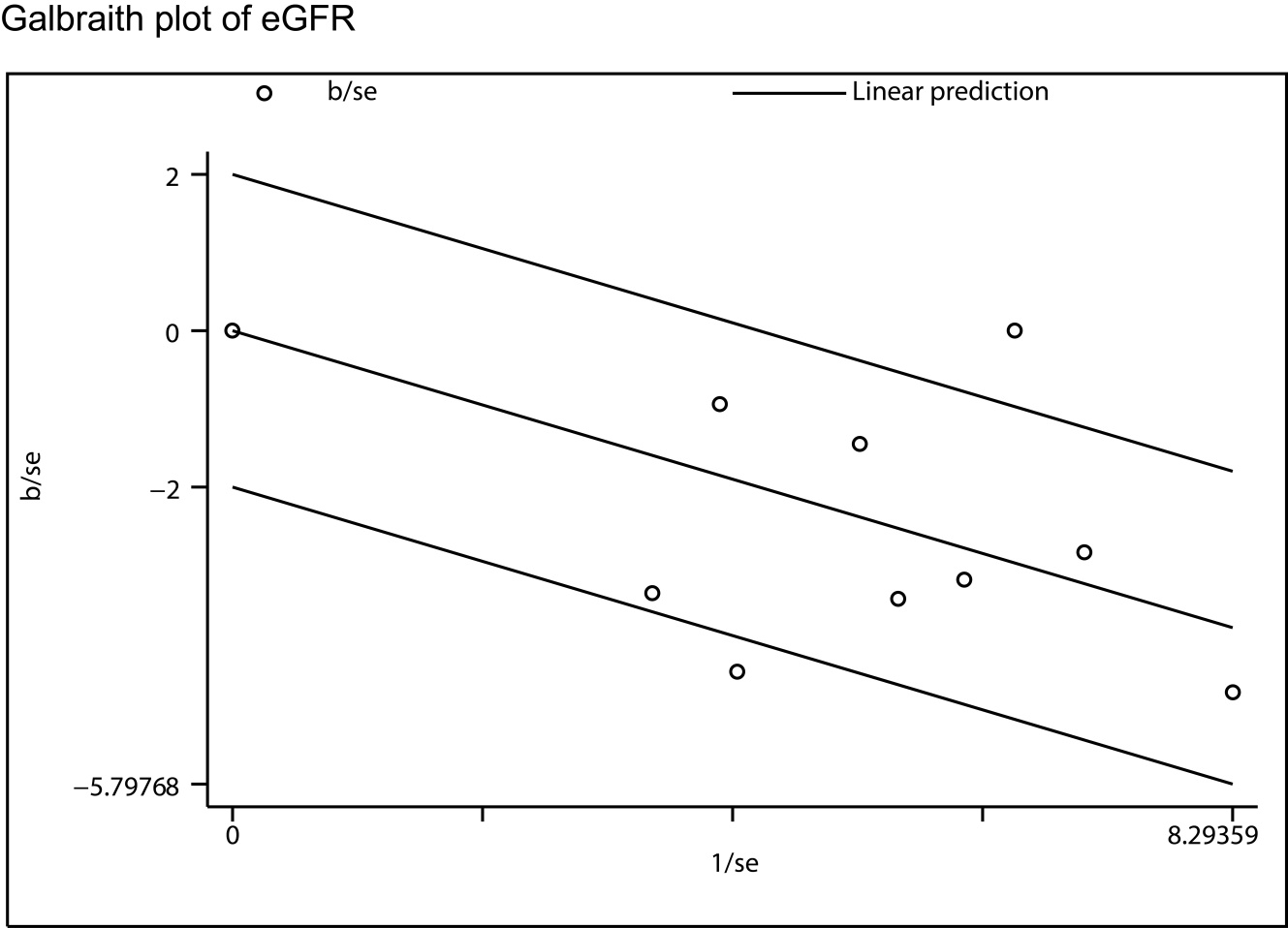

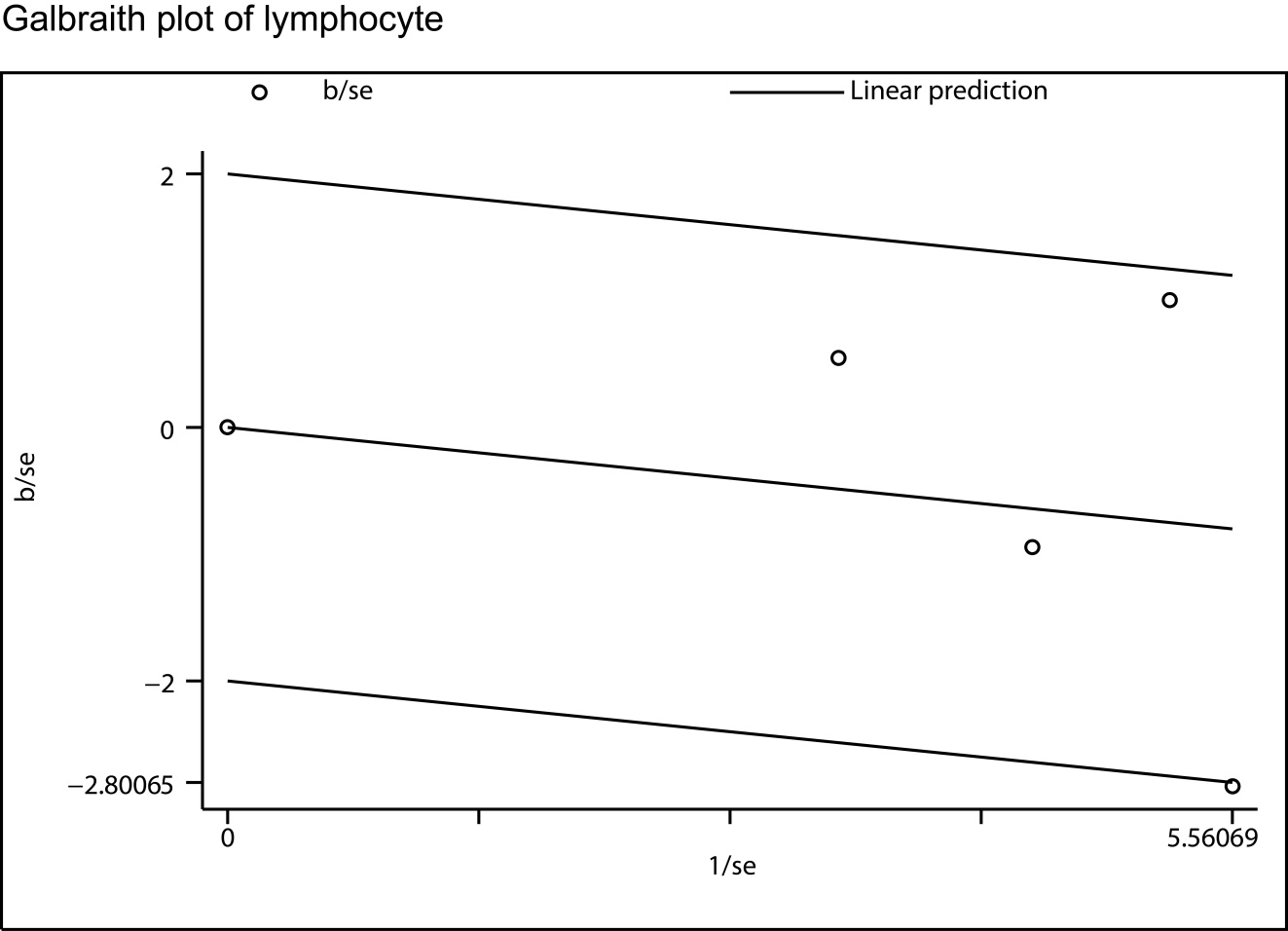

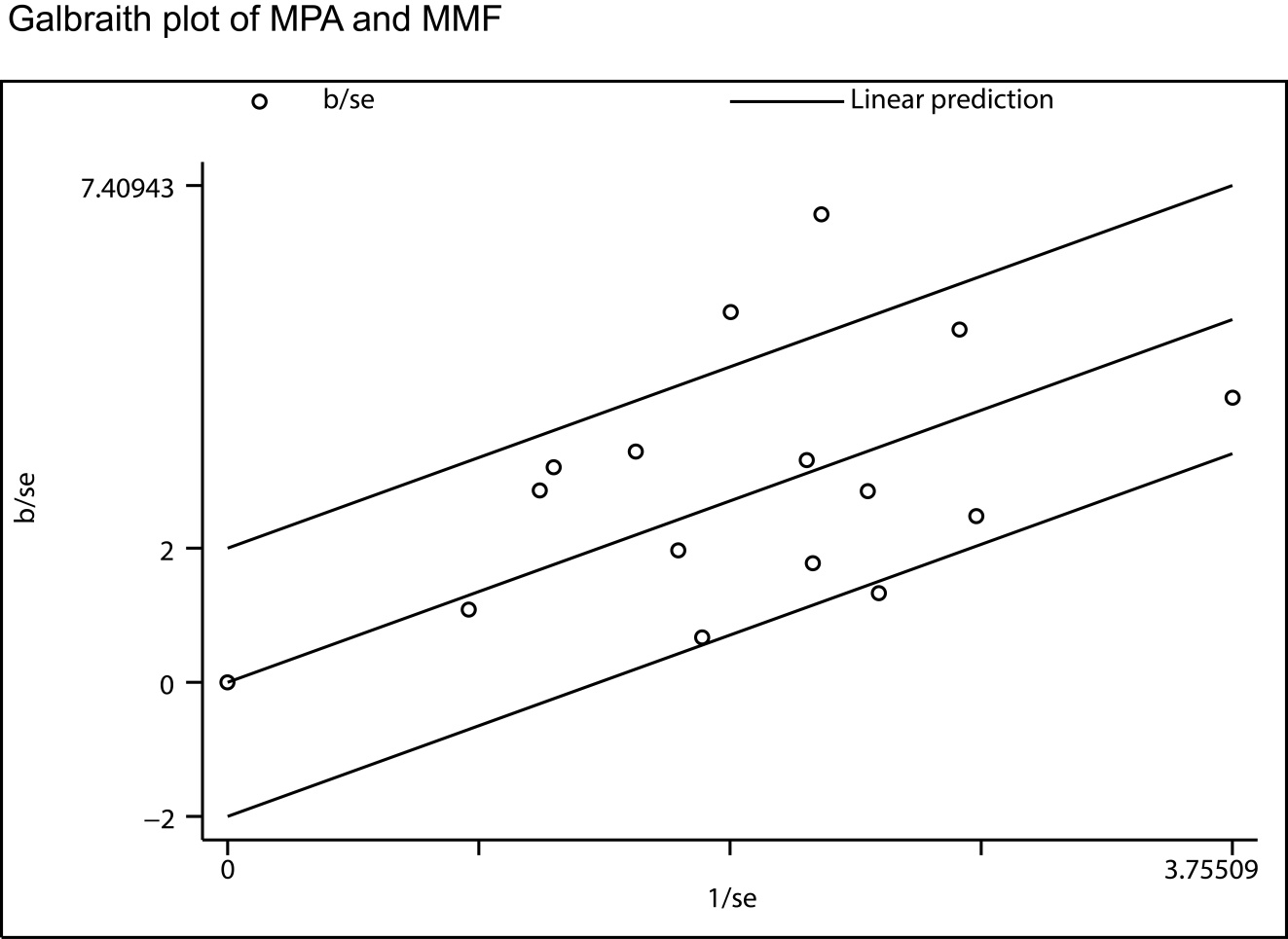

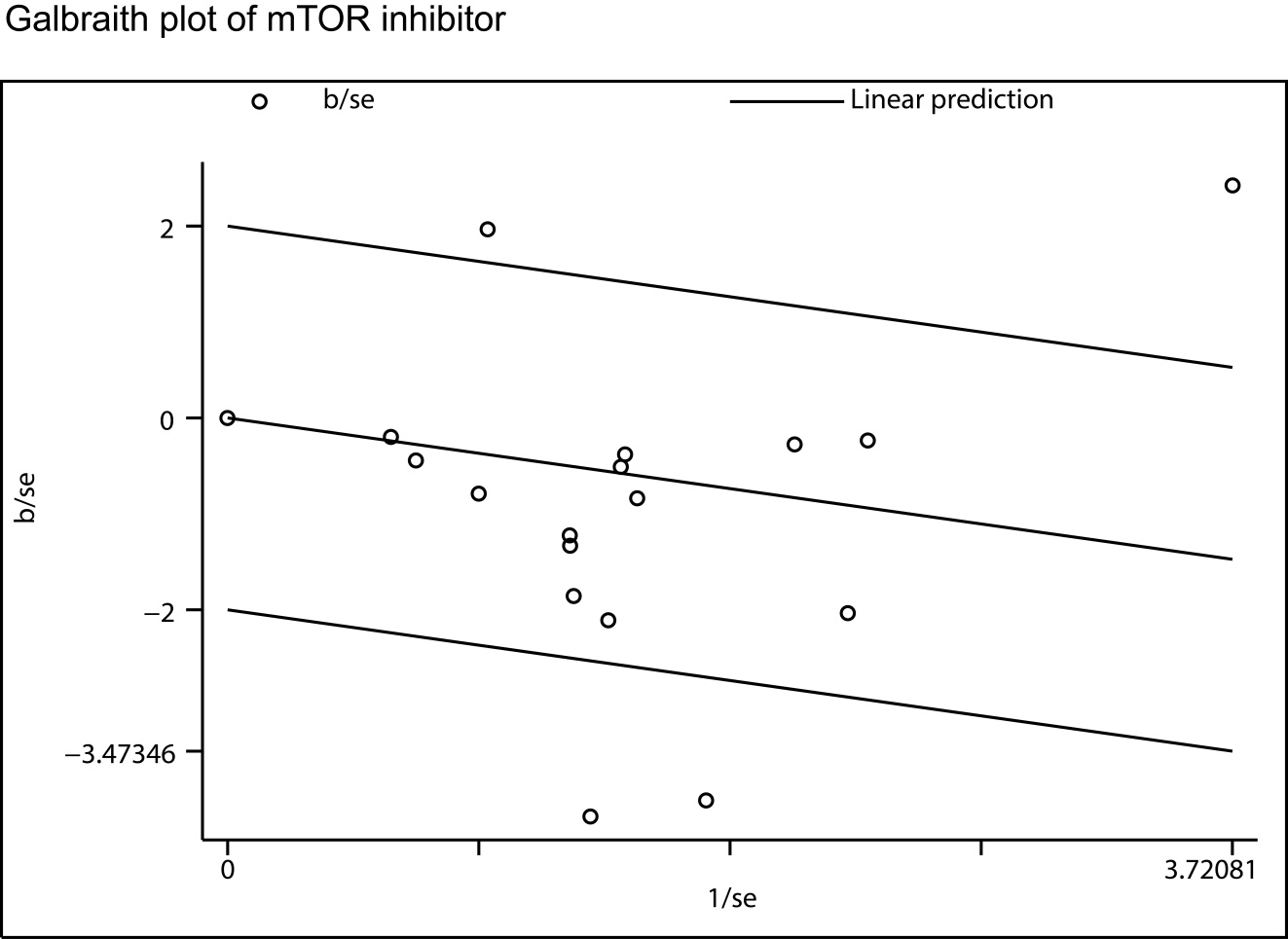

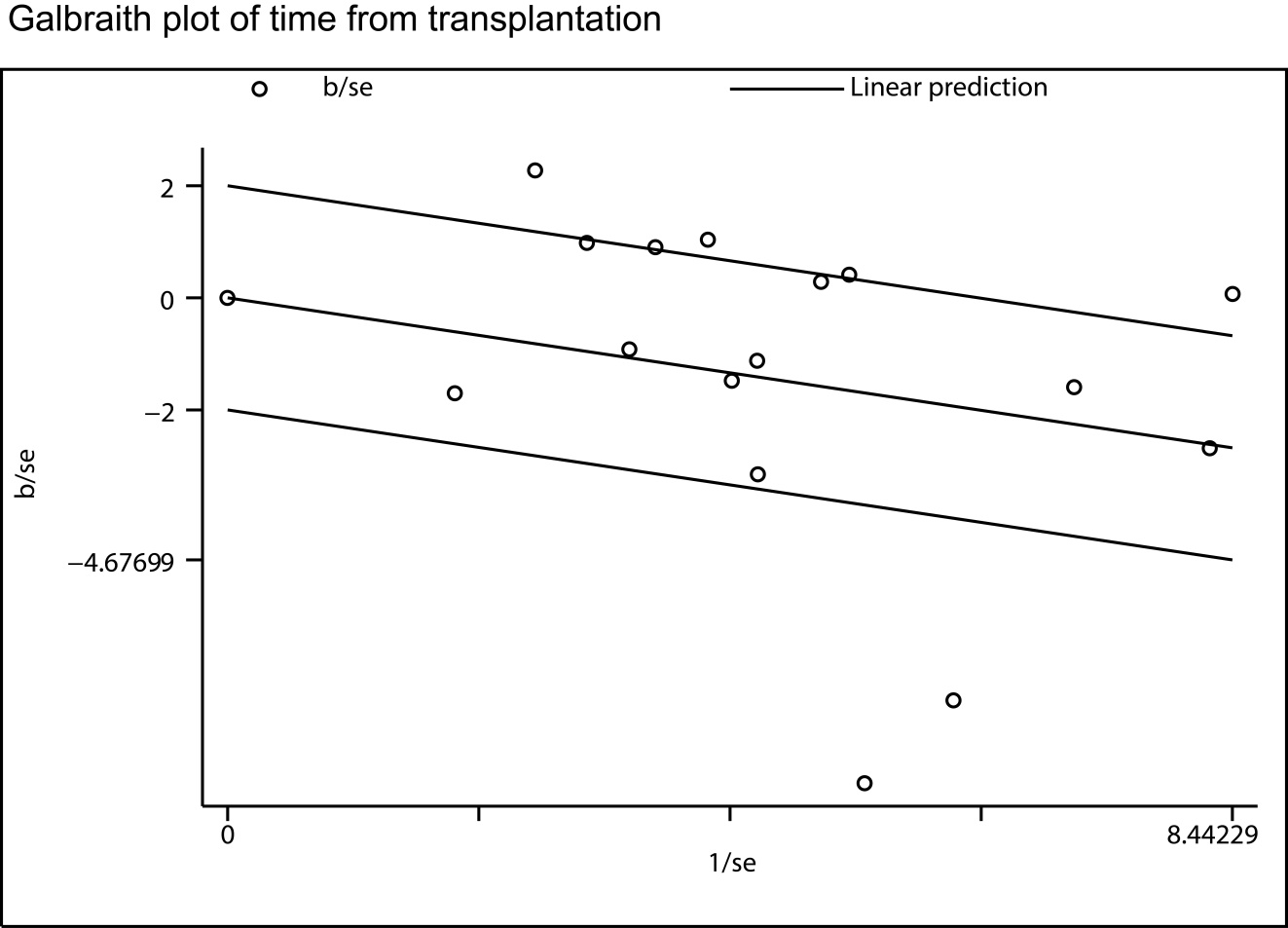

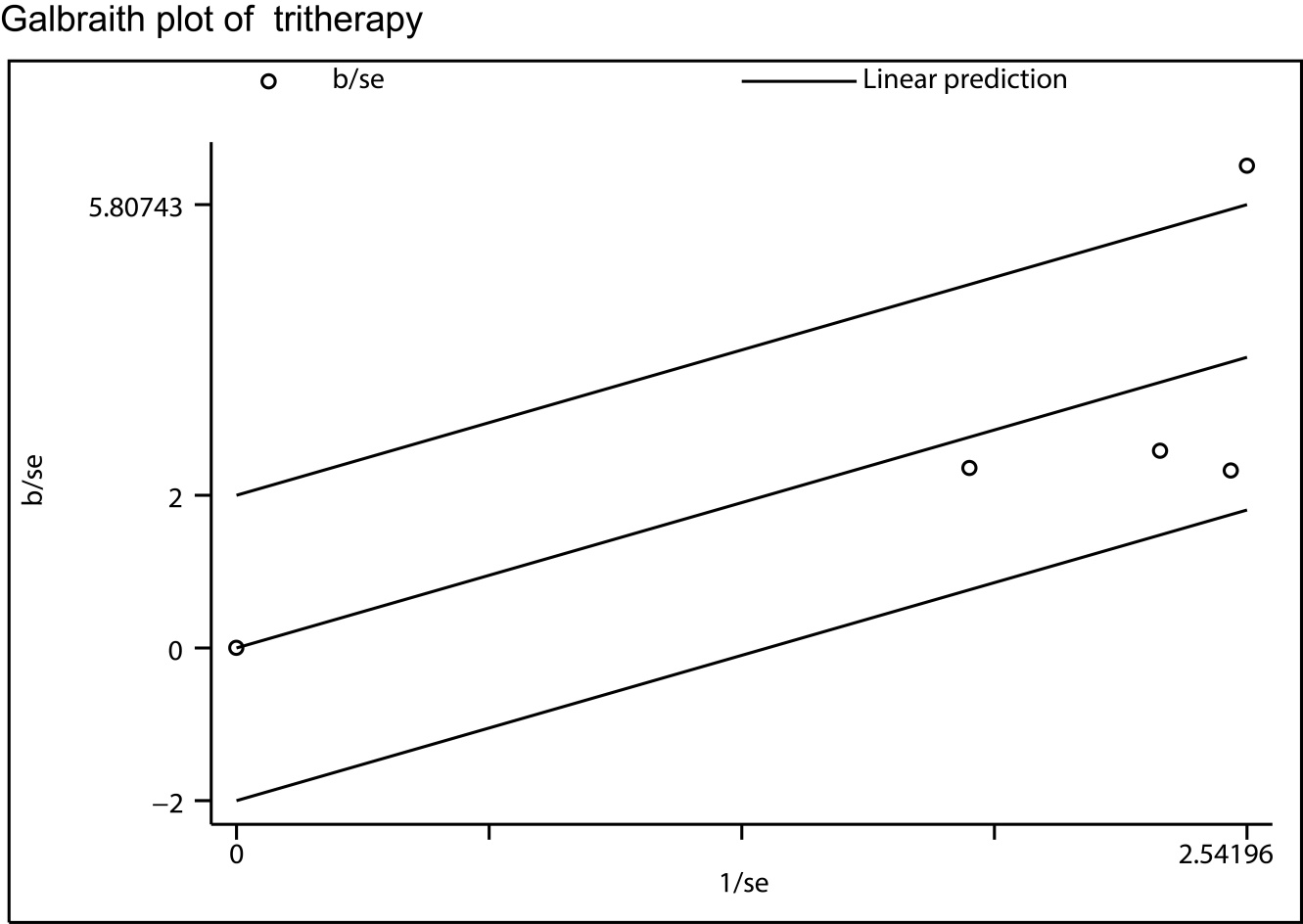


Sensitivity analysis


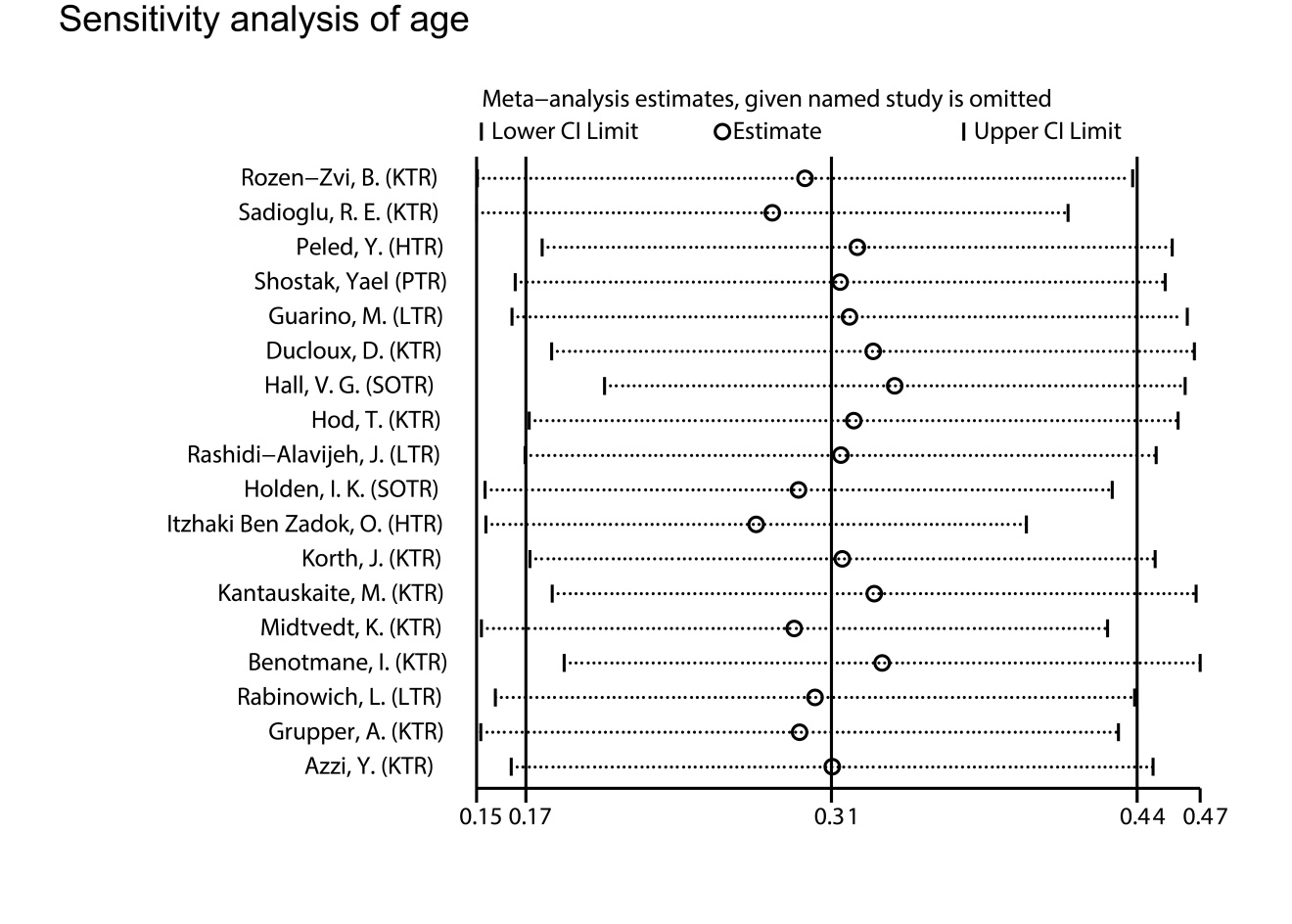

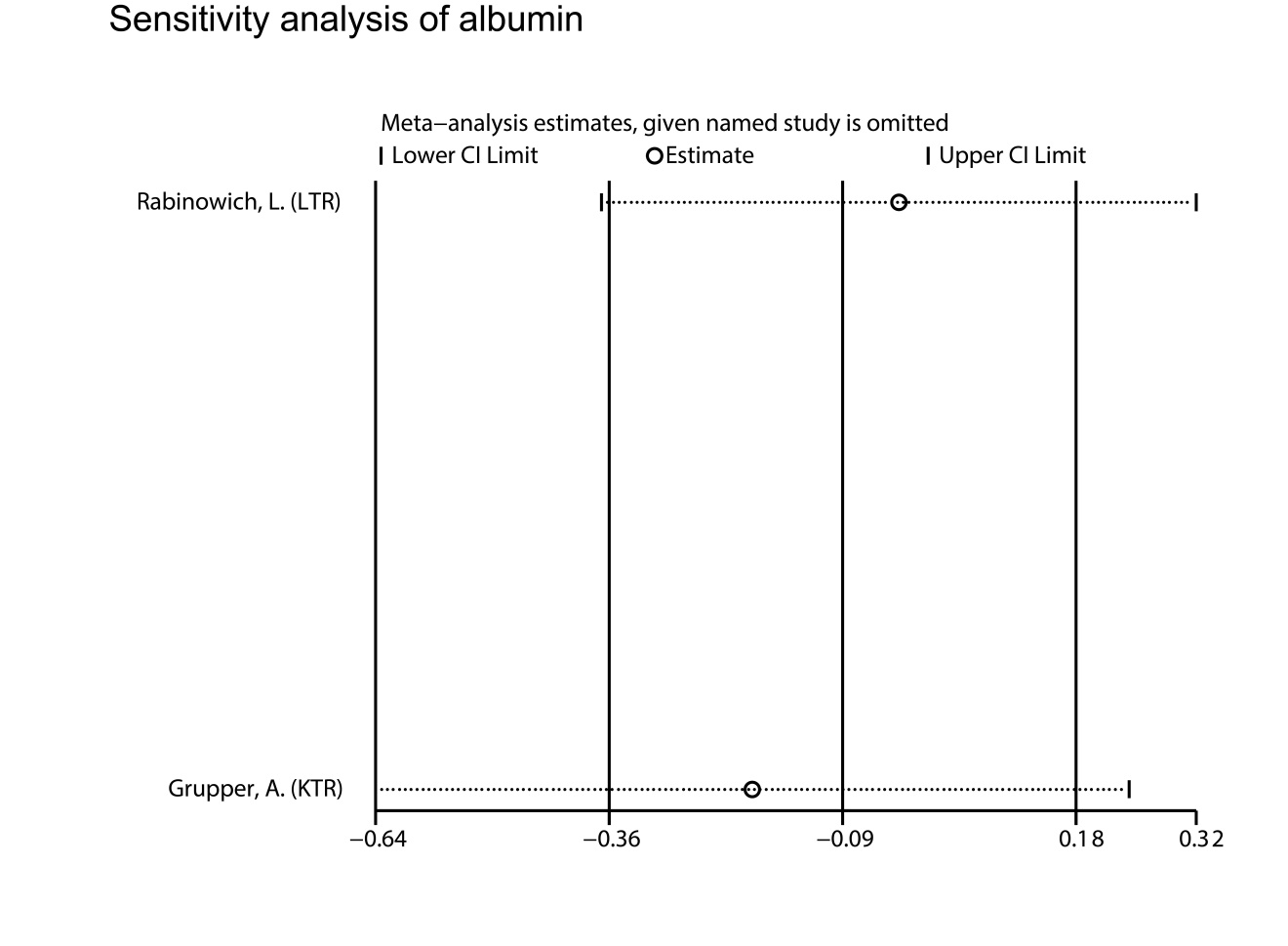

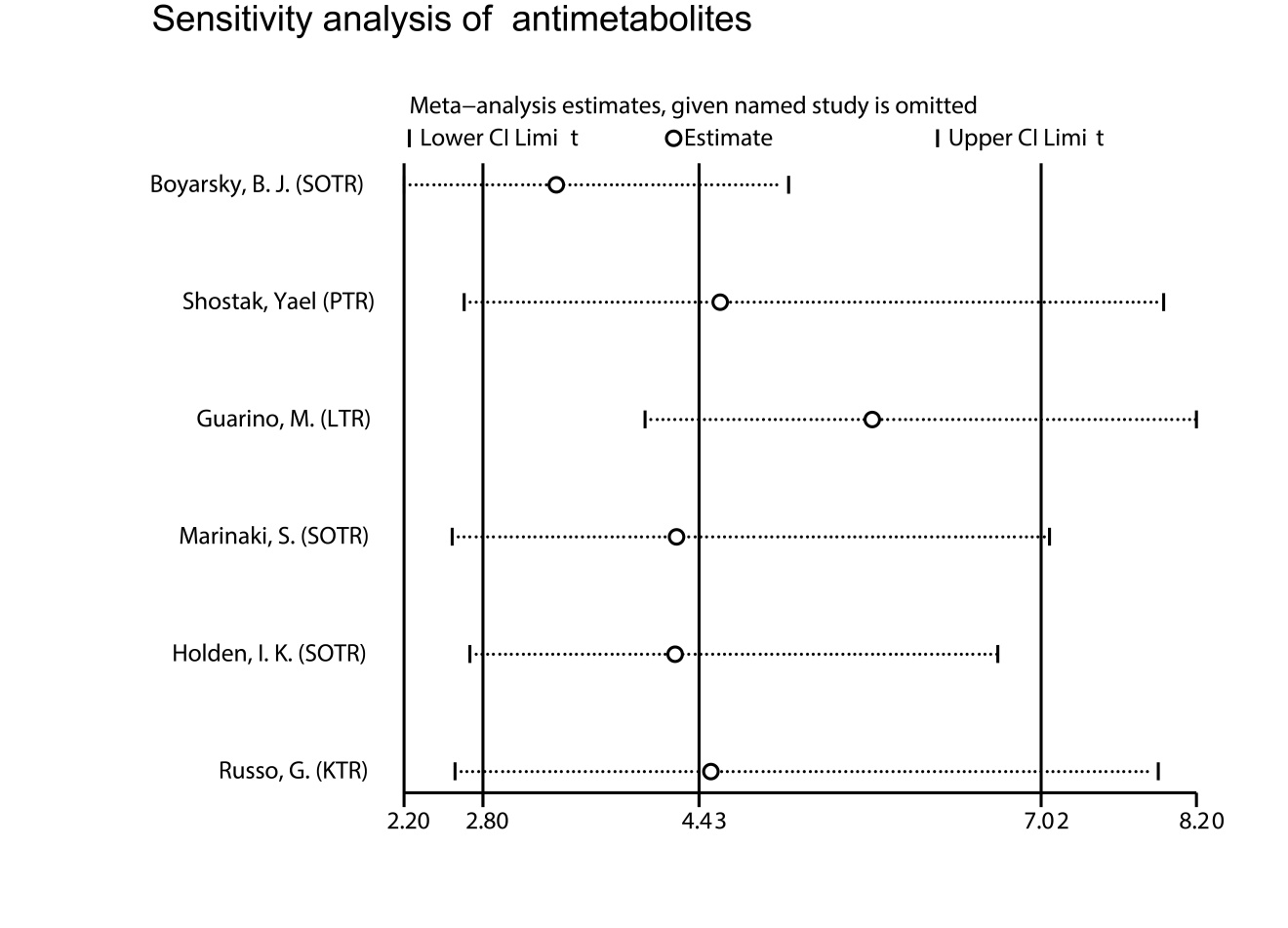

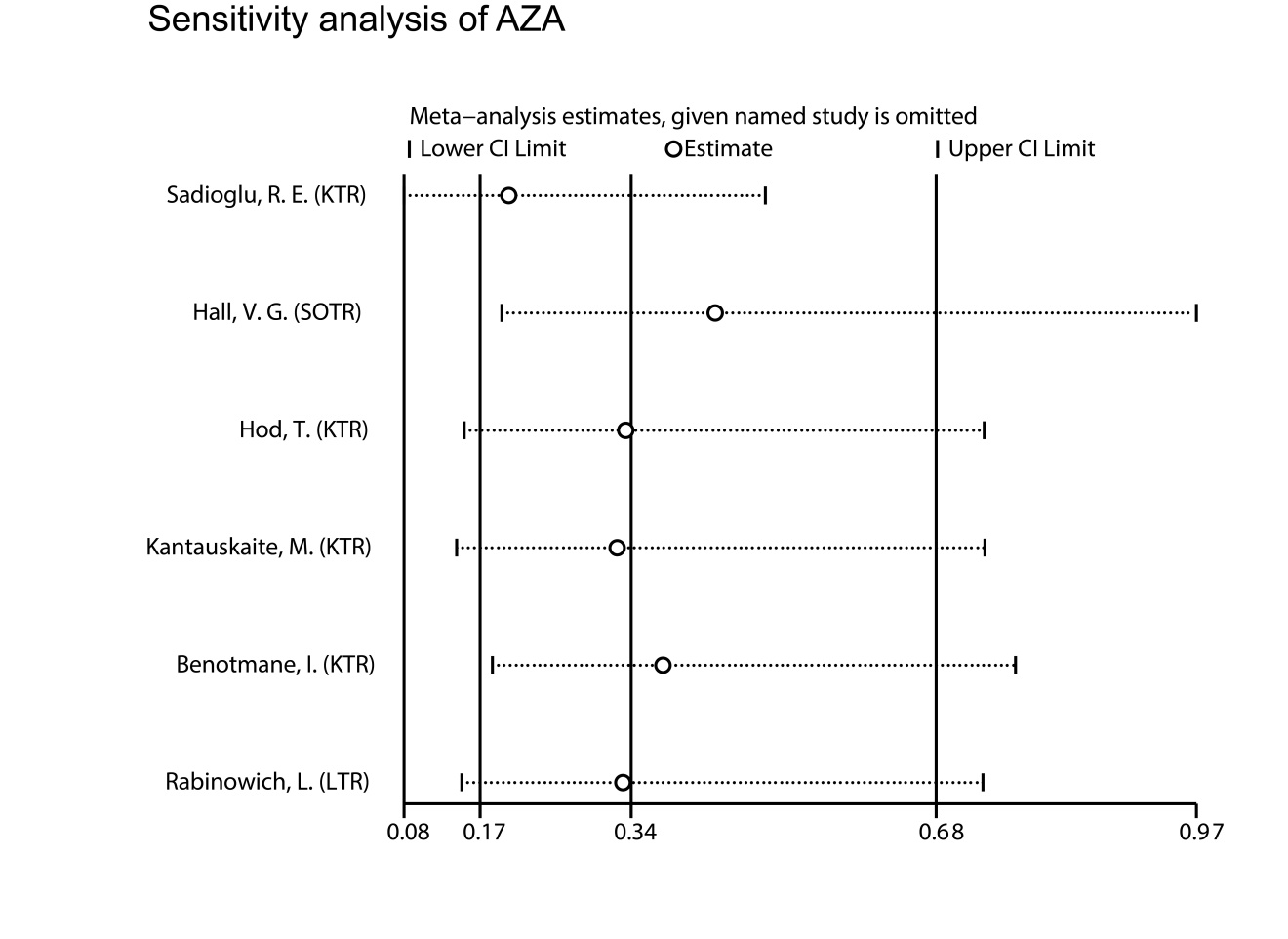

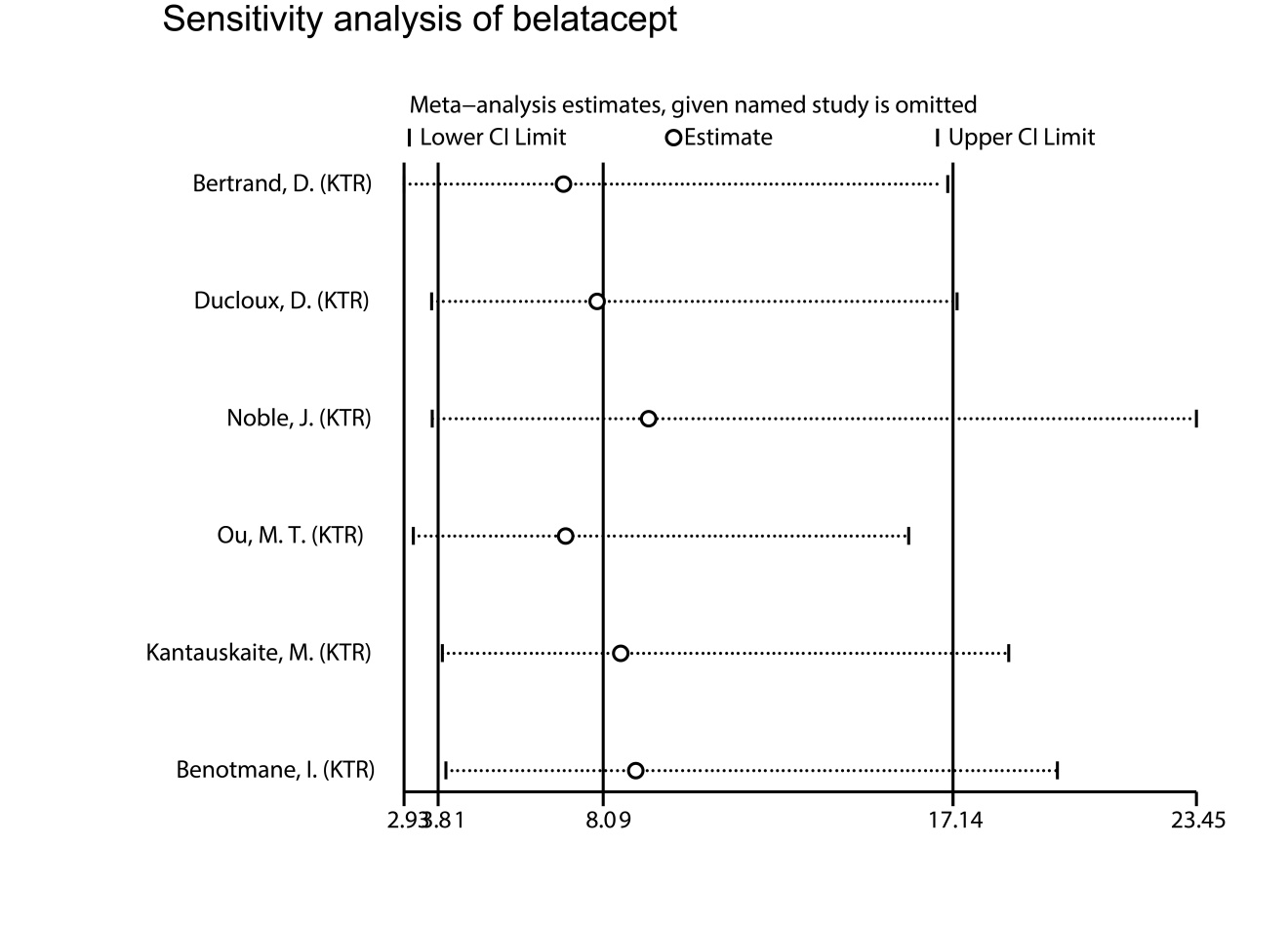

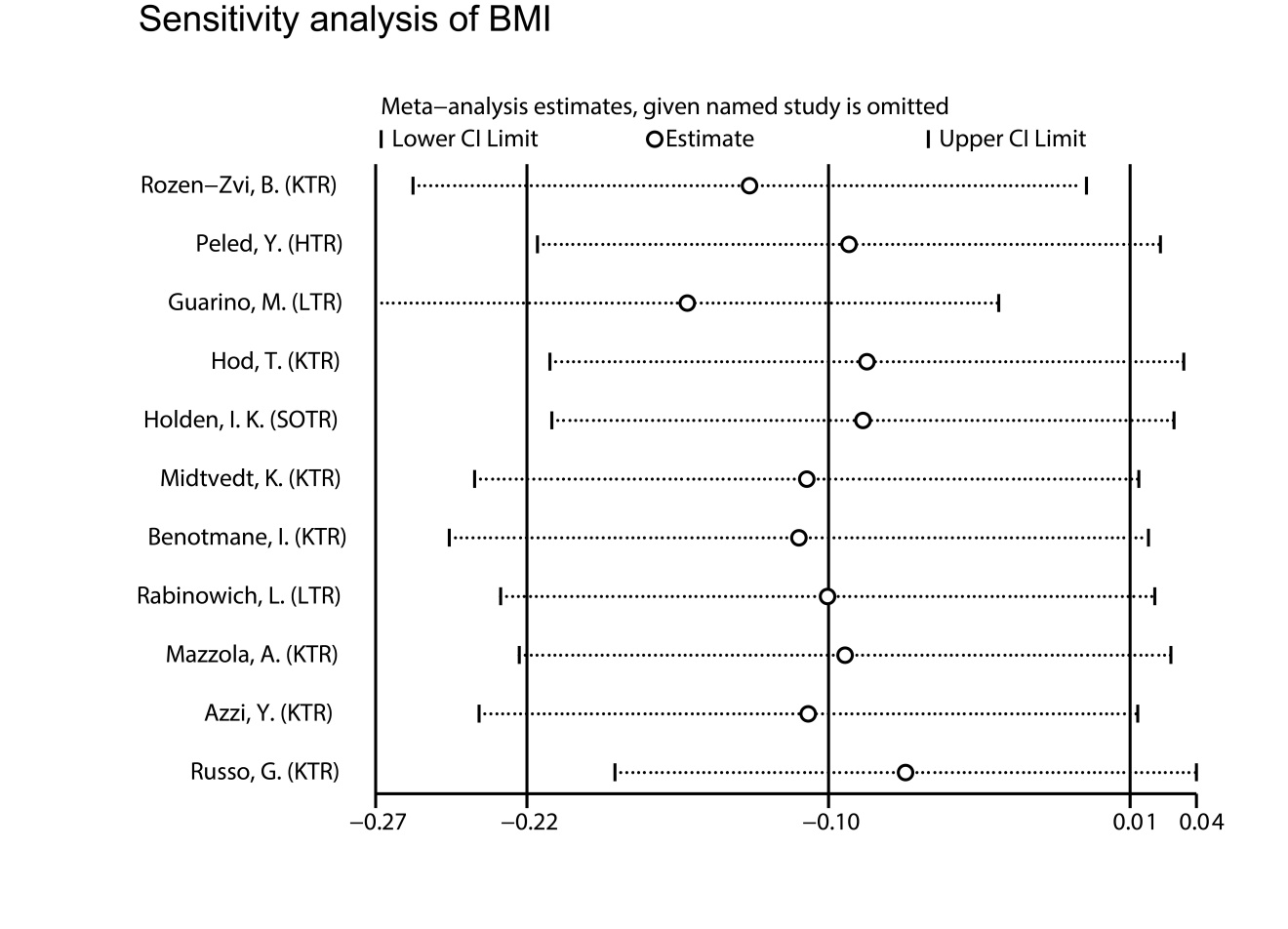

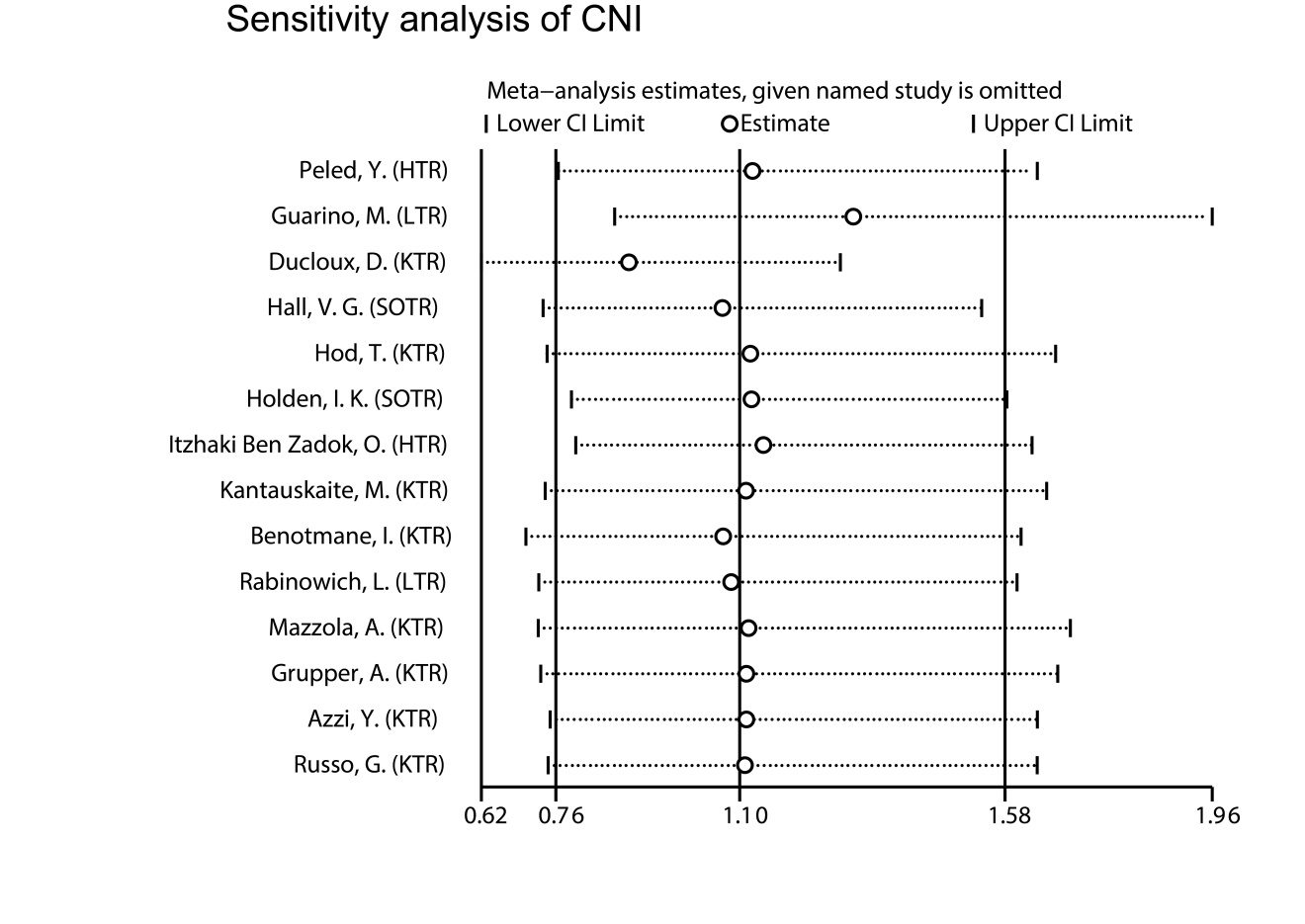

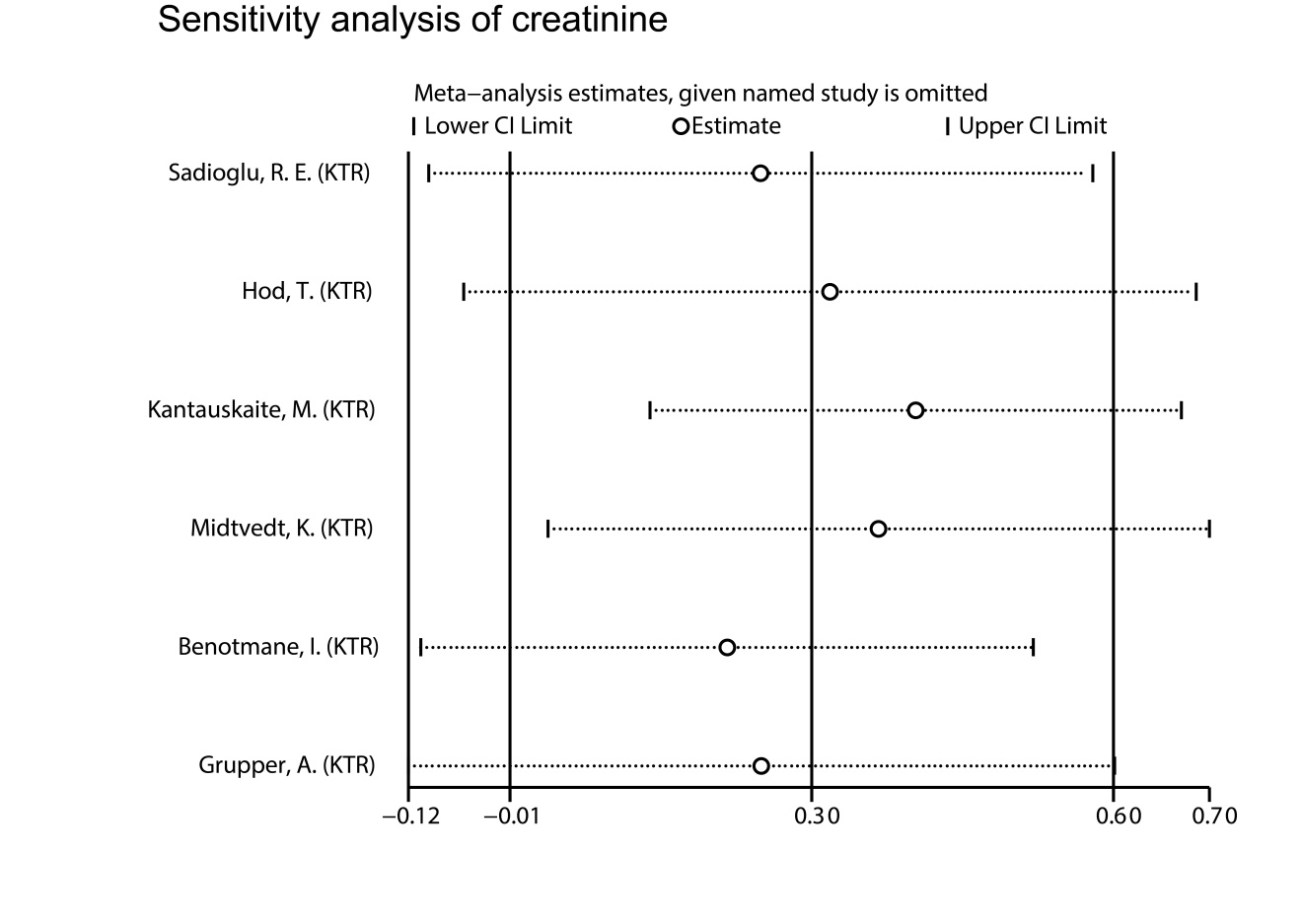

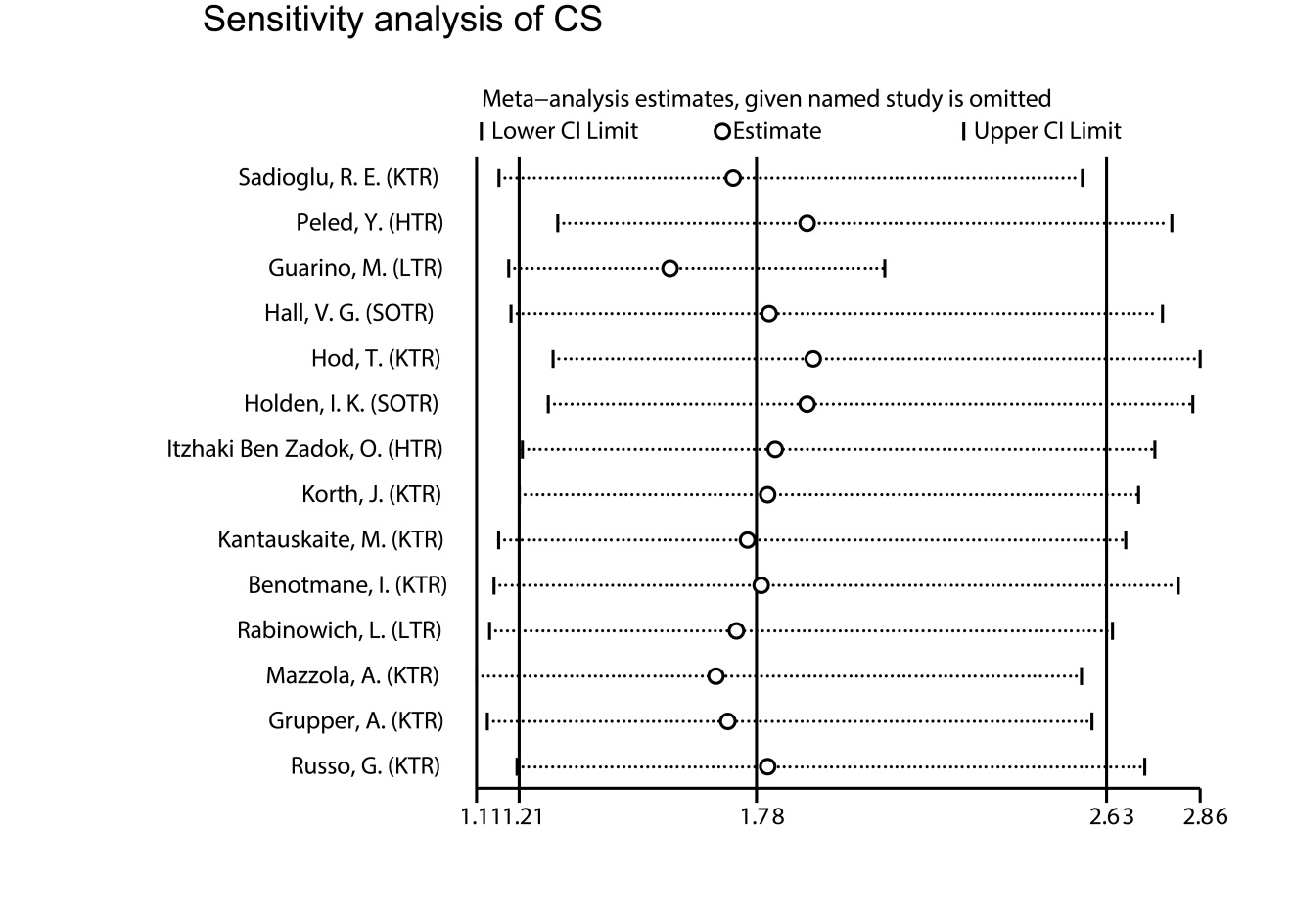

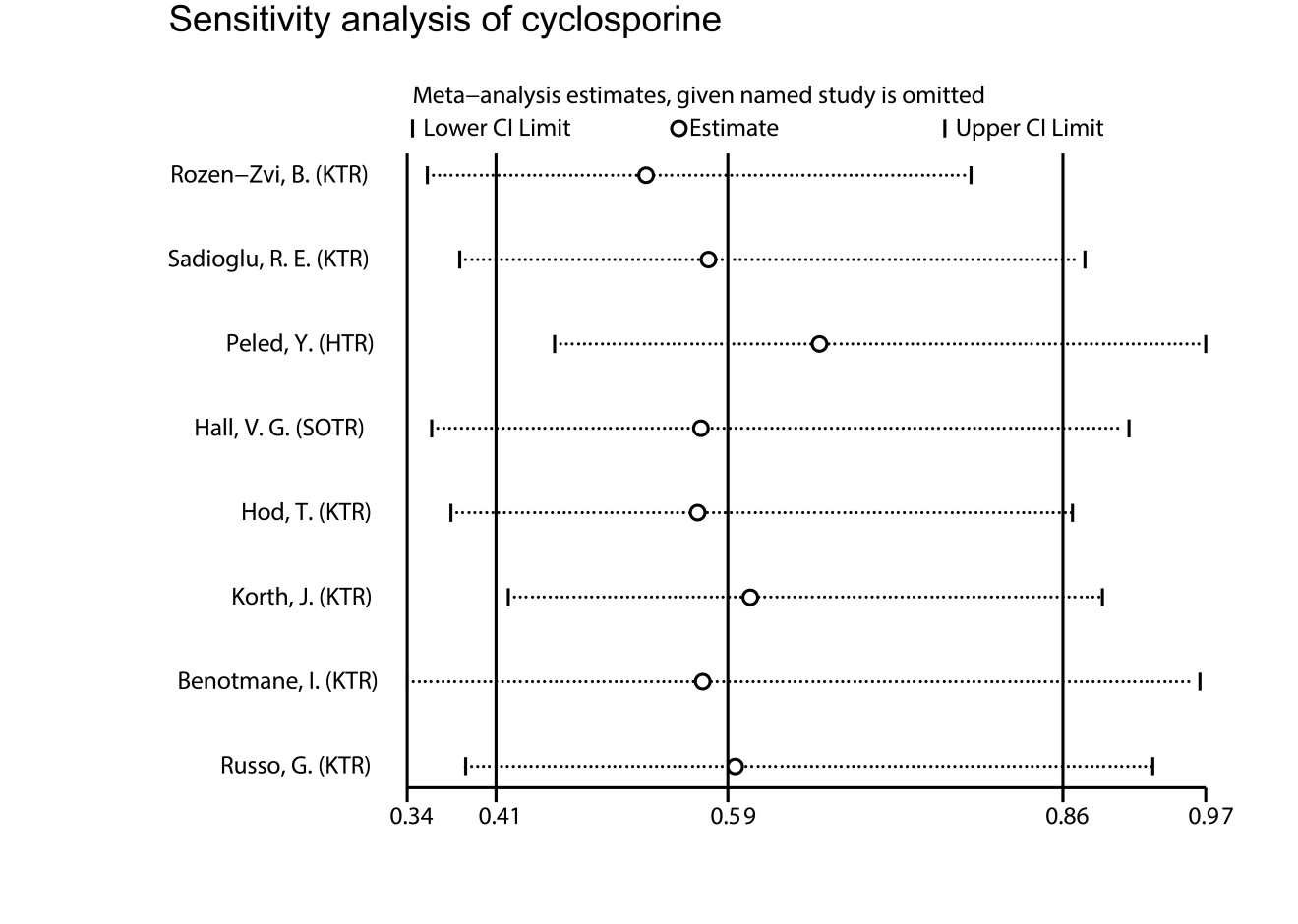

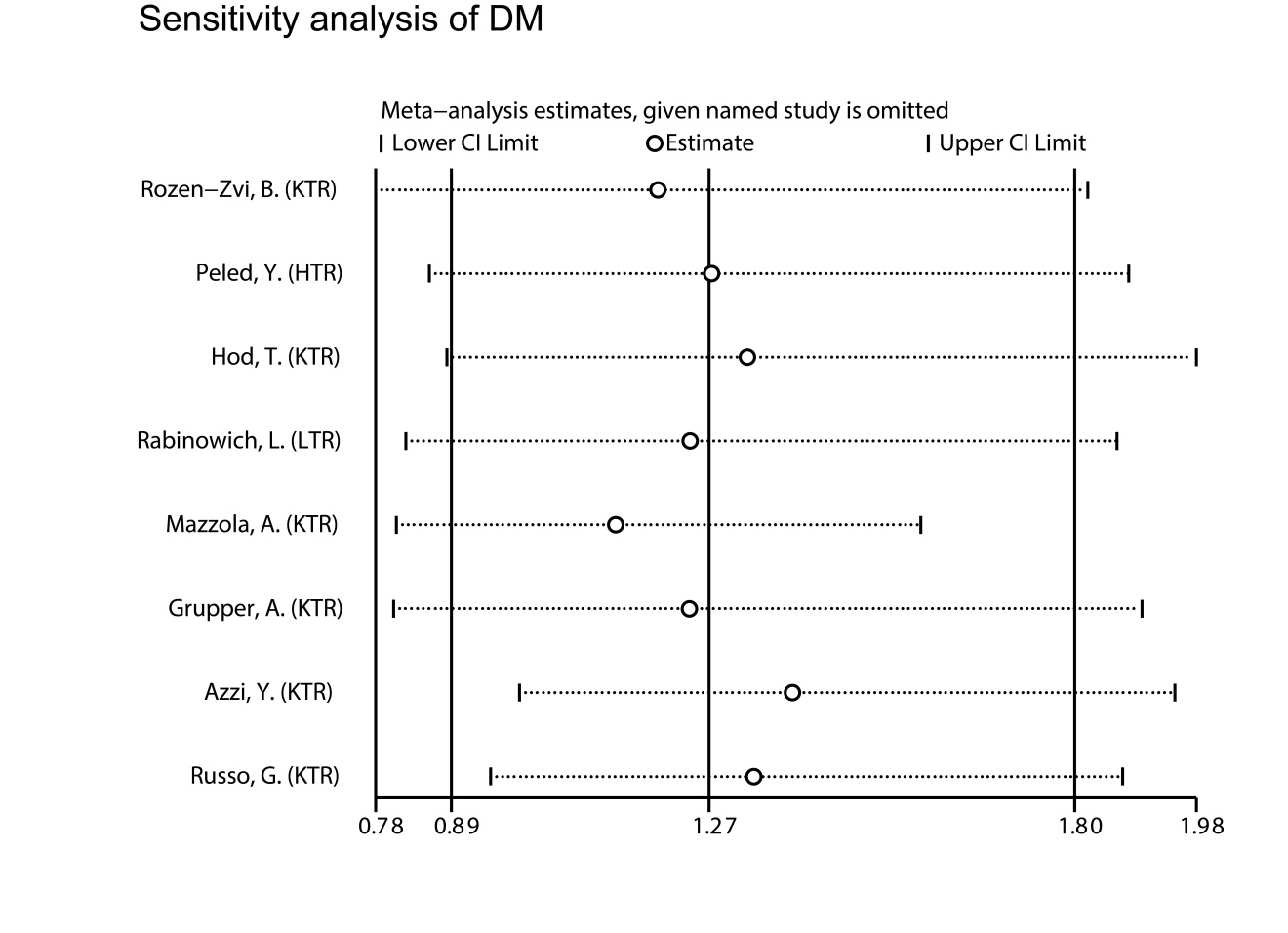

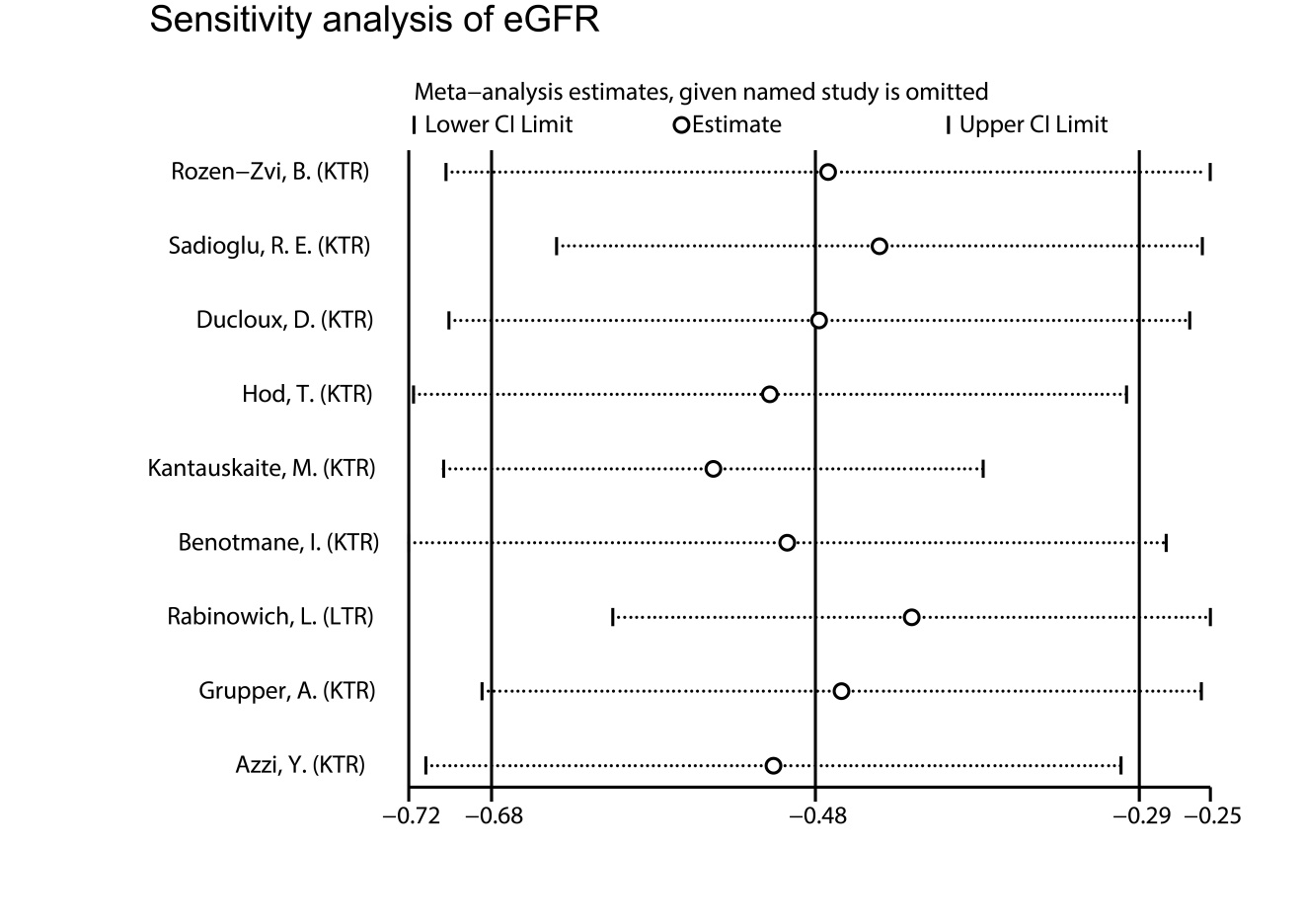

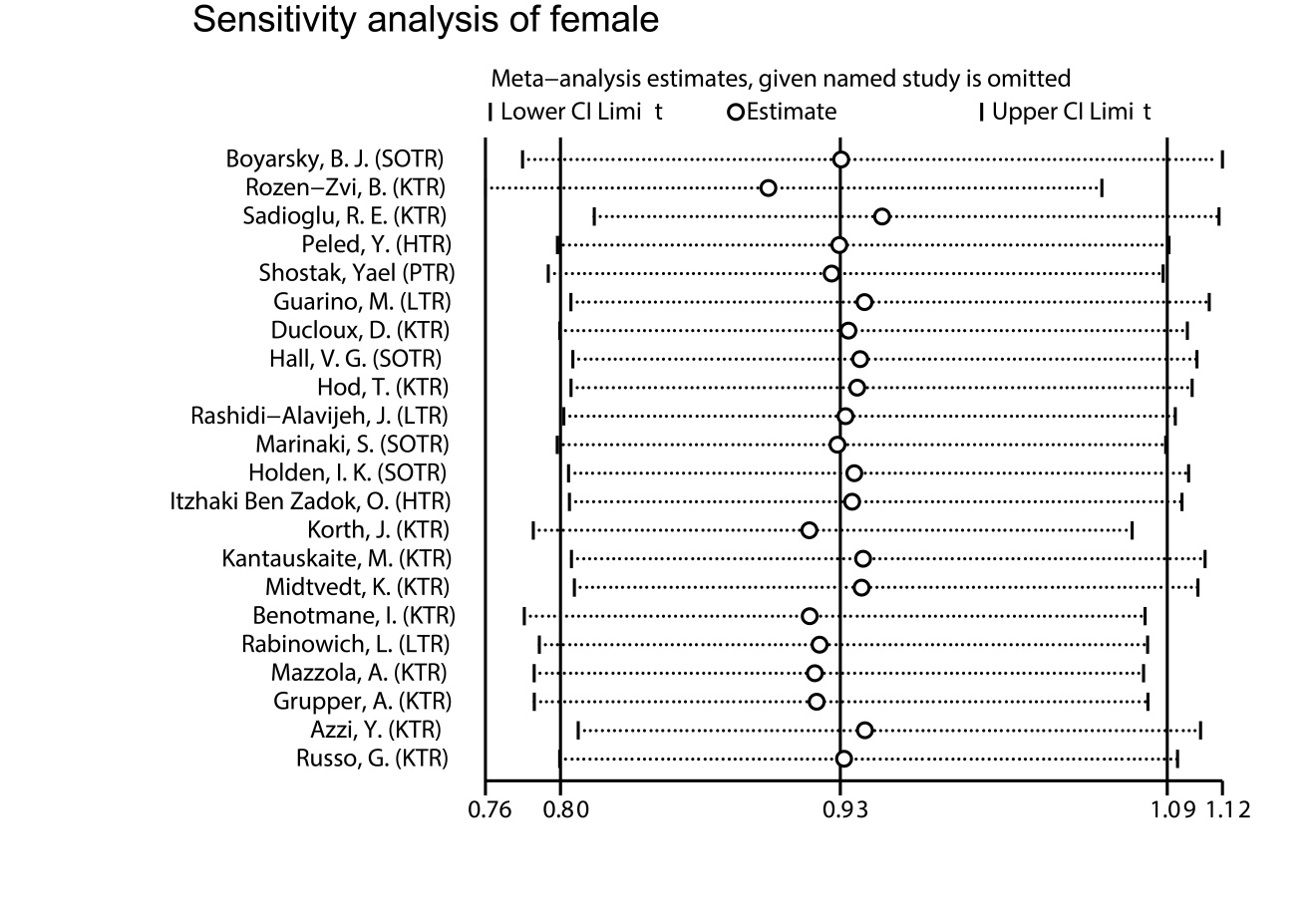

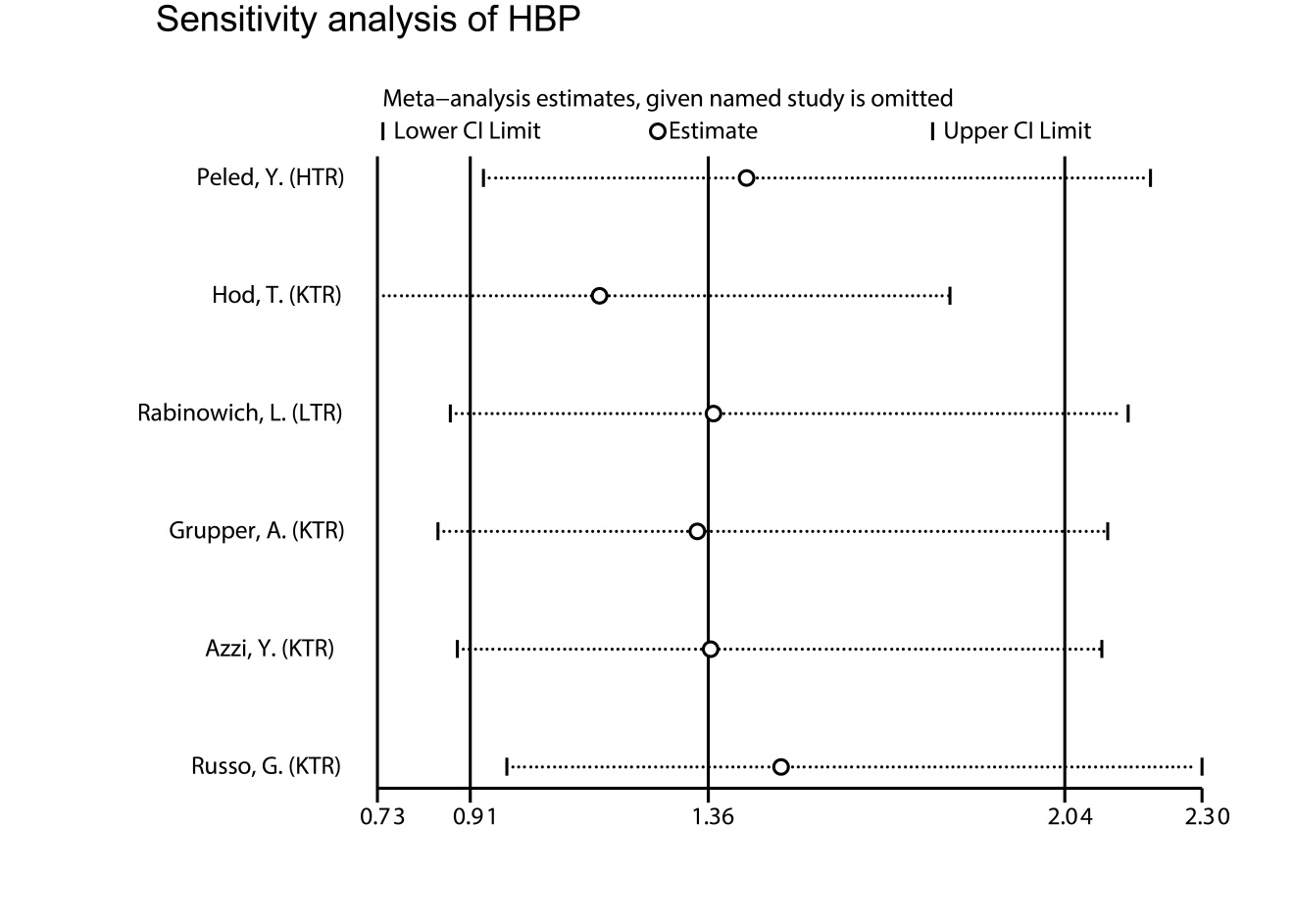

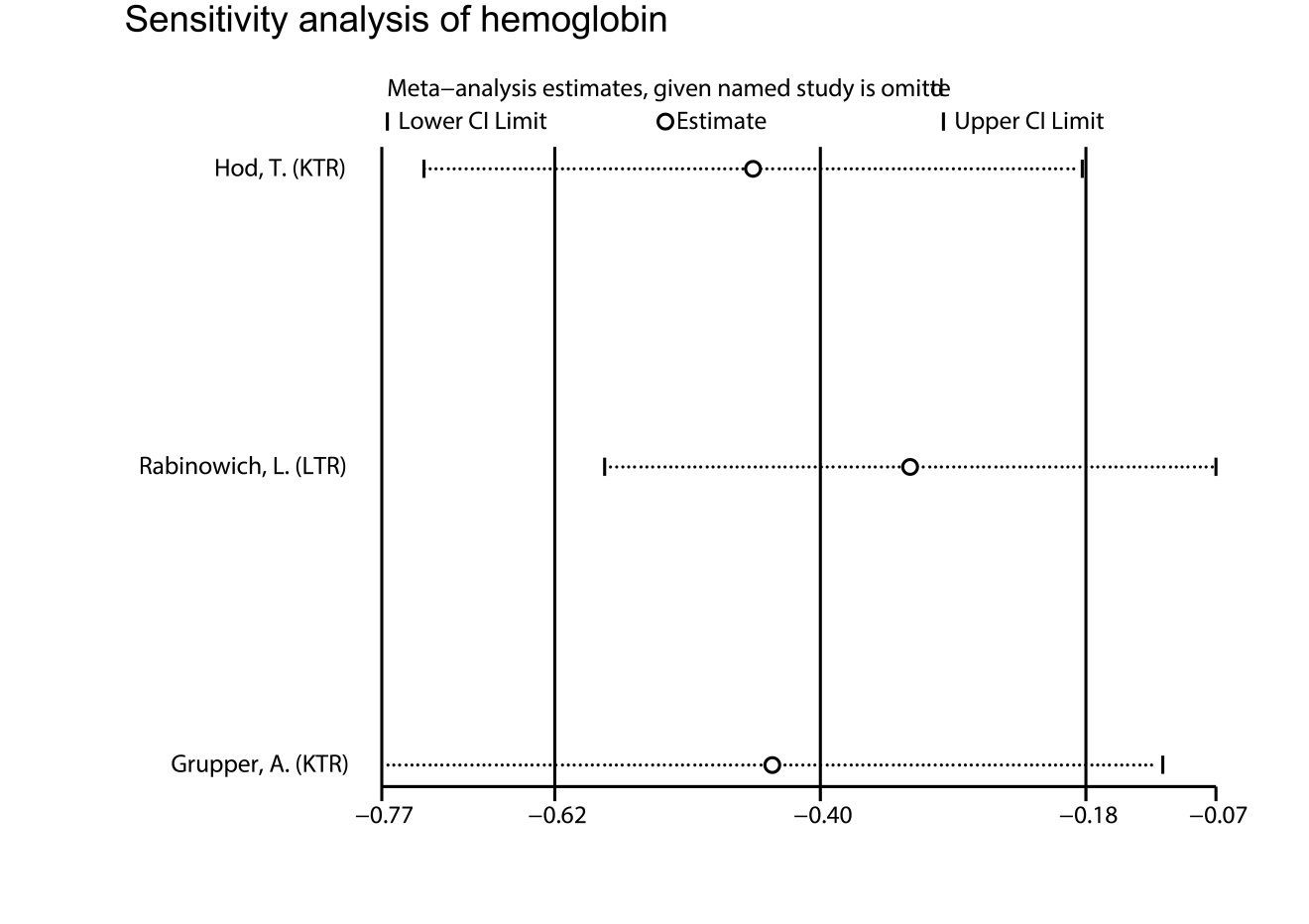

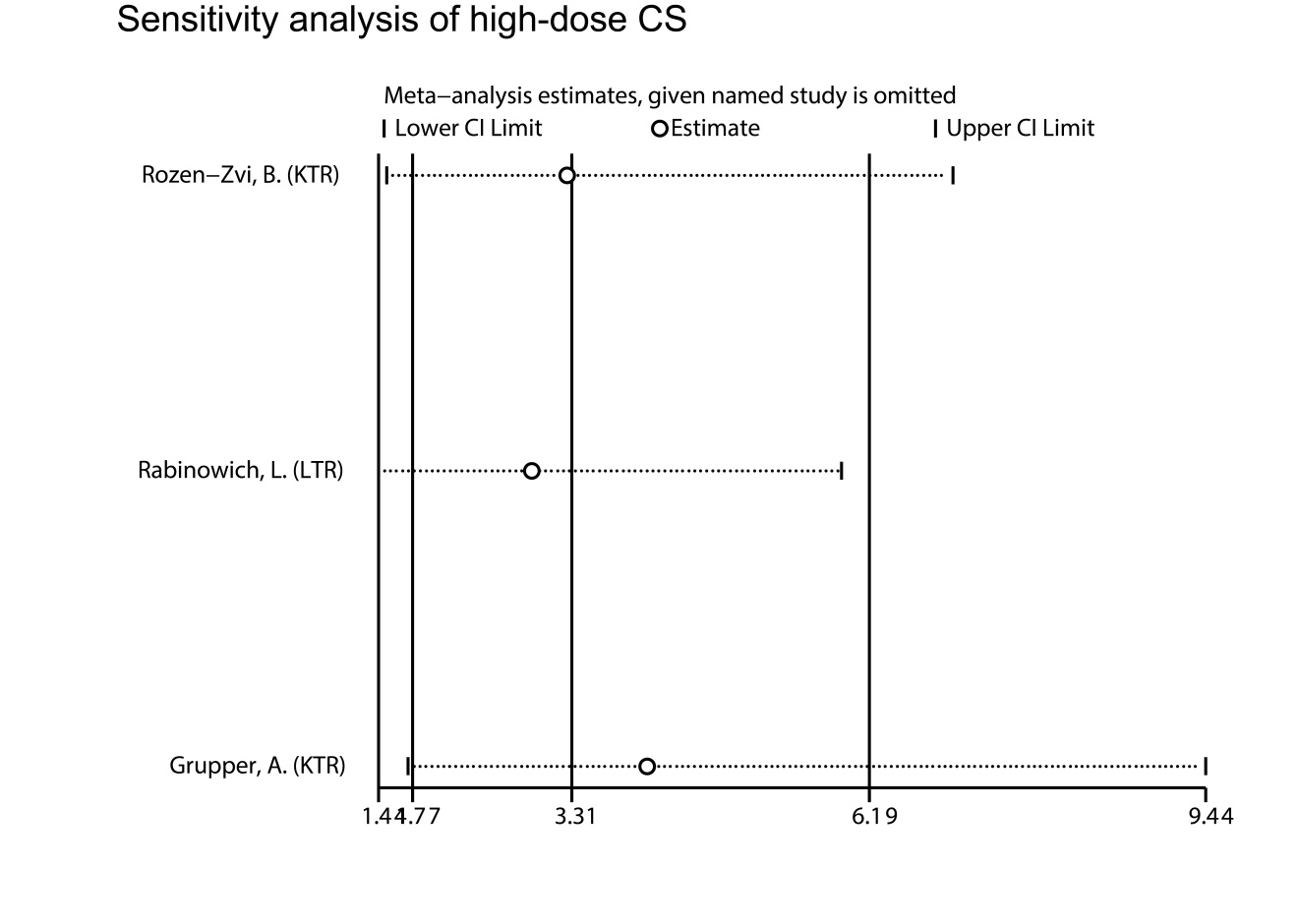

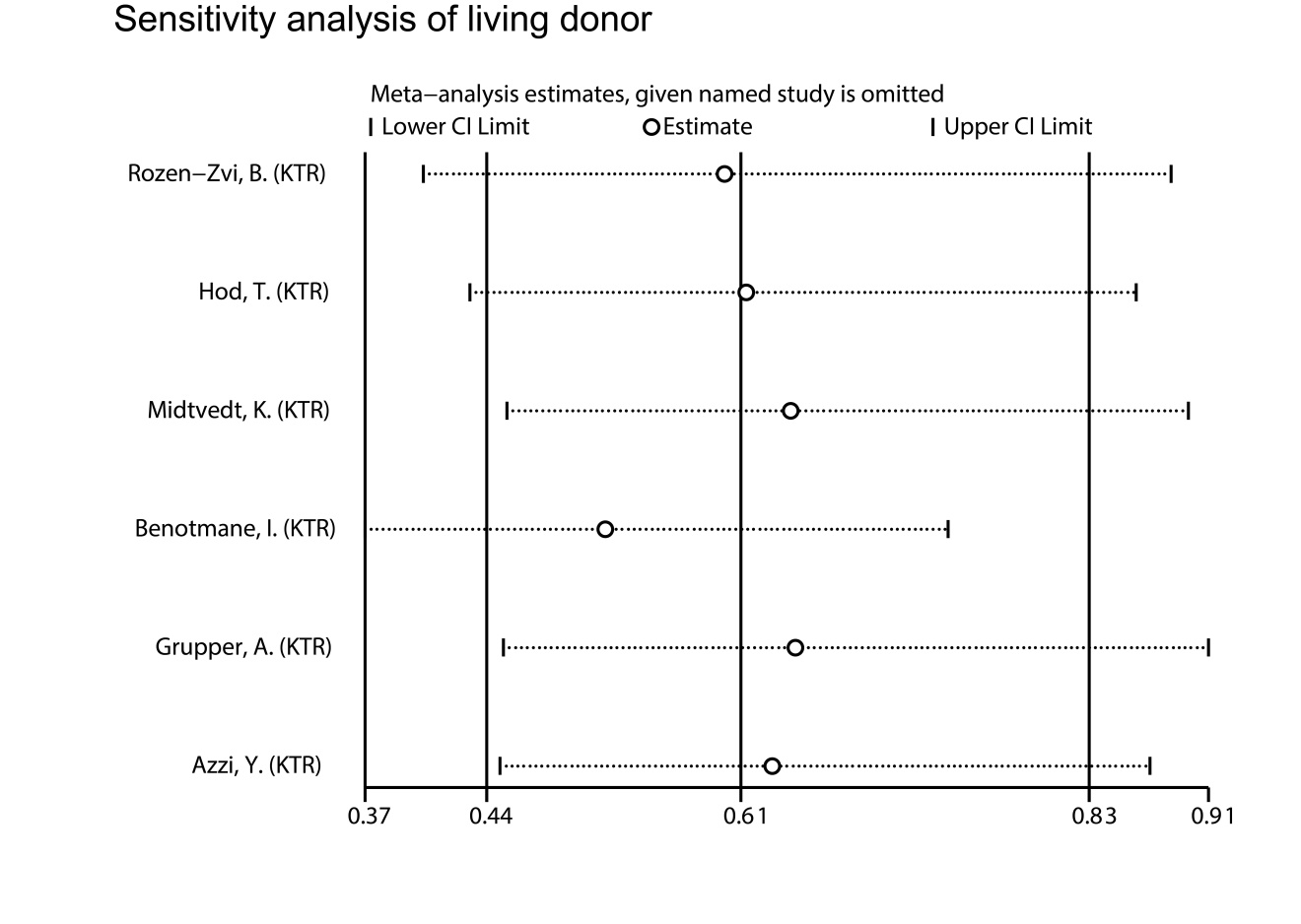

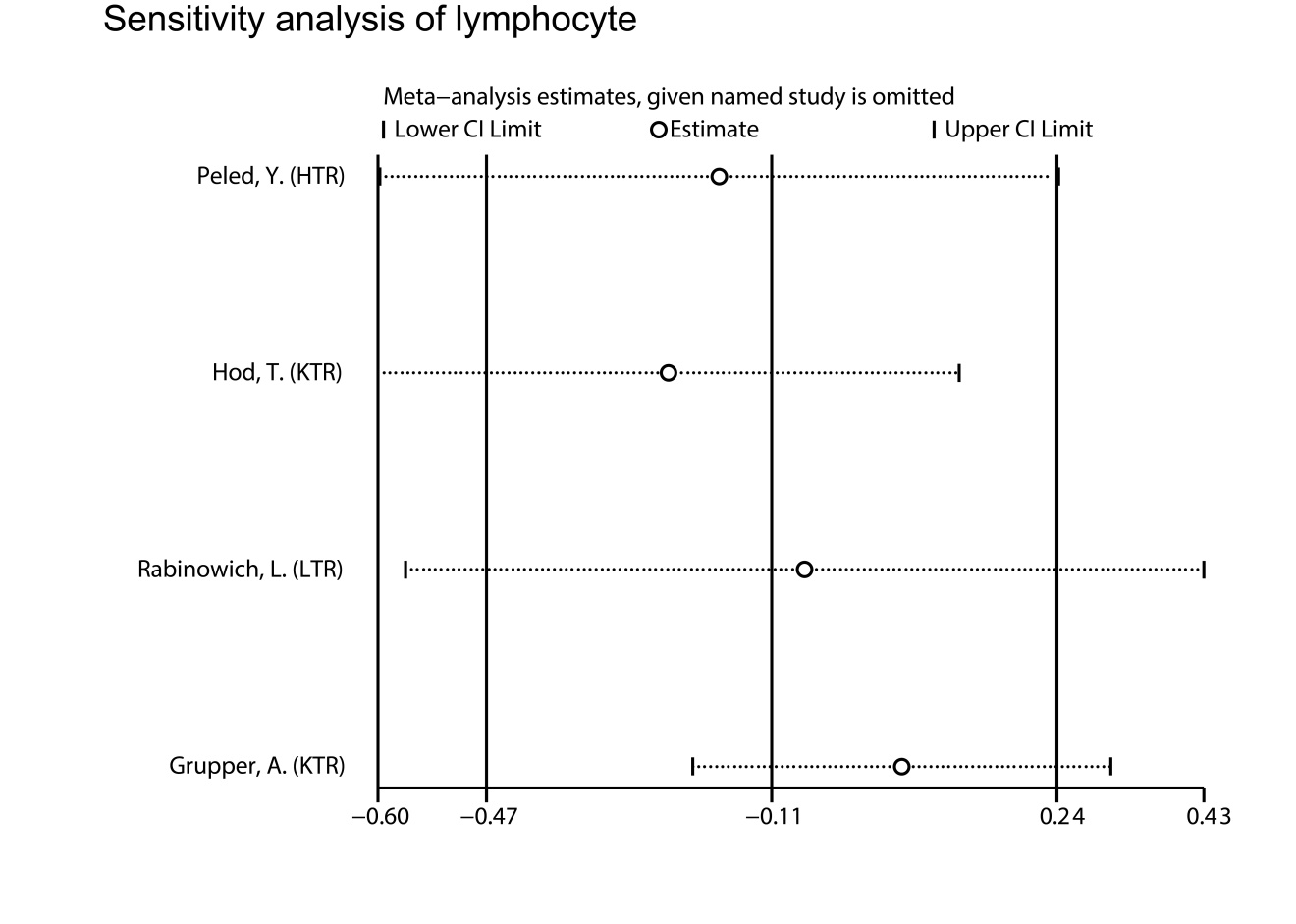

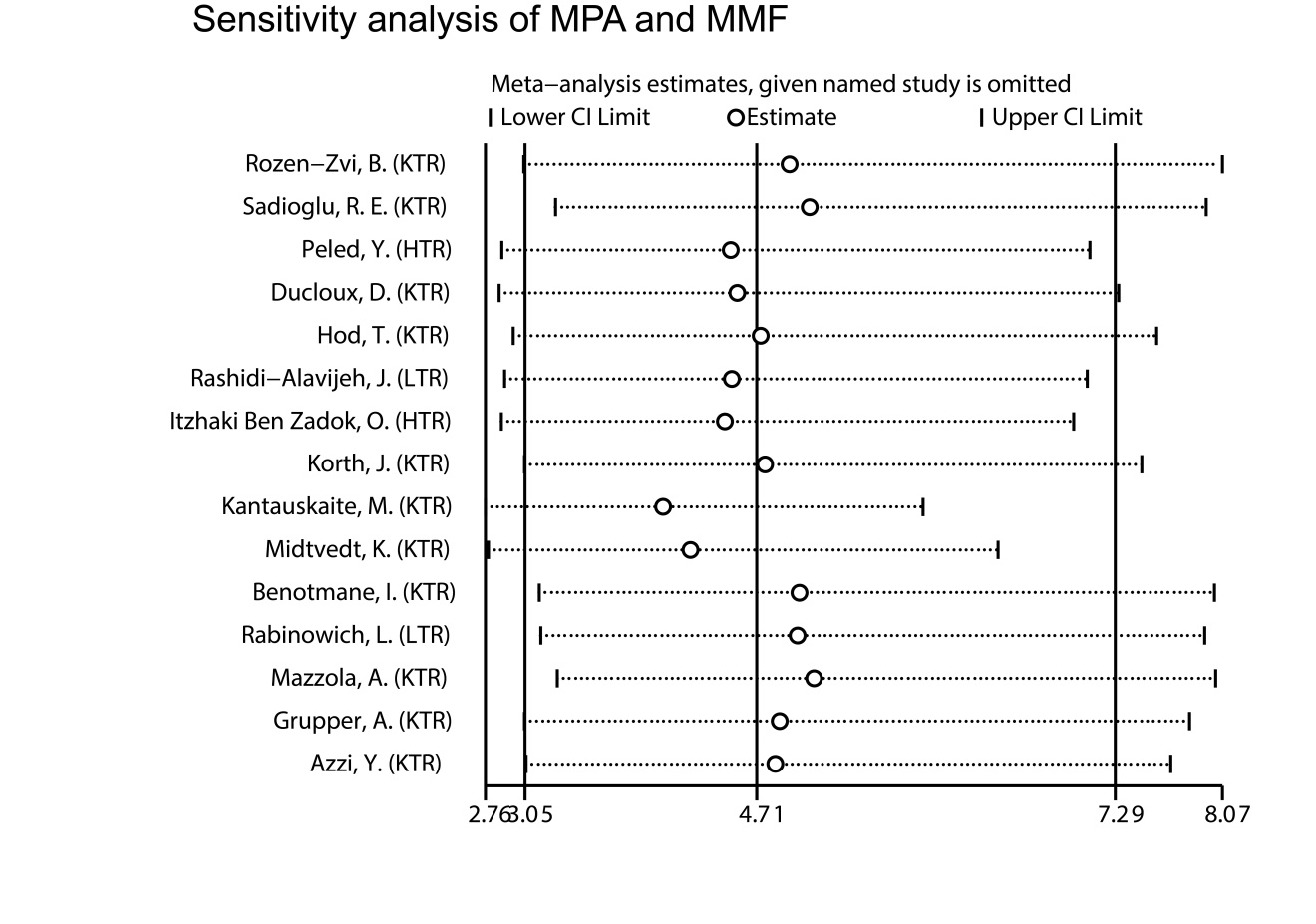

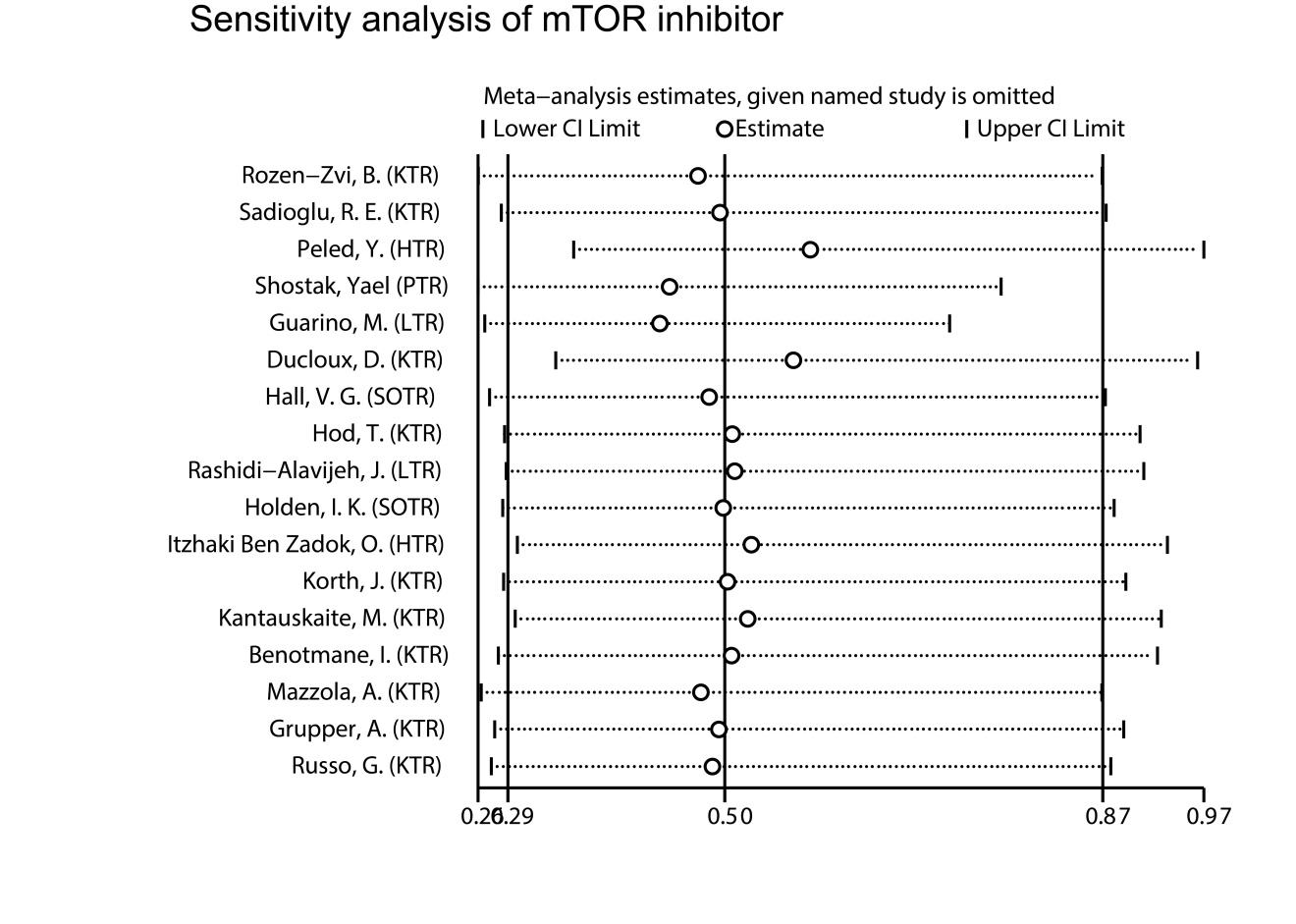

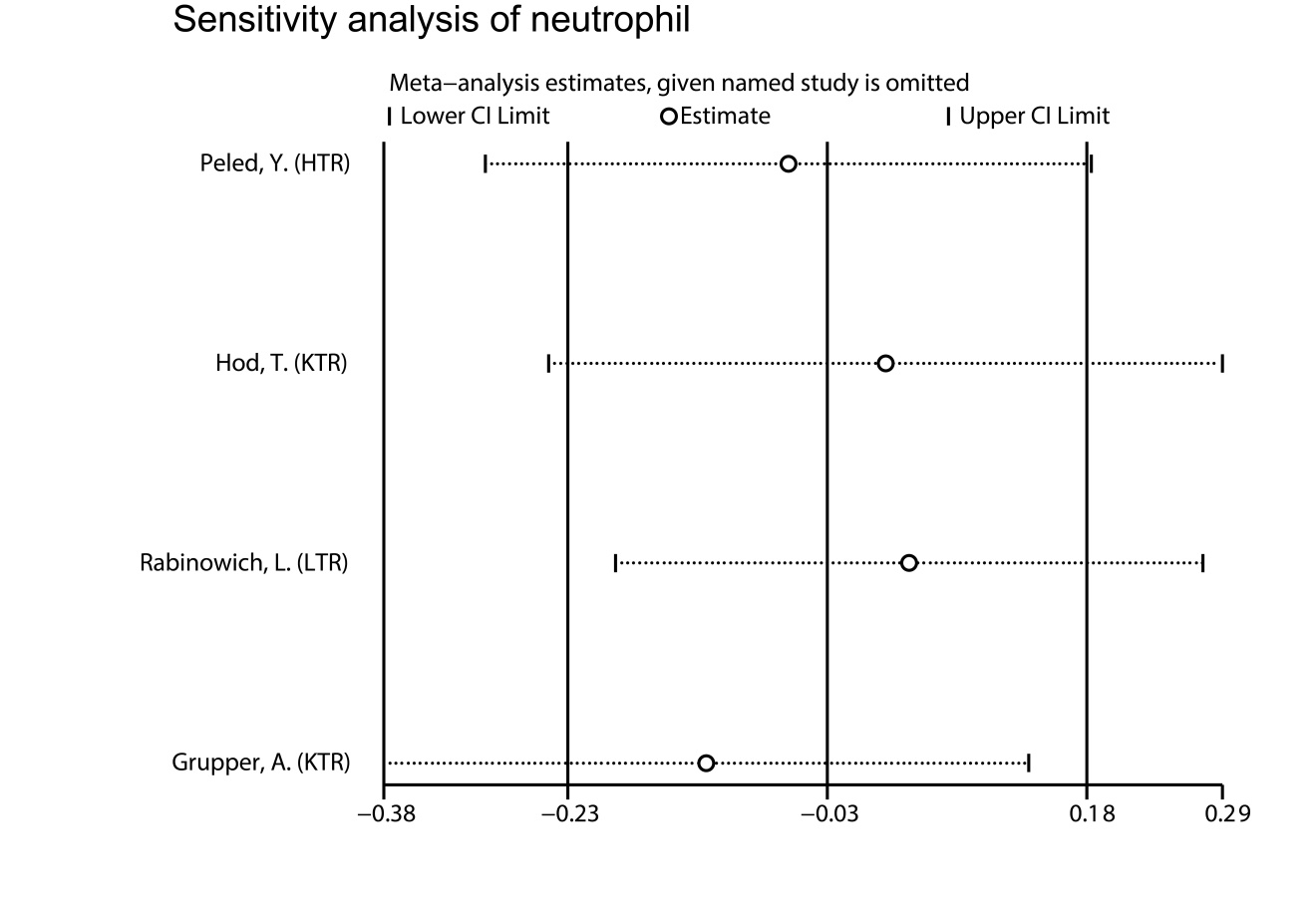

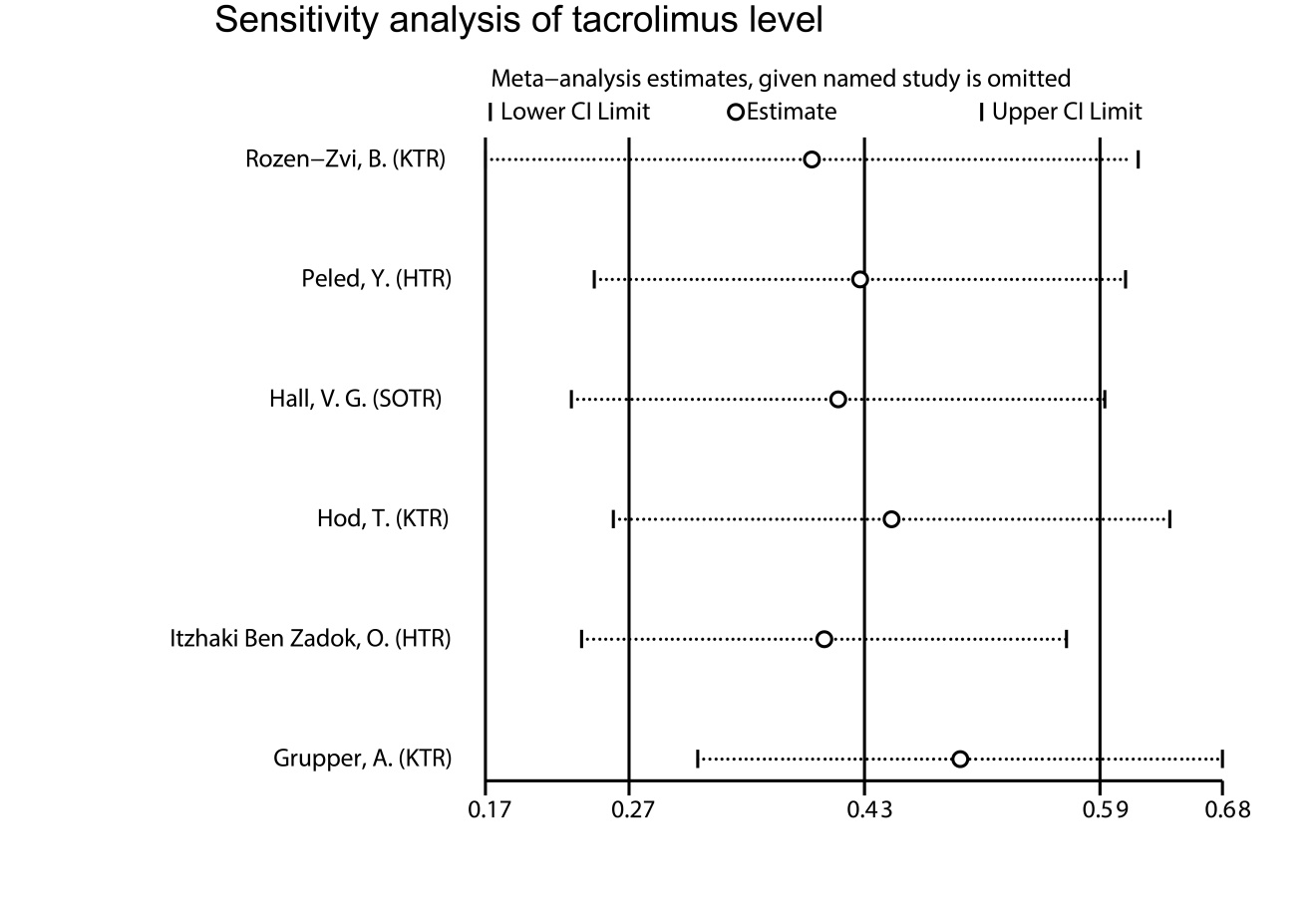

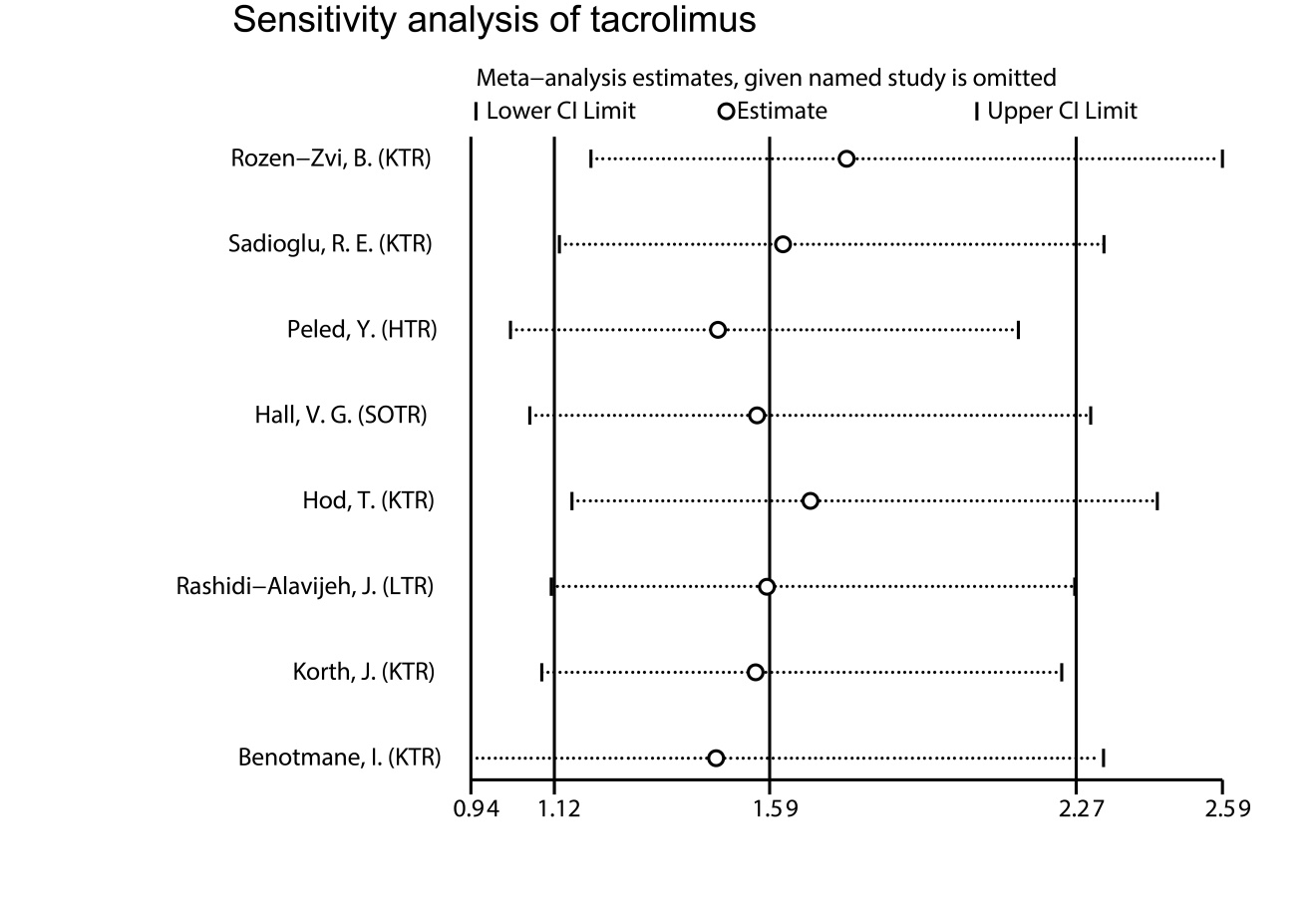

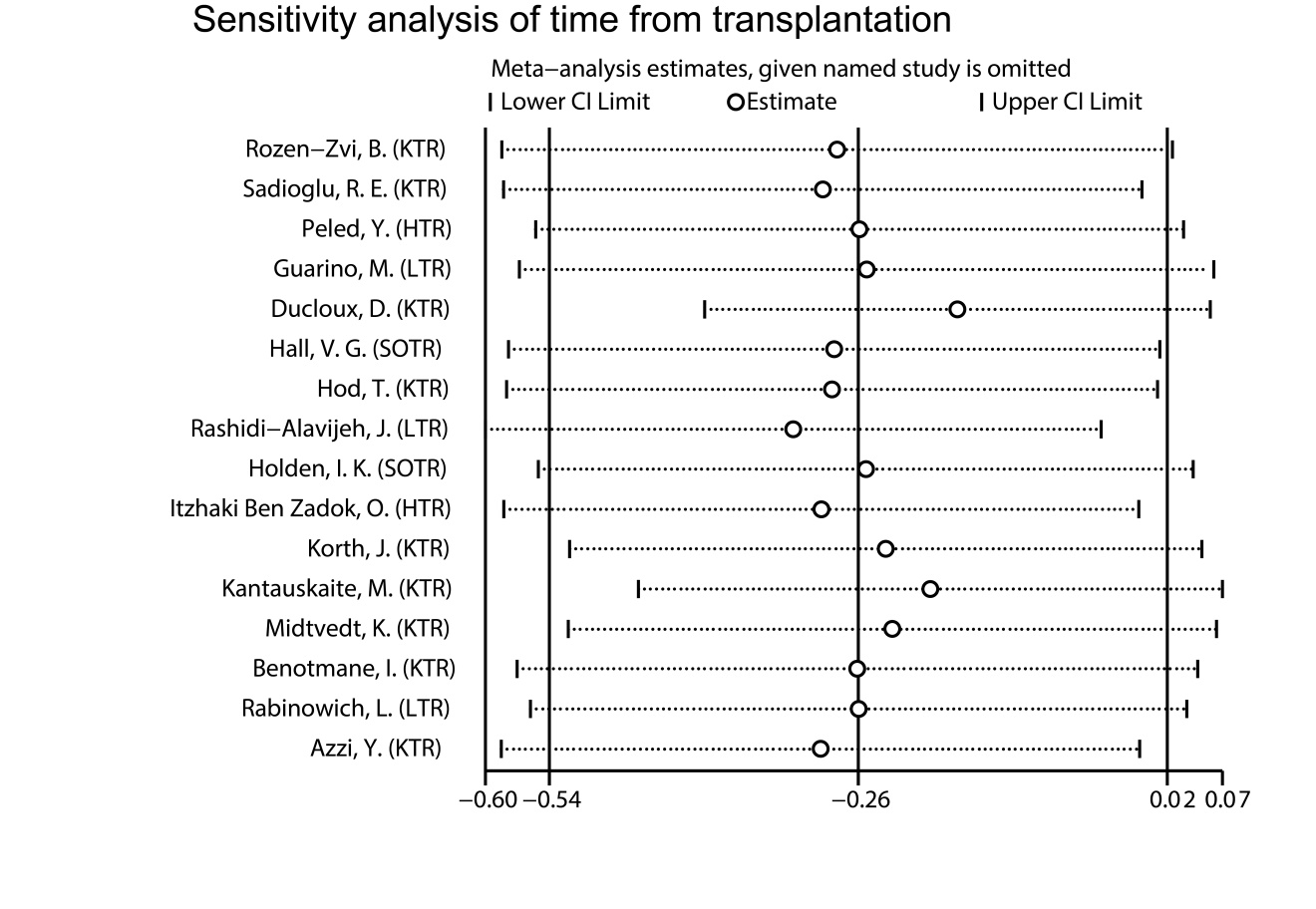

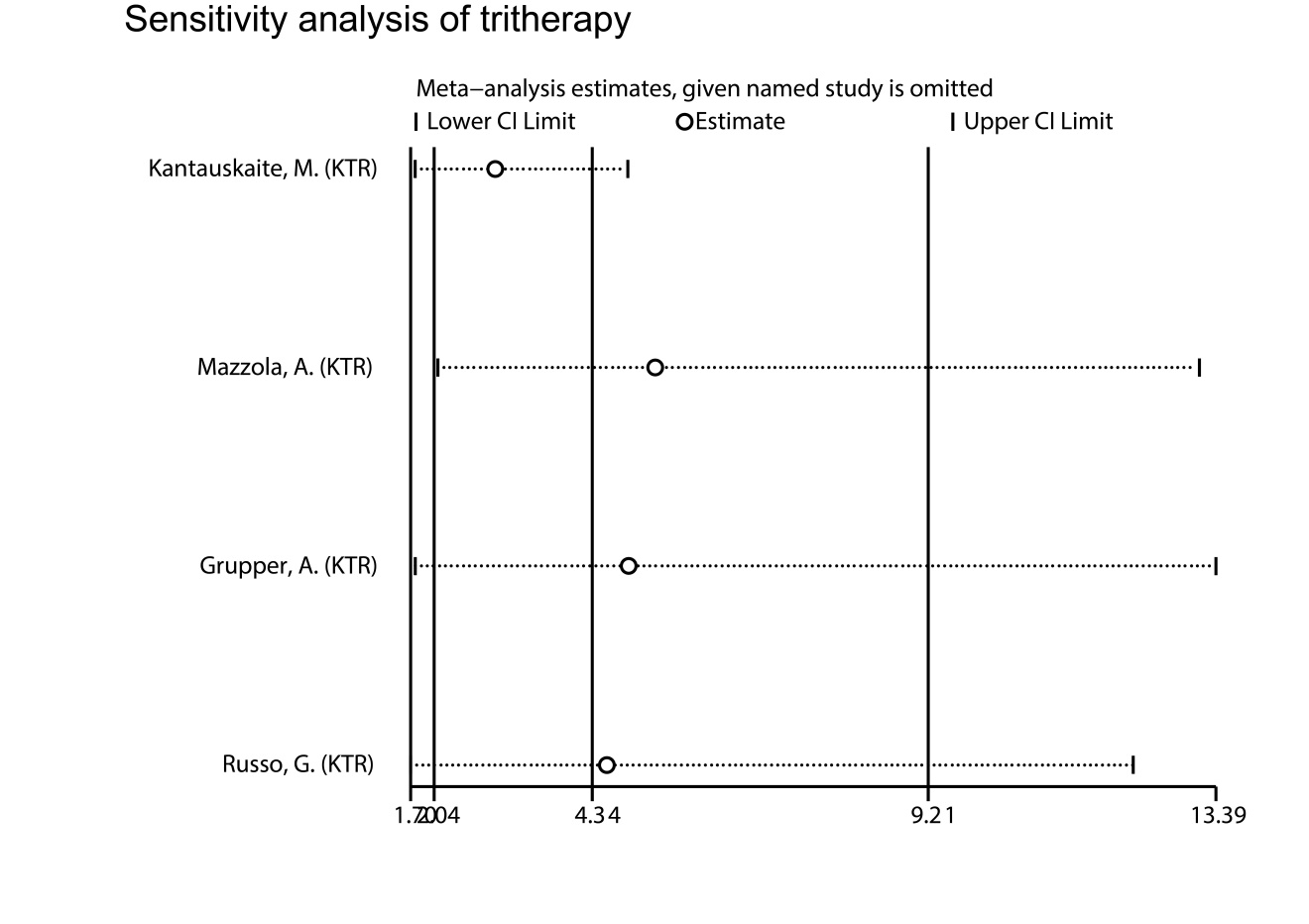

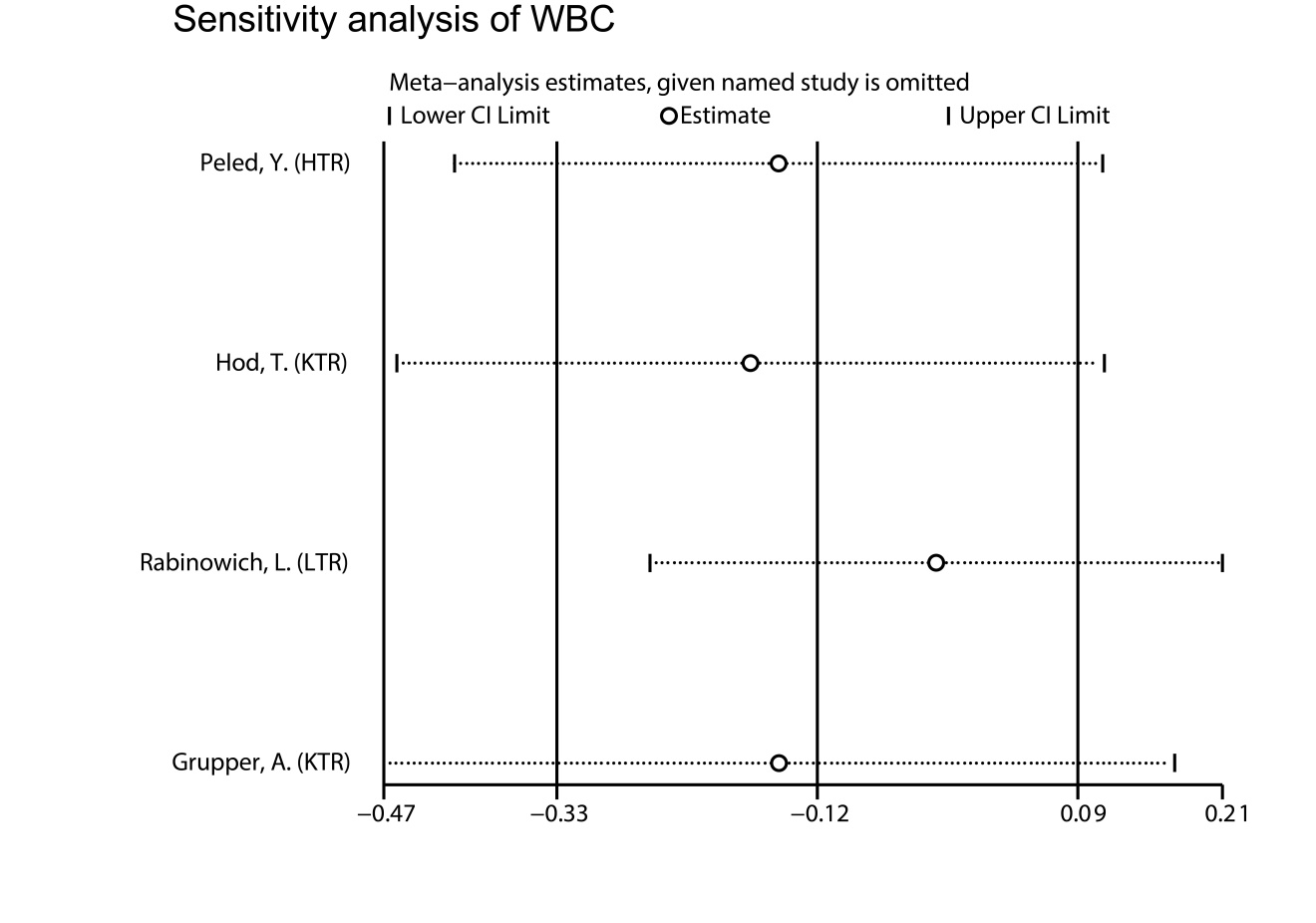

Supplement: Supplementary file 1 [file DataSheet_1.docx]
